# Dysbiosis-induced expansion of AXL-positive inflammatory type 3 dendritic cells triggers preclinical autoimmunity

**DOI:** 10.1038/s41590-026-02599-z

**Published:** 2026-07-21

**Authors:** Grozdan Cvijetic, Valentina Ottaviani, Isabella O. Conway, Erfan Jabari, Mladen Mitrovic, Haiting Wang, Patrick Fernandes Rodrigues, Anastasia du Halgouet, Siqi Zhao, Harrison C. Wang, Anjali P. Chandroth, Robert J. Palmer, Eduard Ansaldo, Andrew D. Doyle, Daniel Martin, Roman Szabo, Cristina Corsino, Francesca Pala, Miriam R. Fernandes, Nicolas Bouladoux, Howard A. Young, Yufan Zheng, Zhaoyuan Liu, Florent Ginhoux, Eric V. Dang, Stephen Darnell, George Liechti, Michail S. Lionakis, Robert Ivanek, Luigi D. Notarangelo, Yasmine Belkaid, WanJun Chen, Amiran Dzutsev, Giorgio Trinchieri, Roxane Tussiwand

**Affiliations:** 1 Immune Regulation Unit, National Institute of Dental and Craniofacial Research (NIDCR), National Institute of Health (NIH), Bethesda, MD USA; 2 Department of Biomedicine, University of Basel, Basel, Switzerland; 3 Mucosal Immunology Section, NIDCR, NIH, Bethesda, MD USA; 4 Department of Pathology and Immunology, Washington University School of Medicine in Saint Louis, Saint Louis, MO USA; 5 Structural Biochemistry Unit and Oral Immunity and Infection Section, NIDCR, NIH, Bethesda, MD USA; 6 Metaorganism Immunity Section, Laboratory of Host Microbiome and Immunity, National Institute of Allergy and Infectious Diseases (NIAID), NIH, Bethesda, MD USA; 7 NIDCR Imaging Core, NIDCR, NIH, Bethesda, MD USA; 8 Genomics and Computational Biology Core, NIDCR, NIH, Bethesda, MD USA; 9 Laboratory of Clinical Immunology and Microbiology, NIAID, NIH, Bethesda, MD USA; 10 Laboratory of Integrative Cancer Immunology, Center for Cancer Research, National Cancer Institute (NCI), NIH, Bethesda, MD USA; 11 Metaorganism Unit, Immunology Department, Institut Pasteur, Université Paris Cité, INSERM U1224, Paris, France; 12 Cancer Innovation Laboratory, Center for Cancer Research, NCI at Frederick, Frederick, MD USA; 13 Molecular Mycology and Immunity Unit, Laboratory of Host Microbiome and Immunity, NIAID, NIH, Bethesda, MD USA; 14 Ragon Institute of MGB, MIT, and Harvard, Cambridge, MA USA; 15 Shanghai Institute of Immunology, Department of Immunology and Microbiology, Shanghai Jiao Tong University School of Medicine, Shanghai, China; 16 Shanghai Key Laboratory for Tumor Microenvironment and Inflammation, Shanghai Jiao Tong University School of Medicine, Shanghai, China; 17 Gustave Roussy, Paris-Saclay University, Villejuif, France; Singapore Immunology Network (SIgN), A*STAR, Singapore, Singapore; 18 Department of Immunology, Harvard Medical School, Boston, MA USA; 19 Department of Microbiology and Immunology, Uniformed Services University of the Health Sciences, Bethesda, MD USA; 20 Henry M. Jackson Foundation for the Advancement of Military Medicine, Bethesda, MD USA; 21 Fungal Pathogenesis Section, Laboratory of Clinical Immunology and Microbiology, NIAID, NIH, Bethesda, MD USA; 22 Swiss Institute of Bioinformatics, Basel, Switzerland; 23 Immune Regulation, Life and Medical Sciences (LIMES) Institute, University of Bonn, Bonn, Germany

**Keywords:** Autoimmunity, Chronic inflammation, Mucosal immunology, Antibodies

## Abstract

Conventional dendritic cells (cDCs) are key sentinels at epithelial barriers, regulating immunity to microbial pathogens and commensals while preserving tissue integrity. NOTCH2 deficiency in CD11c-expressing cells (*Notch2*^cKO^) disrupts type 2a DC (cDC2a) development, impairs intestinal T_H_17 immunity and increases susceptibility to enteropathogenic bacteria. This defect leads to persistent dysbiosis in *Notch2*^cKO^ mice, characterized by low-grade inflammation and systemic autoimmune features, including elevated autoantibody titers and renal immune complex deposition. Dysbiosis precedes expansion of highly inflammatory AXL-expressing type 3 DCs (AXL^+^inf-DC3), promoting chronic inflammation and tertiary lymphoid structures driving adaptive immune responses. Notably, dysbiosis is defined by three dominant pathobionts and is transferable to wild-type mice, recapitulating the autoimmune features observed in *Notch2*^cKO^ mice. Here these findings identify a microbiota–DC axis linking intestinal pathobionts to systemic autoimmunity, establishing inflammatory DC3 as the cellular bridge between dysbiosis, chronic inflammation and autoimmune pathogenesis.

## Abstract (web summary)

Cvijetic et al. show Notch2 deficiency leads to altered populations of CD11c^+^ dendritic cells, including loss of ESAM^+^ cDC2a and an increase in inflammatory AXL^+^ DC3s, which sense and respond to gut dysbiosis, promoting autoantibody production.

## Main

Myeloid cells, including conventional dendritic cells (cDCs), are professional antigen presenting cells (APCs) and key sentinels at epithelial barriers. Equipped with pathogen- and danger-associated molecular pattern receptors, cDCs continuously sample the environment, regulating immune responses to microbial pathogens and commensals, thereby maintaining homeostasis under steady-state conditions^1,2^. Upon infection or tissue damage, DCs become activated, capture antigens, migrate to draining lymph nodes and secrete inflammatory cytokines that collectively shape adaptive immune responses^1,2^. DCs comprise myeloid-derived conventional DCs, which are further subdivided into type 1 (cDC1) and type 2 (cDC2) and lymphoid-derived plasmacytoid DCs (pDCs)^1^. Each subset performs a distinct but complementary function. pDCs are specialized for the rapid production of type I interferons and the amplification of systemic inflammation^3–6^, whereas cDCs act as professional APCs^1^. cDC1s efficiently produce interleukin-12 (IL-12), cross-present cell-associated antigens and promote cytotoxic CD8^+^ T cell responses against viruses, tumors and intracellular pathogens^7,8^. In contrast, cDC2s are phenotypically and functionally heterogeneous but are broadly defined by their expression of CD11b and CD172 (ref. ^9^) and their ability to prime CD4^+^ T cell responses^1,9^. Recent advances in high-dimensional cytometry and single-cell transcriptomics have revealed substantial diversity within the cDC2 compartment across tissues, developmental stages and inflammatory conditions^1,2,10–15^. At steady state, cDC2s comprise multiple transcriptionally and functionally distinct populations, including *Notch2*-dependent ESAM^+^ *Tbx21*-expressing cDC2a^16–20^ and the more heterogenous cDC2b that include partially overlapping subsets expressing CLEC12A, CLEC10A (MGL2)^18,20^ and the KLF4-dependent double negative DCs^6,21–23^. Additional DC subsets include the lymphoid-derived *Rorc-*expressing DCs^18,24–28^ and monocyte/DC progenitor-derived CD16/32^+^ type 3 DCs (DC3s)^29^, the latter showing partial transcriptional and phenotypic overlap with cDC2b and monocyte-derived DCs^18,20,29–31^. Each subset appears specialized in distinct immune responses, with partial redundancy ensuring the robustness of the system. Under inflammatory conditions, DCs upregulate activation markers and acquire overlapping features that blur lineage boundaries. In addition, monocyte-derived DCs can adopt classical DC markers, complicating the distinction between bona fide DCs and other mononuclear phagocytes. Several context-specific inflammatory DC states have been described, including inflammatory DC2 (inf-DC2) during viral respiratory infection^14^ and maturation-associated regulatory DCs within tumors^15,32^. Despite all advances, the developmental relationships and functional contributions of the different DC subsets, particularly DC3, remain incompletely understood.

NOTCH2 signaling plays a critical role in DC differentiation and is essential for the development of cDC2a^16,17^. However, given the broad expression of NOTCH2 across all DC subsets, it remained to be determined how its deficiency would affect developmental and functional features beyond the loss of cDC2a. We here analyzed the DC landscape in *Notch2*^cKO^ mice. In addition to the expected reduction of cDC2a, we observed a systemic expansion of AXL^+^ DC3s. These cells exhibited a pronounced inflammatory transcriptional signature and were therefore referred to as AXL^+^inf-DC3s. Notably, *Notch2*^cKO^ mice were also characterized by intestinal dysbiosis accompanied by multiple inflammatory features and signs of subclinical autoimmunity, including increased autoantibody titers. The abundance of AXL^+^inf-DC3s correlated with microbial dysbiosis and elevated autoantibody titers. To our surprise, dysbiosis was defined by the presence of three pathobionts and was transferable to wild-type (WT) mice. Further, colonization with these bacterial species induced the expansion of AXL^+^inf-DC3s and promoted similar autoimmune features, providing mechanistic insights into the pathogenesis of autoimmune diseases.

## Results

### *Notch2* deficiency alters all DC subsets

**Fig. 1:**
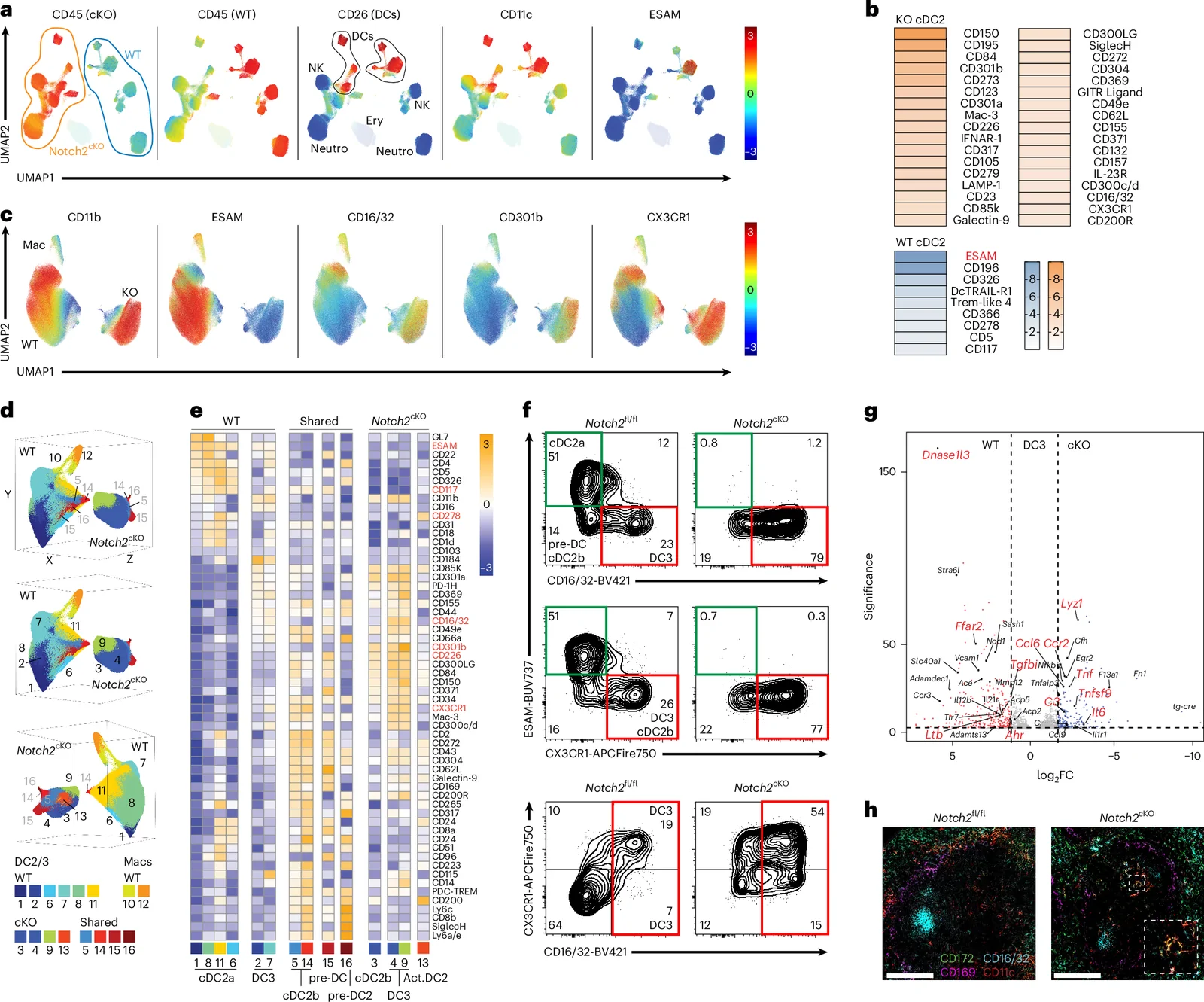
*Notch2* deficiency alters all DC subsets. **a**–**e**, Splenocytes from three *Notch2*^fl/fl^ (CD45-PerCP-Cy5.5) and three *Notch2*^cKO^ (CD45.2-AF700) mice were depleted of CD3^+^ and CD19^+^ cells, stained with a DC-specific backbone antibody panel and screened for 255 PE-conjugated individual antibodies provided by the LEGENDScreen kit. **a**, Uniform Manifold Approximation and Projection (UMAP) showing the relative expression levels of the indicated markers. **b**–**e**, Analysis of cDC2, pre-gated as in Extended Data Fig. 1b. **b**, Heatmap depicting differentially expressed proteins (fold change > 2) between genotypes on cDC2s. Orange shows upregulated genes in *Notch2*^cKO^ cDC2s; blue shows upregulated genes in *Notch2*^fl/fl^ cDC2s. **c**, UMAP of cDC2s illustrating the relative expression levels of selected surface markers. **d**, 3D-UMAP dimensionality reduction of cDC2s showing PhenoGraph-obtained clusters. The bottom panel represents a 180° rotation along the vertical axis. **e**, Relative expression level heatmap for cluster-defining markers. Values represent mean expression scaled per row (z-score). **f**, Flow cytometry plots showing splenic DC subsets, pre-gated as in Extended Data Fig. 1e, from *Notch2*^fl/fl^ and *Notch2*^cKO^ mice. Representative plots of three independent experiments (*n* = 3 mice). **g**, Bulk RNA-seq was performed on sort-purified DC3s and cDC1s from the spleen of *Notch2*^fl/fl^ and *Notch2*^cKO^ mice and cDC2a isolated from *Notch2*^fl/fl^ mice. Details are provided in Methods (*n* = 2 and *n* = 3 mice). Volcano plots showing the top 40 DEGs between *Notch2*^fl/fl^ and *Notch2*^cKO^ DC3. Significance is represented as -log_10_(adjusted *P* value). FC, fold change. **h**, Confocal fluorescence microscopy of DC populations in the spleens of *Notch2*^fl/fl^ and *Notch2*^cKO^ mice stained for CD169 (magenta), CD172 (green), CD16/32 (cyan) and CD11c (red). Scale bars: 150 μm. Dashed square indicates the magnified area. The images are representative of two independent experiments (each *n* = 3 mice). Source data

**Extended Data Fig. 1:**
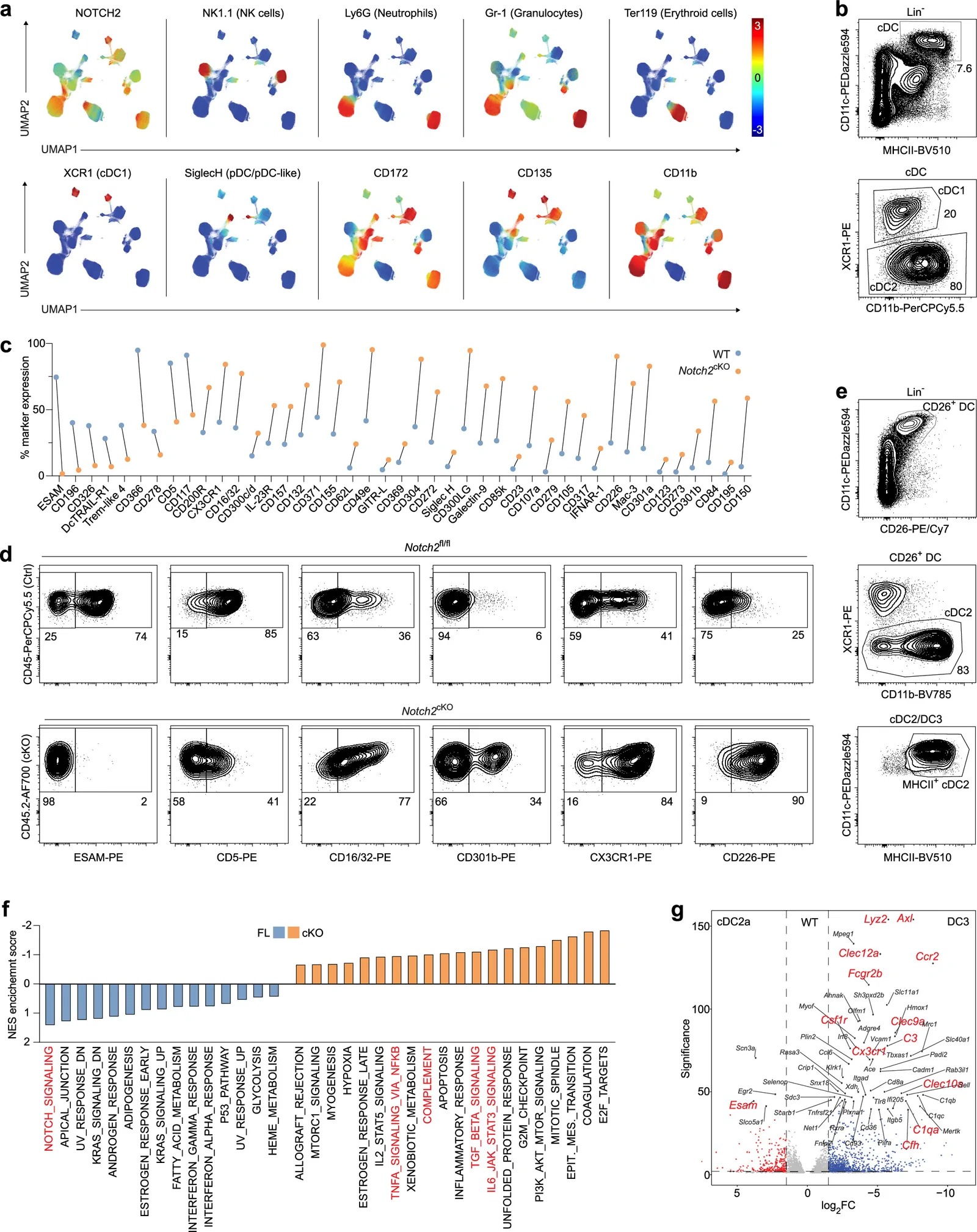
*Notch2* deficiency alters all DC subsets. Related to Fig. 1. (**a–d**) Cells were prepared as described in Fig. 1a. (**a**) UMAP showing the relative expression levels of the indicated markers in depleted splenocytes. (**b**) Flow cytometry plots showing the gating strategy for live Lin (CD3, CD19)^−^CD11c^+^MHC-II^+^XCR1^−^cDC2 and Lin^−^CD11c^+^MHC-II^+^XCR1^+^cDC1. (**c**) Flow cytometry-based frequencies of the indicated proteins in cDC2, showing a > 2-fold difference between *Notch2*^fl/fl^ (blue) and *Notch2*^cKO^ (orange) mice. (**d**) Representative flow cytometry plots of cluster-defining markers differentially expressed in cDC2 from *Notch2*^fl/fl^ (top row) and *Notch2*^cKO^ (bottom row) mice. (**e**) Flow cytometry plots showing the improved gating strategy for live Lin^−^CD26^+^CD11c^+^MHC-II^+^XCR1^−^cDC2. (**f**) Pathway analysis of DEGs in DC3 is plotted as enriched in *Notch2*^cKO^ (orange) versus *Notch2*^fl/fl^ (blue). The normalized enrichment score (NES) reflects the strength and direction of enrichment relative to background. (**g**) Bulk RNA-seq was performed as described in Fig. 1g. Volcano plots showing the top 40 DEGs between *Notch2*^fl/fl^ cDC2a and DC3. Significance is represented as -log_10_(adjusted *P* value). Source data

*Notch2*^cKO^ mice exhibit a profound reduction of the cDC2 compartment, with a near-complete loss of ESAM^+^ cDC2a^16,17^. To characterize changes that may have affected other subsets given the broad expression of NOTCH2 across all DCs, we performed LEGENDScreen profiling on CD3^−^CD19^−^-enriched splenocytes from *Notch2*^fl/fl^ (WT) and *Notch2*^fl/fl^*CD11c*^Cre^ (*Notch2*^cKO^/cKO) mice. Using InfinityFlow^30^, we analyzed surface expression for 255 markers (Fig. 1a and Extended Data Fig. 1a). Focusing on XCR1^−^ cDC2s (Extended Data Fig. 1b), we identified 44 that were differentially expressed, of which 35 were enriched in *Notch2*^cKO^ and 9 in WT cDC2s (Fig. 1b and Extended Data Fig. 1c,d). Unsupervised clustering analysis on the 173 expressed markers, but excluding CD45, highlighted substantial changes beyond the lack of cDC2a. We identified 16 clusters across cDC2s, including two MerTK^+^ macrophage-specific populations, that were absent in cKO cells because of reduced CD11c expression (Fig. 1c–e and Supplementary Fig. 1a). Of those, six were WT exclusive and included ESAM^+^ cDC2a (clusters 1, 8, 6 and 11) and ESAM^−^CD16/32^+^DC3 (clusters 2 and 7); four were shared and included CX3CR1^+^CD16/32^−^ cDC2b (clusters 5 and 14), CD226^+^ preDCs^33^ (cluster 15) and SiglecH^+^ pDC-like/pre-cDC2b cells^22,29^ (cluster 16); and four were cKO exclusive: cDC2b (cluster 3), DC3 (clusters 4 and 9) and activated CD278^hi^ (ICOS) CD200^hi^ cDC2 (cluster 13) (Fig. 1d,e). We validated key subsets by conventional flow cytometry, confirming increased DC3 frequencies, but not total numbers, in *Notch2*^cKO^ mice, unlike the previously reported reduction in *Klf4*^fl/fl^*CD11c*^Cre^ mice (Fig. 1f, Extended Data Fig. 1e and Supplementary Fig. 1b–d)^22,34^.

Bulk RNA sequencing (RNA-seq) on cDC1, cDC2a and DC3s highlighted profound transcriptional alterations (Fig. 1g, Supplementary Fig. 1e,f and Supplementary Table 1). Among the most differentially expressed genes (DEGs) across all subsets, we identified *Dnase1l3*, whose expression was highly impaired in cKO cells. Pairwise comparison of WT and cKO DC3s showed enrichment of inflammatory genes (Fig. 1g) and pathways (Extended Data Fig. 1f) in cKO cells, including *Il6*, *C3* and *Tnf*. Comparison between WT cDC2a and DC3s identified *Axl*, *Ccr2*, *Fcgr2b*, *Clec12a*, *C3* and *Lyz2* as the most DEGs in DC3s (Extended Data Fig. 1g). Confocal microscopy revealed accumulation of *Notch2*^cKO^ DC3s within splenic B cell follicles in proximity of CD11c^−^CD16/32^+^ follicular DCs (FDCs), whereas WT DC3 localized to the marginal zone (Fig. 1h and Supplementary Fig. 1g–i), reminiscent of DC positioning described following immunization^35^.

Altogether, these results suggest that NOTCH2 deficiency impairs cDC2a development^16,17^, but also profoundly alters the transcriptional profile of other DC subsets, leading to an increased frequency of DC3 cells characterized by a proinflammatory state.

### Proinflammatory AXL^+^inf-DC3s systemically expand in *Notch2*^cKO^ mice

**Fig. 2:**
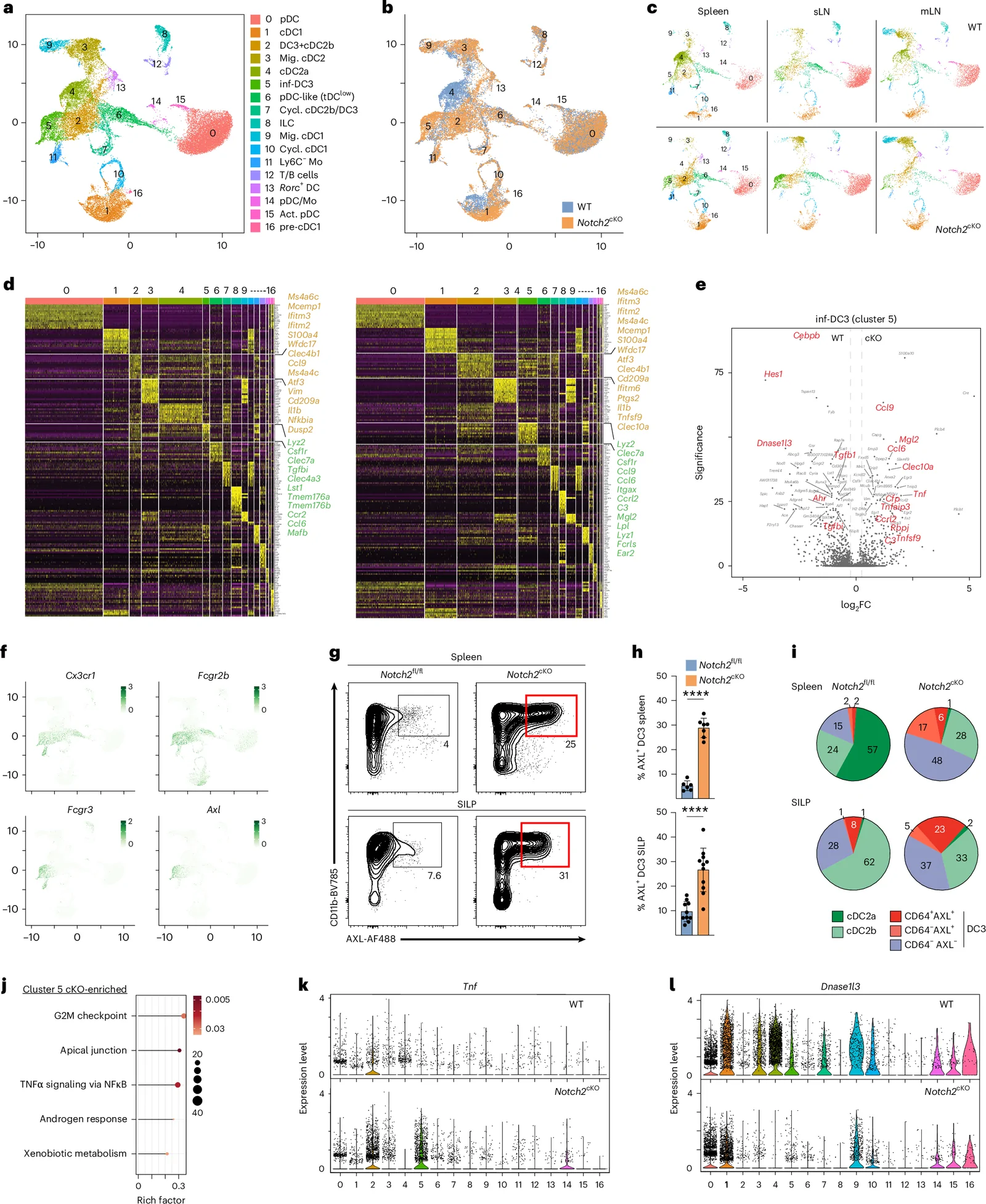
Proinflammatory AXL^+^inf-DC3s systemically expand in *Notch2*^cKO^ mice. **a**–**f**, scRNA-seq was performed on CD11c^+^-enriched and sort-purified DCs from spleen, sLN and mLN from three C57BL/6 (WT) and *Notch2*^cKO^ mice. Details are provided in Methods. **a**, UMAP showing cluster distribution of cells from each genotype and tissue. Mig. DC, migratory DCs; Cycl. DC, cycling DCs; Act. DC, activated DCs; Mo, monocytes. **b**, UMAP showing cells colored by genotype. **c**, UMAP showing cluster distribution for each tissue and genotype separated. **d**, Heatmaps of relative expression levels showing the top 15 DEGs per cluster for WT (left) and *Notch2*^cKO^ (right). **e**, Volcano plot showing the top 80 DEGs between WT and *Notch2*^cKO^ cluster 5 (inf-DC3). Significance is represented as −log_10_(adjusted *P* value). **f**, UMAP showing relative expression levels of the indicated DC2 and DC3 genes across all genotypes and tissues combined. **g**–**i**, Analysis of AXL expression within DC2 and DC3, pre-gated as in Extended Data Fig. 2g, of spleen and SILP of *Notch2*^fl/fl^ and *Notch2*^cKO^ mice. Shown are the flow cytometry plots (**g**) and frequencies (**h**) for AXL^+^inf-DC3 (spleen: *n* = 6 and 7 mice, SILP: *n* = 10 and 11 mice, pooled data from three independent experiments). **i**, Pie charts showing average DC2 and DC3 subset frequencies in spleen and SILP of *Notch2*^fl/fl^ and *Notch2*^cKO^. Data are shown as mean ± standard deviation (s.d.). Statistical analysis was performed using an unpaired two-tailed *t*-test. ****P > 0.0001. **j**, Pathway enrichment analysis based on DEGs between WT and *Notch2*^cKO^ cluster 5 (inf-DC3). The cKO-enriched pathways are displayed as dot plot. GSEA was performed using the Molecular Signatures Database (V7.5.1). Dot size represents the number of core enriched genes. Color indicates the adjusted *P* value. The rich factor represents the ratio of core enriched genes to the total number of genes in the gene set. **k**,**l**, Violin plots showing log-normalized gene expression levels for *Tnf* (**k**) and *Dnase1l3* (**l**) across all clusters in WT (top) and *Notch2*^cKO^ (bottom). Source data

**Extended Data Fig. 2:**
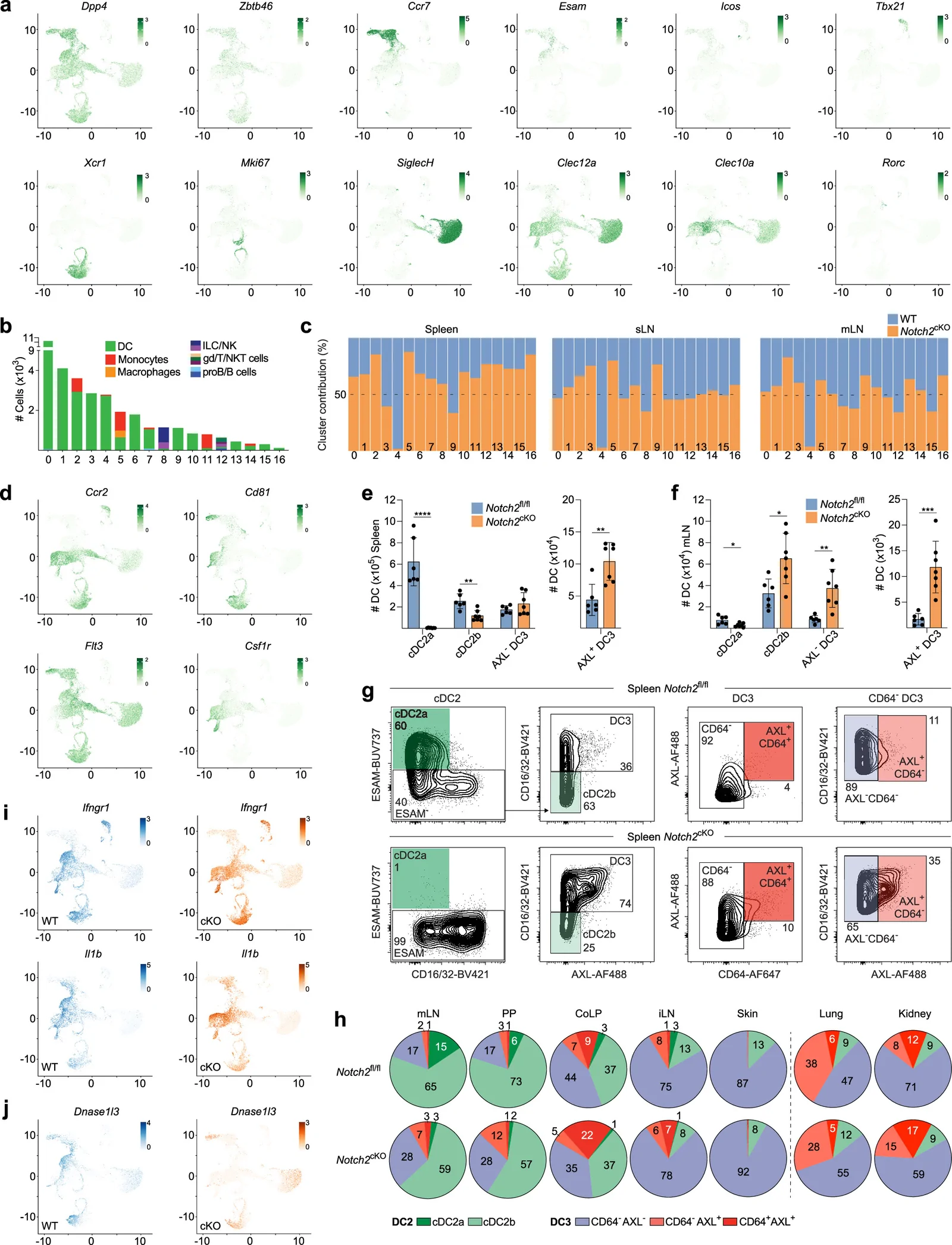
Proinflammatory AXL^+^inf-DC3 systemically expand in *Notch2*^cKO^ mice. Related to Fig. 2. (**a–d**) Cells were prepared as described in Fig. 2a. (**a**) UMAP showing relative expression levels of the indicated genes including cells from each genotype and tissue. (**b**) Quantification of cell-type identity in each cluster using reference profiles from the ImmGen database. (**c**) Contribution frequency of each genotype to the overall cluster composition is plotted for each tissue. (**d**) UMAP displaying the relative expression levels of the indicated genes across all genotypes and tissues combined. (**e, f**) Shown are the total cell numbers for the indicated DC2 and DC3 subsets in spleen (**e**) and mLN (**f**). (*n* = 6 and 7 mice, pooled data from three independent experiments). (**g**) Flow cytometry plots showing the gating strategy for DC2/DC3 (pre-gated as Lin^−^CD26^+^CD11c^+^MHC-II^+^XCR1^−^) subsets including AXL and CD64 in *Notch2*^fl/fl^ and *Notch2*^cKO^ spleen. Representative plots of three independent experiments. (**h**) Pie charts showing average frequencies of DC2 and DC3 subsets in mLN, Peyer’s Patches (PP), CoLP, inguinal lymph node (iLN), dorsal skin, lung, and kidney of *Notch2*^fl/fl^ and *Notch2*^cKO^. (*n* = 6 and 7 mice, pooled data from two to three independent experiments). (**i, j**) Cells were prepared as described in Fig. 2a. (**i**) UMAP illustrating relative expression levels of the indicated genes in WT (blue) and *Notch2*^cKO^ (orange). (**j**) UMAP showing relative expression of *Dnase1l3* in WT (blue) and *Notch2*^cKO^ (orange). Data are shown as mean ± SD. Statistical analysis was performed using an unpaired two-tailed *t*-test. *P > 0.05, **P > 0.01, ***P > 0.001, ****P > 0.0001. Source data

To gain more analytical depth of the differences across subsets, we performed scRNA-seq on Lin^−^CD11c^+^cells isolated from spleen, mesenteric lymph nodes (mLN) and skin-draining LN (sLN). We identified 17 clusters across WT and *Notch2*^cKO^ cells (Fig. 2a). Clusters were assigned based on transcriptional profiles and validated by selected marker expression. DC signature was confirmed using cell-type enrichment analysis as well as *Dpp4, Flt3* and *Zbtb46* expression (Extended Data Fig. 2a,b and Supplementary Fig. 2a). As expected, *Esam*^+^ cDC2a (cluster 4) was absent in *Notch2*^cKO^ mice, and consistent with the above results, major differences characterized cKO mice (Fig. 2b and Supplementary Fig. 2b). Most notable was the expansion of clusters 5 and 2 in *Notch2*^cKO^ mice (Fig. 2c and Extended Data Fig. 2c) and the inflammatory signature of cluster 5 cKO cells, as determined by the top 15 DEG across all clusters and pairwise comparison between WT and cKO cells (Fig. 2d,e, Supplementary Fig. 2b,c and Supplementary Tables 2 and 3). The inflammatory profile of cluster 5 was characterized by high expression of *Tnf*, *Ccl6*, *Ccl9* and *Ccrl2*, complement genes *C3* and *Cfp*, antimicrobial genes *Lyz1* and *Lyz2* and activation genes *Tgfbi* and *Lpl*, among others. Taking advantage of *Cx3cr1* and *Fcgr3*/*Fcgr2b*, we could classify clusters 5 and 2 as DC3s (Fig. 2f, Extended Data Fig. 2d and Supplementary Fig. 2d). Further, we could segregate those clusters by transcript and protein expression of the scavenger receptor *Axl* (Fig. 2f,g) and refer hereafter to cluster 5 as AXL^+^inf-DC3. This subset could be further split based on CD64 (*Fcgr1*) expression and was markedly expanded across most tissues in *Notch2*^cKO^ mice, accounting for almost a third of all DCs within spleen and intestinal tissues, that is, small intestine lamina propria (SILP) and colon LP (CoLP) (Fig. 2g–i, Extended Data Fig. 2e–h). Across tissues, AXL^+^inf-DC3s preserved a core signature characterized by inflammatory and proliferation-associated genes, as well as transcripts classically associated with monocytes and macrophages, such as *Lyz2*, *Clec7a* and *Csf1r* (Fig. 2d and Supplementary Fig. 2d). Differential expression and pathway analysis between WT and cKO cluster 5 highlighted transcripts related to inflammation, antibacterial defense, migration, redox regulation and antigen processing and presentation (Fig. 2e,j,k, Extended Data Fig. 2i and Supplementary Fig. 2b–e), suggesting a functional role in regulating epithelial responses, possibly to the microbiome or pathobiome. In contrast, the recently identified *Rorc*-expressing DCs (cluster 13), shown to regulate immunity to orally derived antigens^18,24–26,28^, appeared preserved in numbers and transcriptionally unaltered (Supplementary Fig. 2f).

Collectively, *Notch2* deficiency impaired cDC2a development, broadly altered the transcriptional landscape of DC subsets and induced systemic expansion of AXL^+^inf-DC3s across multiple tissues. We identified *Dnase1l3* as one of the most dysregulated genes across all subsets (Fig. 2l and Extended Data Fig. 2j).

### *Notch2*^cKO^ mice show autoimmune features

**Fig. 3:**
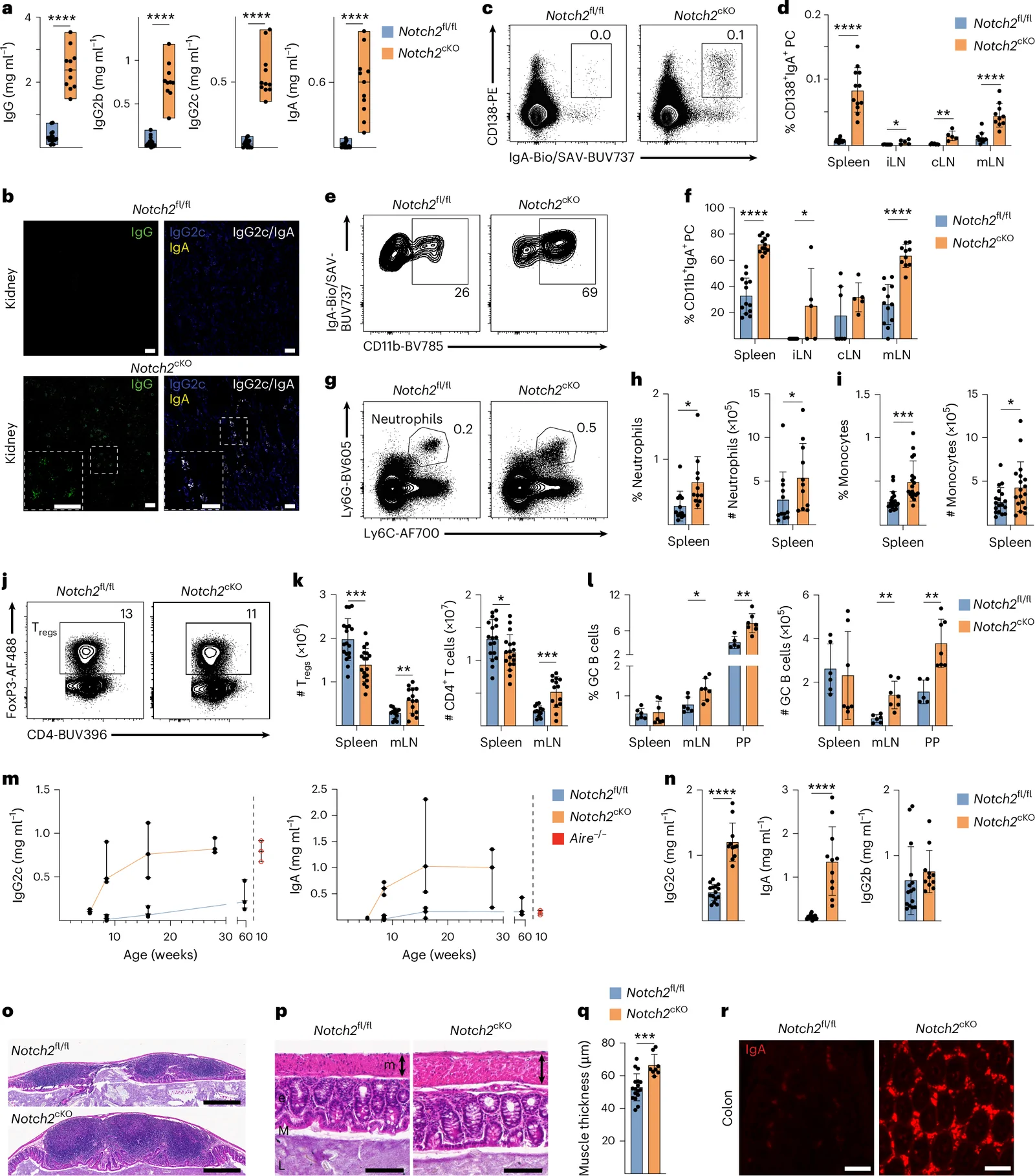
*Notch2*^cKO^ mice show autoimmune features. **a**, Serum immunoglobulin concentrations in 8- to 12-week-old *Notch2*^fl/fl^ (blue) and *Notch2*^cKO^ (orange) mice were determined by enzyme-linked immunosorbent assay (ELISA) (IgG/IgG2c/IgA: *n* = 16 and 11 mice, IgG2b: *n* = 15 and 10 mice, pooled data from at least three independent experiments). **b**, Confocal fluorescence microscopy of immune complex deposition in kidneys of 16-week-old *Notch2*^fl/fl^ (top) and *Notch2*^cKO^ (bottom), stained for IgG (green) (left), IgG2c (blue) and IgA (yellow) (right). Scale bars: 150 μm. Dashed square indicates the magnified area. The images are representative of two independent experiments (each *n* = 3). **c**,**d**, IgA^+^CD138^+^ PCs were assessed in *Notch2*^fl/fl^ (blue) and *Notch2*^cKO^ (orange) mice. Representative flow cytometry plots (**c**) and frequencies across the indicated tissues (**d**) are shown (spleen: *n* = 13 and 12 mice, iLN/cervical lymph node (cLN): *n* = 7 and 5 mice, mLN: *n* = 12 and 10 mice, pooled data from two to four independent experiments). **e**,**f**, CD11b expression within IgA^+^CD138^+^ PCs was assessed in *Notch2*^fl/fl^ (blue) and *Notch2*^cKO^ (orange) mice. Representative flow cytometry plots (**e**) and frequencies across the indicted tissues (**f**) are shown (spleen: *n* = 13 and 12 mice, iLN, cLN: *n* = 7 and 5 mice, mLN: *n* = 12 and 10 mice, pooled data from two to four independent experiments). **g**–**l**, Flow cytometric analysis of immune cells from 8- to 12-week-old *Notch2*^fl/fl^ (blue) and *Notch2*^cKO^ (orange) mice. **g**,**h**, Ly6G^+^Ly6C^+^ neutrophils within live cells were assessed in the spleen. Shown are representative flow cytometry plots (**g**), frequencies and total cell numbers (**h**) (spleen: *n* = 12 and 11 mice, pooled data from two to four independent experiments). **i**, CX3CR1^+^Ly6C^+^ monocytes within live cells were assessed in the spleen. Shown are frequencies (left) and total cell numbers (right) (spleen: *n* = 18 mice, pooled data from four to five independent experiments). **j**,**k**, FoxP3^+^ T_reg_ cells within CD4^+^ T cells (CD19^−^CD3^+^CD8^−^) and FoxP3^−^CD4^+^ T cells were assessed across the indicated tissues. Shown are representative flow cytometry plots (**j**) and total numbers (**k**) (spleen: *n* = 18 mice, mLN: *n* = 12 and 14 mice, pooled data from two to five independent experiments). **l**, CD95^+^GL7^+^ GCs within CD19^+^B220^+^ B cells were assessed across the indicated tissues. Shown are frequencies (left) and total cell numbers (right) (spleen/mLN: *n* = 6 and 7 mice, PP: *n* = 5 and 7, pooled data from two to three independent experiments). **m**, Serum immunoglobulin concentrations at the indicated timepoints in *Notch2*^fl/fl^ (blue), *Notch2*^cKO^ (orange) and the autoimmune reference *Aire*^−/−^ (red) mice were determined by ELISA (*n* = 3 mice per timepoint, representative of at least three independent collections, quantifications and including the range of young serum Ig levels shown in Fig. 3a as reference). **n**, Serum immunoglobulin concentrations in 70-week-old *Notch2*^fl/fl^ (blue) and *Notch2*^cKO^ (orange) mice were measured by ELISA (*n* = 15 and 11 mice, pooled data from three independent experiments). **o**, H&E staining of cecal patches from young *Notch2*^fl/fl^ and *Notch2*^cKO^. Scale bars: 500 μm. The images are representative of two independent experiments (each *n* = 3). **p**–**q**, Muscle thickness in H&E-stained colons from aged *Notch2*^fl/fl^ and *Notch2*^cKO^ mice. Representative images (**p**), and quantification (**q**). m, muscle; e, epithelium; M, mucus; L, lumen. Arrows indicate muscle thickness. Scale bars: 250 μm (*n* = 17 and 8 mice, pooled data and representative images of two independent experiments). **r**, Confocal fluorescence microscopy of intraepithelial IgA (red) in FFPE colon tissue from aged *Notch2*^fl/fl^ and *Notch2*^cKO^. Scale bars: 50 μm. The images are representative of two independent experiments (each *n* = 3). Data in bar graphs are shown as mean ± s.d. Floating bar plots show the median (center line), with bars extending from the minimum to the maximum value. Statistical analysis was performed using an unpaired two-tailed *t*-test, except panel h (right), where a two-sided Mann-Whitney U test was applied. *P > 0.05, **P > 0.01, ***P > 0.001, ****P > 0.0001. Source data

**Extended Data Fig. 3:**
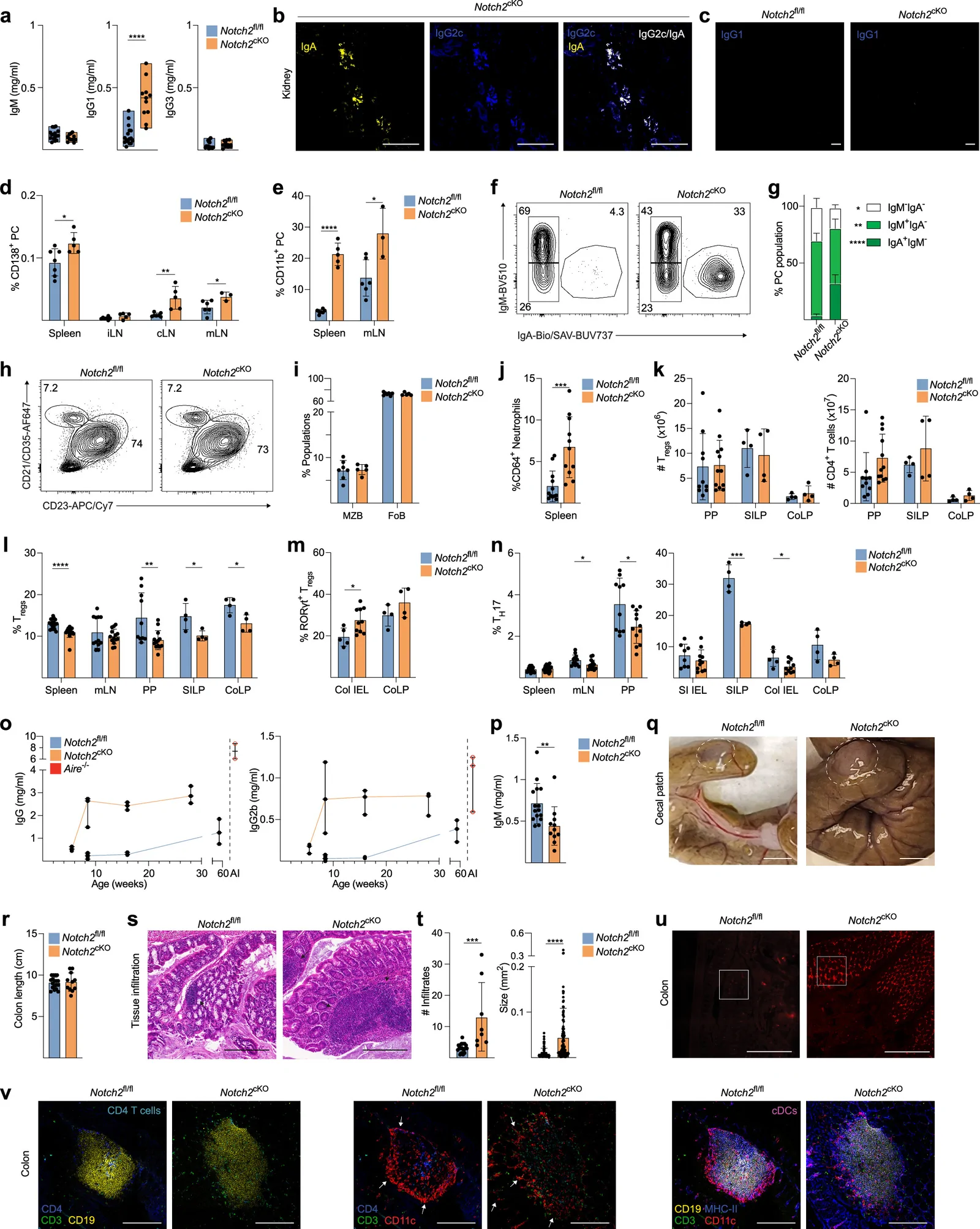
*Notch2*^cKO^ mice show autoimmune features. Related to Fig. 3. (**a**) Serum immunoglobulin concentrations in 8–12-week-old *Notch2*^fl/fl^ (blue) and *Notch2*^cKO^ (orange) mice were determined by ELISA. (*n* = 16 and 11 mice, pooled data from three independent experiments). (**b, c**) Confocal microscopy of kidney immune complex deposition in 16-week-old mice. (**b**) Representative IgA (yellow, left), IgG2c (blue, middle), and merged (right) images from *Notch2*^cKO^. Images correspond to Fig. 3b. (**c**) IgG1 staining in *Notch2*^fl/fl^ and *Notch2*^cKO^. Scale bars: 150 μm. The images are representative of two independent experiments (each *n* = 3 mice). (**d, e**) Frequency of CD138^+^ PCs (**d**) and CD11b expression within CD138^+^ PCs (**e**) in the indicated tissues of *Notch2*^fl/fl^ and *Notch2*^cKO^ mice. (Spleen/iLN/cLN: *n* = 7 and 5 mice, mLN: *n* = 6 and 3 mice, pooled data from two independent experiments). (**f, g**) Representative flow cytometry plots (**f**) and frequencies (**g**) of PC subsets based on IgM and IgA expression within CD138^+^ PCs were quantified in *Notch2*^fl/fl^ and *Notch2*^cKO^ mice. (*n* = 7 and 5 mice, pooled data from two independent experiments). (**h, i**) Representative plots (**h**) and frequencies (**i**) of CD21/CD35^+^CD23^+^ follicular (FoB) and CD21/CD35^+^CD23^−^marginal zone (MZB) B cells among splenic CD19^+^B220^+^ B cells. (*n* = 7 and 5 mice, pooled data from two independent experiments). (**j**) Frequency of CD64 expression within splenic Ly6G^+^Ly6C^+^ neutrophils. (*n* = 12 and 11 mice, pooled data from two independent experiments). (**k**) Total cell numbers of FoxP3^+^ (left) and FoxP3^−^(right) CD4^+^ T cells, pre-gated as in 3j, across the indicated tissues. (PP: *n* = 10 and 12 mice, T_reg_ SILP/CoLP: *n* = 4 mice, pooled data from two to five independent experiments). (**l**) Frequency of FoxP3^+^ T_reg_ within CD4^+^ T cells, pre-gated as in 3j, across the indicated tissues. (Spleen: *n* = 18 mice, mLN: *n* = 12 and 14 mice, PP: *n* = 10 and 13 mice, SILP/CoLP: *n* = 4 mice, pooled data from two to five independent experiments). (**m-n**) Frequency of RORγt expression within FoxP3^+^ (**m**), or FoxP3^−^ (**n**) CD4^+^T cells, pre-gated as in 3j, across the indicated tissues. (Spleen: *n* = 18 and 19 mice, mLN: *n* = 12 and 14 mice, PP: *n* = 10 and 12 mice, SILP/CoLP: *n* = 4 mice, SI IEL: *n* = 8 and 11 mice, Col IEL: *n* = 5 and 10 mice, CoLP: *n* = 4 mice, pooled data from two to three independent experiments). (**o**) Serum immunoglobulin concentrations at the indicated timepoints. (*n* = 3 mice per timepoint, *Aire*^−/−^: *n* = 2 or 3 mice, representative of at least three independent collections and quantifications; young mouse serum Ig range from Fig. 3a is included as reference). (**p**) Serum IgM concentration in 70-week-old mice. (*n* = 15 and 11 mice, pooled data from three independent experiments). (**q**) Cecal patches in young mice. Scale bars: 0.2 cm. Dashed circles highlight the cecal patch. The images are representative of two independent experiments (each *n* = 3 mice). (**r–u**) Tissue phenotypes in 70-week-old mice including colon length (**r**), representative H&E-stained colon sections (s), and tissue infiltrates (t). (**s**) Scale bars: 250 μm. Arrows indicate tissue infiltration. (**t**, left) Number of tissue infiltrates. (**t**, right) Infiltrate sizes. Each dot represents the measured size of an identified tissue infiltrate. (*n* = 18 and 11 mice, pooled data and representative images of two independent experiments). (**u**) Confocal microscopy of intraepithelial IgA (red) stained FFPE colon. Images correspond to Fig. 3r. Scale bars: 400 μm. The images are representative of two independent experiments (each *n* = 3 mice). (**v**) Confocal microscopy of immune cell localization in the colon of 25-30-week-old mice. Sections from each genotype were stained with CD3 (green), CD4 (blue), CD19 (yellow), CD11c (red), MHC-II (blue) and presented with the indicated channel combinations. Scale bars: 200 μm. Arrows indicate CD11c tissue distribution. The images are representative of two independent experiments (each *n* = 3 mice). Data in bar graphs are shown as mean ± SD. Floating bar plots show the median (center line), with bars extending from the minimum to the maximum value. Statistical analysis was performed using an unpaired two-tailed *t*-test. *P > 0.05, **P > 0.01, ***P > 0.001, ****P > 0.0001. Source data

Consistent with the previously reported autoimmune phenotype of *Dnase1l3*-deficient mice^36^, *Notch2*^cKO^ mice exhibited increased serum IgG1, IgG2b, IgG2c and IgA (Fig. 3a and Extended Data Fig. 3a), as well as immune complex deposition in the kidney (Fig. 3b and Extended Data Fig. 3b,c). This was consistent with a systemic accumulation of plasma cells (PCs), including IgA^+^ PCs, across all tissues analyzed (Fig. 3c–f and Extended Data Fig. 3d–g). Multiple features, including CD11b expression on PCs^37^ (Fig. 3e,f and Extended Data Fig. 3e), and isotype prevalence were consistent with an antibacterial polysaccharide immune response ongoing in cKO mice^38^. We could exclude impairment of marginal zone B cell development^39^ (Extended Data Fig. 3h,i). Instead, CD64^+^ neutrophils, previously described in the context of sepsis^40^, accumulated in the spleen, further supporting our hypothesis (Fig. 3g,h and Extended Data Fig. 3j). Similarly, frequency of monocytes increased in the spleen, suggesting a systemic reaction (Fig. 3i). Within the T cell compartment, regulatory T cells (T_reg_ cells) were reduced in the spleen but enriched in mLNs (Fig. 3j,k and Extended Data Fig. 3k–m), whereas T_H_17 cells appeared impaired within multiple gut-associated tissues, including SILP, as previously reported^16^ (Extended Data Fig. 3n). Taken together, *Notch2*^cKO^ mice exhibited enhanced antibody production and increased germinal center (GC) B cell reactions in mLN and Peyer’s patches (PP), compatible with an intestinal barrier defect (Fig. 3l), potentially related to their inability to mount effective T_H_17 immunity.

When monitored longitudinally, up to 70 weeks of age, antibody titers reached peak levels by 10 weeks, comparable to those observed in the autoimmune regulator *Aire*^−/−^ model (Fig. 3m,n and Extended Data Fig. 3o,p). However, *Notch2*^cKO^ mice displayed elevated levels of both IgG and IgA, suggesting a different pathogenic process from that observed in *Aire*^−/−^ or aged mice^41,42^, which exhibit increased IgG, but not IgA titers, thereby arguing against premature inflammaging as the primary driver (Fig. 3n and Extended Data Fig. 3p). Consistent with impaired intestinal barrier function, *Notch2*^cKO^ mice showed enlarged cecal patches, increased colonic muscle thickness, extensive immune cell infiltration and pronounced intraepithelial IgA accumulation around colonic crypts (Fig. 3o–r and Extended Data Fig. 3q–u). Immune cell infiltrates in the colon formed tertiary lymphoid structures containing organized CD4 T cell-DC aggregates, whereas colonic epithelial cells exhibited elevated MHC-II expression (Extended Data Fig. 3v), indicative of an ongoing inflammation.

Collectively, *Notch2*^cKO^ mice displayed an expansion of AXL^+^inf-DC3s, elevated systemic antibody titers, renal immune complex deposition and multiple features of intestinal inflammation consistent with dysbiosis-driven immune dysregulation.

### *Notch2*^cKO^ mice have dysbiosis

**Fig. 4:**
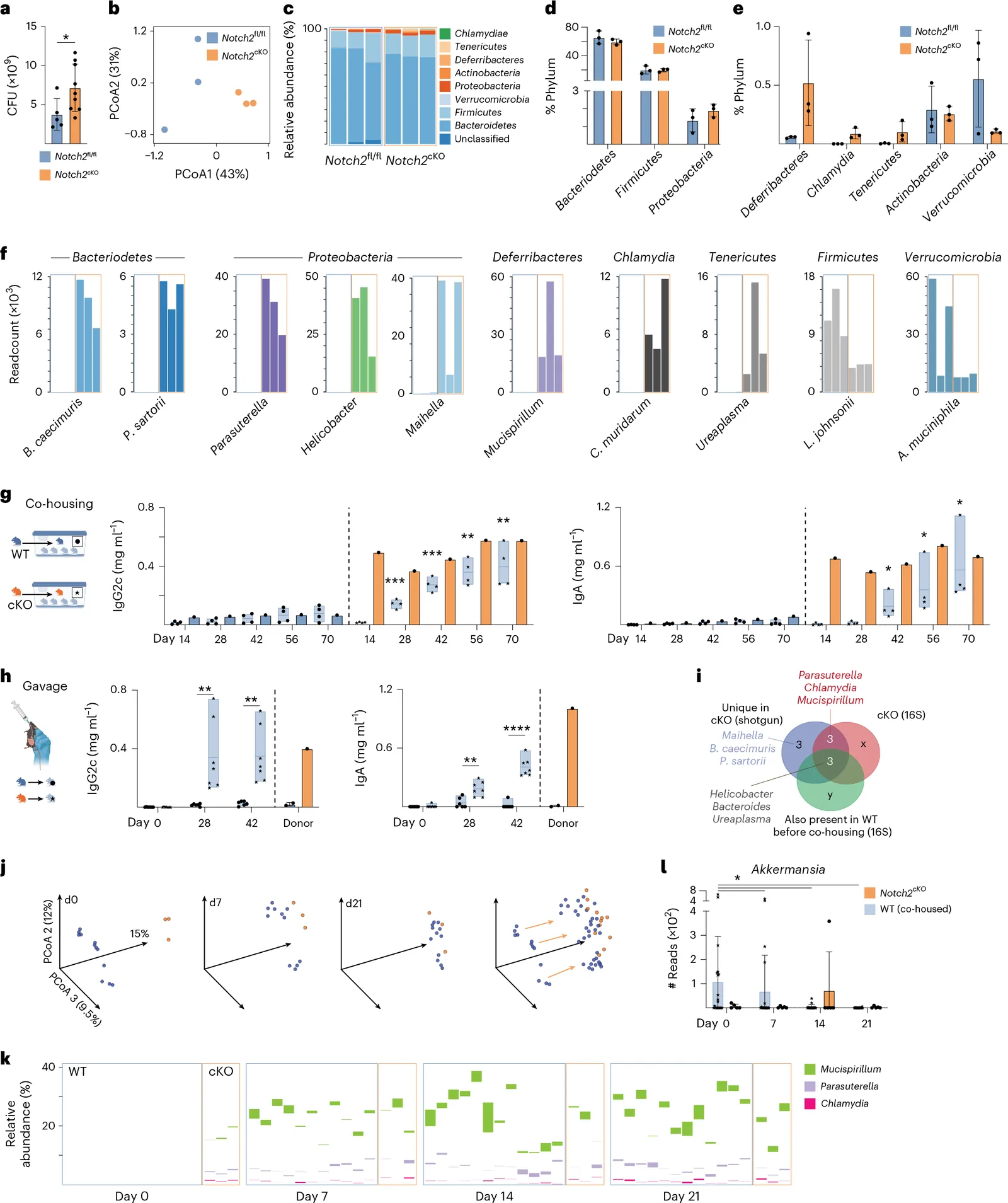
*Notch2*^cKO^ mice have intestinal dysbiosis that can be transferred. **a**–**f**, Fresh feces from young 8- to 12-week-old *Notch2*^fl/fl^ and *Notch2*^cKO^ mice were analyzed. **a**, Colony-forming units of fecal homogenates from *Notch2*^fl/fl^ and *Notch2*^cKO^ mice incubated on red blood agar plates under anaerobic conditions (*n* = 5 and 9 mice, pooled data from two independent experiments). **b**–**f**, Shotgun sequencing on fresh feces from three separately housed *Notch2*^fl/fl^ and three separately housed *Notch2*^cKO^ mice (*n* = 3 mice). **b**, Principal coordinate analysis (PCoA) plot of Jaccard distances at the species level based on shotgun metagenomic profiles of mice from each genotype. Percentages represent the variance explained by each principal coordinate. **c**, Relative abundance of bacterial phyla. **d**,**e**, Frequencies of phyla with high abundance (**d**) and low abundance (**e**). **f**, Total number of reads for the bacterial species *Bacteroides caecimuris, Phocaeicola sartorii, Parasutterella MGBC134540, Helicobacter MGBC103445, Maihella MGBC107932, Mucispirillum MGBC140179, Chlamydia muridarum, Ureaplasma MGBC165308*, *Lactobacillus johnsonii, Akkermansia muciniphila* in *Notch2*^fl/fl^ (blue square) and *Notch2*^cKO^ (orange square) and their corresponding phyla is indicated. **g**, Co-housing using four C57BL/6 mice (6–8 weeks old) and either one *Notch2*^fl/fl^ or *Notch2*^cKO^ mouse. Blood was collected biweekly for 70 days. Serum immunoglobulin concentrations were determined for each timepoint by ELISA. C57BL/6 co-housed with *Notch2*^fl/fl^ (light blue, circle symbol), *Notch2*^fl/fl^ (dark blue), C57BL/6* co-housed with *Notch2*^cKO^ (light blue, star symbol) and *Notch2*^cKO^ (orange). Statistical analysis was performed between the WT groups (recipient *n* = 4 mice per experiment, donor: *n* = 1 mouse per experiment, representative data from three independent experiments). **h**, C57BL/6 mice (6–8 weeks old) were gavaged with fecal homogenates (OD_600_ = 20) derived from either *Notch2*^fl/fl^ or *Notch2*^cKO^ mice. Blood was collected biweekly for 42 days, and serum immunoglobulin concentrations were determined by ELISA. C57BL/6 gavaged with *Notch2*^fl/fl^ fecal material (light blue, circle symbol), C57BL/6* gavaged with *Notch2*^cKO^ fecal material (light blue, star symbol), control *Notch2*^fl/fl^ (dark blue) and control *Notch2*^cKO^ (orange) (recipient: *n* = 6 and 7 mice, donor: *n* = 2 mice and 1 mouse, pooled data from two independent experiments). **i**−**l**, 16S rRNA sequencing of fecal material from co-housed 6- to 8-week-old *Notch2*^cKO^ (orange) and C57BL/6 (blue) mice. Fecal material was collected weekly over 21 days (C57BL/6: *n* = 20 mice, *Notch2*^cKO^: *n* = 5 mice, data from one or both independent experiments are shown as indicated). **i**, Venn diagram depicting bacterial species detected comparing those exclusively identified in *Notch2*^cKO^ by shotgun sequencing, those identified in *Notch2*^cKO^ at steady state by 16S sequencing and those identified in WT control feces at steady state by 16S sequencing. Numbers indicate the bacteria associated with each condition; 9 species were found by shotgun sequencing of *Notch2*^cKO^ mice. The numbers of uniquely identified bacterial species across 16S experiments are not indicated. **j**, 3D-PCoA plots of Jaccard distances at the species level based on 16S rRNA profiles from mice of each genotype at d0, d7 and d21. Percentages represent the variance explained by each PC. **k**, Relative abundance of *Mucispirillum*, *Parasutterella* and *Chlamydia* in feces of C57BL/6 (blue square) and *Notch2*^cKO^ (orange square) before and after co-housing at the indicated timepoints (C57BL/6: *n* = 12 mice, *Notch2*^cKO^: *n* = 3 mice, representative data from two independent experiments are shown). **l**, Number of *Akkermansia* reads in *Notch2*^cKO^ and C57BL/6 co-housed mice at the indicated timepoints (C57BL/6: *n* = 20 mice, *Notch2*^cKO^: *n* = 5 mice, pooled data from two independent experiments). Data in bar graphs are shown as mean ± s.d. Floating bar plots show the median (center line), with bars extending from the minimum to the maximum value. Statistical analysis was performed using an unpaired two-tailed *t*-test, except panel k, where a paired two-tailed *t*-test was applied. *P > 0.05, **P > 0.01, ****P > 0.0001. Source data

**Extended Data Fig. 4:**
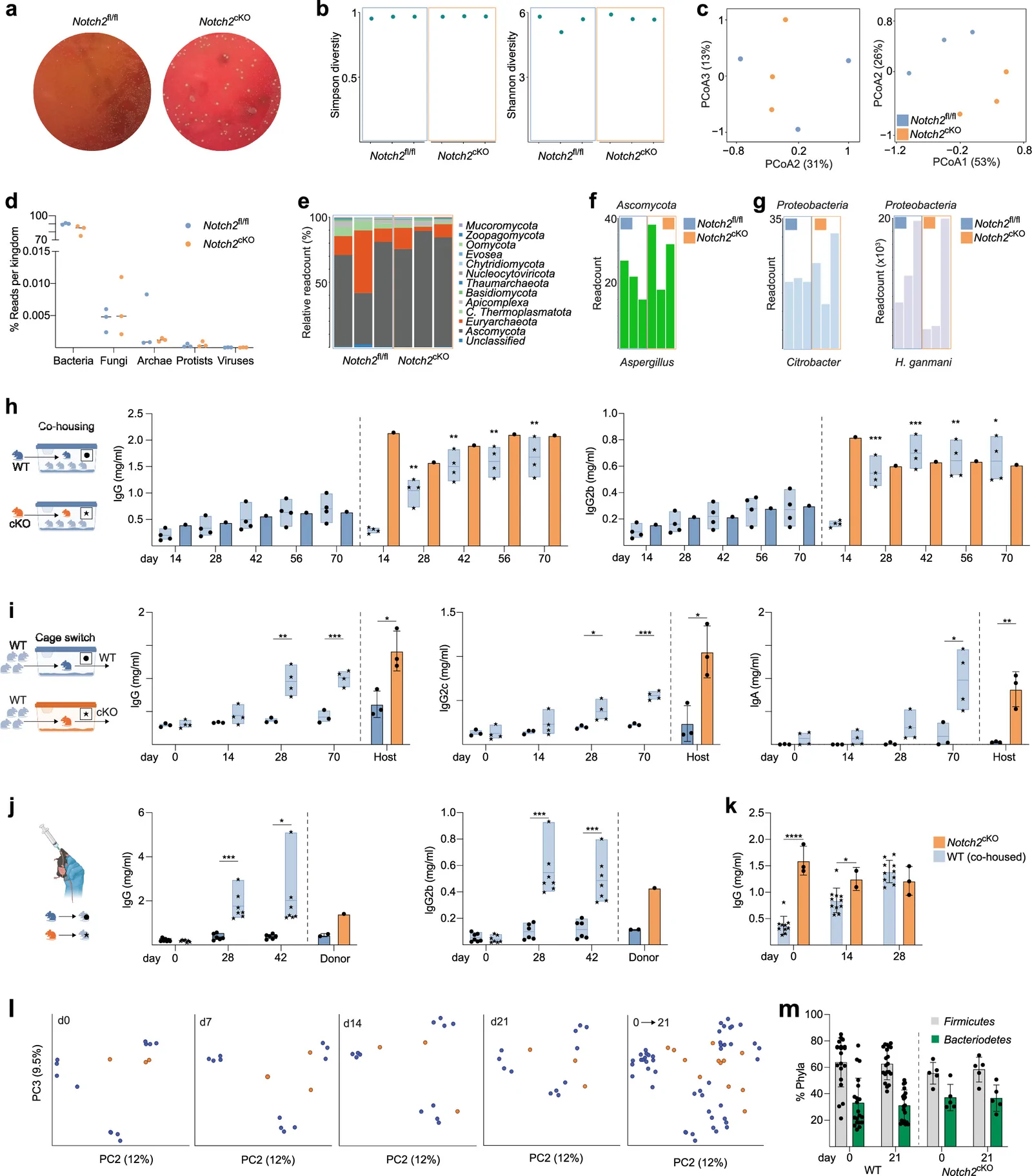
*Notch2*^cKO^ mice have intestinal dysbiosis that can be transferred. Related to Fig. 4. (**a**) Feces were prepared as described in Fig. 4a. Shown are red blood agar plates that were plated with a 1/100 diluted fecal suspension from *Notch2*^fl/fl^ or *Notch2*^cKO^ mice. (**b–g**) Shotgun sequencing was performed as described in Fig. 4b. (*n* = 3 mice). (**b**) α-diversity analysis based on species-level relative abundances. Shannon diversity index (left), and Simpson diversity index (right) for *Notch2*^fl/fl^ (blue square) and *Notch2*^cKO^ (orange square). (**c**) PCoA plot of Jaccard distances at the species level (left), and family level (right) based on shotgun metagenomic profiles of mice from each genotype. Percentages represent the variance explained by each PC. (**d**) Relative abundance of reads per kingdom in fecal samples from *Notch2*^fl/fl^ and *Notch2*^cKO^. (*n* = 3 mice). (**e–g**) Quantification of non-bacterial and bacterial species in *Notch2*^fl/fl^ (blue square) and *Notch2*^cKO^ (orange square) mice. (**e**) Frequency of non-bacterial species. (**f**) Number of *Aspergillus* reads. (**g**) Number of *Citrobacter* (Genus) and *Helicobacter ganmani* reads. (**h**) Co-housing was performed as described in Fig. 4g. Serum immunoglobulin concentrations for each timepoint were determined by ELISA. C57BL/6 co-housed with *Notch2*^fl/fl^ (light blue, circle symbol), control *Notch2*^fl/fl^ (dark blue), C57BL/6* co-housed with *Notch2*^cKO^ (light blue), and control *Notch2*^cKO^ (orange). Statistical analysis was performed between the WT groups. (recipient *n* = 4 mice per experiment, donor: *n* = 1 mouse per experiment, representative data of three independent experiments). (**i**) C57BL/6 mice were placed into *Notch2*^fl/fl^ or *Notch2*^cKO^ bedding for 3 days (cage switch) and into fresh bedding thereafter. Blood was collected biweekly for 70 days and serum immunoglobulin concentrations were analyzed by ELISA in C57BL/6 added to *Notch2*^fl/fl^ cage (light blue, circle symbol), C57BL/6* added to *Notch2*^cKO^ cage (light blue, star symbol), control *Notch2*^fl/fl^ (dark blue), and *Notch2*^cKO^ (orange). (recipient *n* = 3 and 4 mice per experiment, donor: *n* = 3 mice per experiment, representative data of two independent experiments). (**j**) Oral gavage was performed as described in Fig. 4h. Shown are the schematic representation of the experimental design and serum immunoglobulin concentrations at each timepoint, determined by ELISA, in C57BL/6 gavaged with *Notch2*^fl/fl^ fecal material (light blue, circle symbol), C57BL/6* gavaged with *Notch2*^cKO^ fecal material (light blue, star symbol), *Notch2*^fl/fl^ (dark blue), and *Notch2*^cKO^ (orange). (recipient *n* = 6 and 7 mice, donor: *n* = 2 mice and 1 mouse, pooled data from two independent experiments). (**k–m**) The experimental setup for 16S rRNA sequencing of fecal samples from co-housed mice is outlined in Fig. 4i. (**k**) Serum IgG concentrations in WT and *Notch2*^cKO^ mice before and after co-housing were determined by ELISA at the indicated timepoints. (C57BL/6 (timepoints): *n* = 10, 11, and 10 mice, *Notch2*^cKO^ (timepoints): *n* = 3, 2, and 3 mice, representative data from two independent experiments are shown). (**l**) PCoA plots of Jaccard distances at the species level based on the 16S rRNA profiles from mice of each genotype at d0, d7, d14 and d21. Percentages represent the variance explained by each PC (PC2 vs PC3). (C57BL/6: *n* = 12 mice, *Notch2*^cKO^: *n* = 3 mice, representative data from two independent experiments are shown). (**m**) Relative abundance of *Firmicutes* and *Bacteroidetes* in C57BL/6 and *Notch2*^cKO^ mice before and after co-housing at the indicated timepoints. (C57BL/6: *n* = 20 mice, *Notch2*^cKO^: *n* = 5 mice, pooled data from two independent experiments are shown). Data in bar graphs are shown as mean ± SD. Floating bar plots in panel show the median (center line), with bars extending from the minimum to the maximum value. Statistical analysis was performed using an unpaired two-tailed *t*-test. *P > 0.05, **P > 0.01, ***P > 0.001, ****P > 0.0001. Source data

To determine whether the inflammatory phenotype was associated with alterations in the microbiota, we characterized the intestinal microbiome of *Notch2*^cKO^ mice. Fecal pellets yielded higher bacterial colony-forming unit counts compared to WT controls (Fig. 4a), and α-hemolytic species were exclusively detected in cKO mice (Extended Data Fig. 4a). Shotgun metagenomic sequencing showed comparable α-diversity between genotypes, whereas β-diversity analysis revealed distinct clustering of cKO samples and reduced microbial variability relative to WT mice (Fig. 4b and Extended Data Fig. 4b,c). We did not observe significant genotype-dependent differences among fungal, archaeal, protist or viral species (Extended Data Fig. 4d–f, Supplementary Fig. 3a and Supplementary Table 4). Further, both genotypes were dominated by *Bacteroidetes* and *Firmicutes*, whereas *Deferribacteres*, *Chlamydia* and *Tenericutes* were enriched in *Notch2*^cKO^ mice and *Verrucomicrobia* in WT mice (Fig. 4c–e). Health-associated commensals *Lactobacillus johnsonii* and *Akkermansia muciniphila* were enriched in WT mice^43,44^, whereas *Bacteroides caecimuris, Phocaeicola sartorii, Parasutterella MGBC134540* (ref. ^45^), *Helicobacter MGBC103445, Maihella MGBC107932, Mucispirillum MGBC140179* (refs. ^46,47^), *Chlamydia muridarum*^48^ and *Ureaplasma MGBC165308* were exclusive to *Notch2*^cKO^ mice (Fig. 4f). Low read counts of *C. rodentium*^17^ and *Helicobacter ganmani*^49^ were detected in both genotypes (Extended Data Fig. 4g), arguing against a contribution of these pathogens to chronic inflammation. Among the identified species, several emerged as candidate pathobionts based on their reported pathogenicity in patients^50^.

### Intestinal dysbiosis can be transferred to WT mice

To determine whether the inflammatory phenotype was driven by dysbiosis independently of *Notch2* deficiency and to identify candidate microbial drivers, we performed microbiota transfer experiments. To our surprise, WT mice co-housed with *Notch2*^cKO^ mice or exposed to cKO-derived microbiota through cage switching or fecal gavage, without antibiotic conditioning, developed high and persistent IgA, IgG2b and IgG2c titers comparable to those of cKO mice (Fig. 4g,h, Extended Data Fig. 4h–j and Supplementary Fig. 3b–d). These results demonstrated that colonization by the dysbiotic microbiota was independent of the host genotype and sufficient to induce a chronic and systemic immune activation. Analysis of the pathobiome/microbiome composition following co-housing by 16S sequencing, combined with filtering for species detected by shotgun sequencing, allowed us to pinpoint three species that consistently correlated with the observed phenotype: *Parasutterella, Mucispirillum* and *Chlamydia* (Fig. 4f, i). These taxa efficiently colonized WT recipients in 7 days, followed by progressively increasing antibody titers from day 14 onward (Fig. 4j,k and Extended Data Fig. 4k,l). Further, the beneficial commensal *Akkermansia*^43^ was swiftly depleted from the community, whereas *Firmicutes* and *Bacteroidetes* abundance remained largely unchanged (Fig. 4l, Extended Data Fig. 4m and Supplementary Fig. 3e).

Together, these findings implicate a persistent dysbiosis dominated by *Parasutterella*, *Mucispirillum* and *Chlamydia* as a driver of chronic intestinal inflammation and sustained systemic IgA and IgG2c responses.

### Expansion of AXL^+^inf-DC3s exacerbates intestinal inflammation

**Fig. 5:**
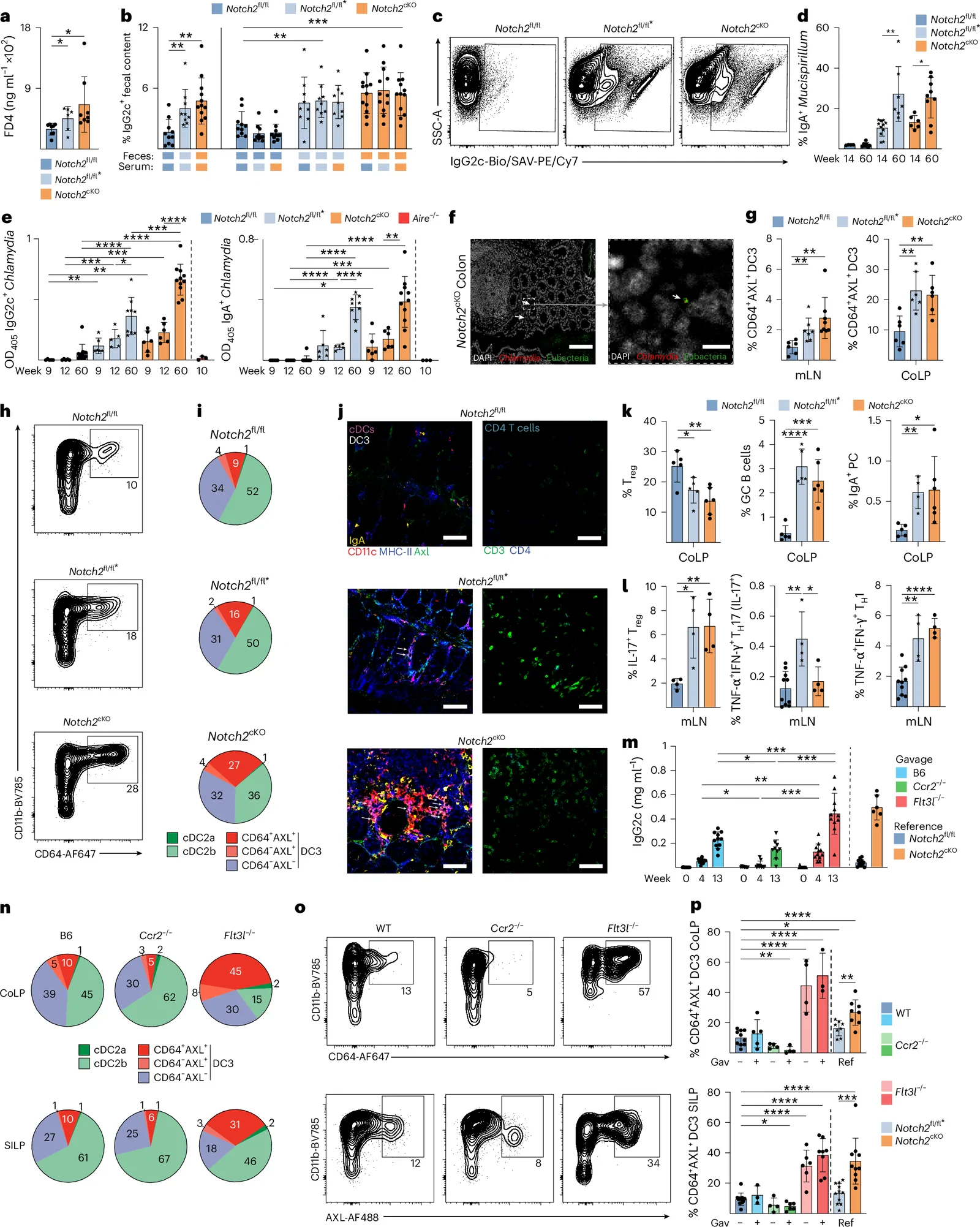
Expansion of AXL^+^inf-DC3s exacerbates intestinal inflammation. **a**, Serum FITC-dextran 4 kDa (FD4) concentration was determined 4 h after oral gavage in 20- to 25-week-old *Notch2*^fl/fl^ (dark blue), *Notch2*^fl/fl^* co-housed with *Notch2*^cKO^ (light blue) and *Notch2*^cKO^ (orange) mice (groups: *n* = 8/6/8 mice, pooled data from two independent experiments). **b**, Frequency of IgG2c-bound fecal content was determined by flow cytometry after staining fecal homogenates with serum. Paired serum and fecal homogenate from the same mouse were used for staining (left). Serum from individual mice was used to stain pooled fecal samples from each genotype (right) (groups: *n* = 10/9/12 mice, pooled data from two independent experiments). **c**,**d**, Frequency of serum IgA-bound *Mucispirillum ASF457*. Representative plots (**c**) and frequencies (**d**) at the indicated ages (14 weeks (groups): *n* = 4/11/6 mice, 60 weeks (groups): *n* = 7/7/9 mice, pooled data and representative plot of two independent experiments). **e**, Serum anti-*Chlamydia muridarum* IgG2c (left) and IgA (right) antibodies from *Notch2*^fl/fl^, co-housed *Notch2*^fl/fl^*, and *Notch2*^cKO^ and autoimmune reference *Aire*^−/−^ (red) mice at the indicated ages were detected by ELISA (9 weeks (groups): *n* = 5/6/6 mice, 12 weeks (groups): *n* = 5/5/6 mice, 60 weeks (groups): *n* = 12/9/11 mice, *Aire*^−/−^: *n* = 3 mice, pooled data from two independent experiments). **f**, Confocal fluorescence microscopy of *Chlamydia* in the colon of *Notch2*^cKO^ mice, stained with DAPI (epithelium, white), and fluorescence in situ hybridization (FISH) probes against Eubacteria (Eub338, green) and *Chlamydia* (Chla232, red). Scale bars: 100 μm (left), 10 μm (right). Arrow indicates the *Chlamydia*/Eubacteria co-staining. Dashed square indicates the magnified area. The images are representative of two independent experiments (each *n* = 3 mice). **g**, Frequency of CD64^+^AXL^+^inf-DC3 among DC2/DC3, pre-gated as in Extended Data Fig. 2g, in mLN (left) and CoLP (right) of 8- to 12-week-old mice was determined by flow cytometry (mLN (groups): *n* = 6/7/8 mice, CoLP (groups): *n* = 6 mice, pooled data from two independent experiments). **h**–**j**, DC3 subsets in CoLP of 25- to 30-week-old mice. **h**, Representative flow cytometry plots showing CD64^+^AXL^+^inf-DC3. **i**, Pie charts showing average DC2/DC3 subset frequencies, pre-gated as in Extended Data Fig. 2g. **j**, Confocal microscopy of consecutive colon sections stained for: (left, section 1) DC3 using CD11c (red), MHC-II (blue), AXL (green), and PC using IgA (yellow); (right, section 2) T cells using CD3 (green) and CD4 (blue). Scale bars: 50 μm. Arrows indicate AXL^+^inf-DC3s (*n* = 5 mice, representative data from two independent experiments). **k**, Frequencies of FoxP3^+^ T_reg_ cells within CD4^+^ T cells (left) pre-gated as in 3j, GC within B cells (middle) pre-gated as in Fig. 3 l, IgA^+^CD138^+^ PCs within live cells (right) in the CoLP of 25- to 30-week-old mice (groups: *n* = 5/5/6 mice, pooled data from two independent experiments). **l**, Frequencies of IL-17 expression within T_reg_ cells (left) pre-gated as in Fig. 3 j, TNF-α^+^IFN-γ^+^ within IL-17^+^T_H_17 (RORγt^+^CD4^+^ T cells) (middle), TNF-α^+^IFN-γ^+^ within T_H_1 (RORγt^−^CD4^+^ T cells) (right) from mLN of 25- to 30-week-old *Notch2*^fl/fl^, co-housed *Notch2*^fl/fl^* and *Notch2*^cKO^ mice following PMA and ionomycin stimulation. (Groups: *n* = 4 or 10/4/4 mice, pooled data from two to four independent experiments). **m**–**p**, C57BL/6 (turquoise), *Ccr2*^−/−^ (green) and *Flt3l*^−/−^ (red) mice (8 weeks old) were gavaged with fecal homogenates (OD_600_ = 0.005) from *Notch2*^cKO^ mice and monitored for up to 13 weeks. **m**, Serum immunoglobulin concentrations were measured by ELISA at the indicated timepoints, including reference (ref) mice (*n* = 11/9/11/11/6 mice, respectively; pooled data from two independent experiments). **n**–**p**, DC populations were analyzed by flow cytometry in the CoLP (top) and SILP (bottom) of ungavaged 30-week-old mice. **n**, DC2 and DC3 subset frequencies, pre-gated as in Extended Data Fig. 2g. **o**, Representative CD64^+^AXL^+^inf-DC3 plots. **p**, Frequency of CD64^+^AXL^+^inf-DC3 among DC2/DC3 without (minus) or after (plus) gavage (Gav).Sample sizes for each group are indicated from left to right (CoLP: *n* = 10/5/4/4/4/3/8/8 mice, SILP: *n* = 11/3/4/6/6/8/11/10 mice, pooled data from two to four independent experiments). Data are shown as mean ± s.d. Statistical analysis was performed using an unpaired two-tailed *t*-test. *P > 0.05, **P > 0.01, ***P > 0.001, ****P > 0.0001. Source data

**Extended Data Fig. 5:**
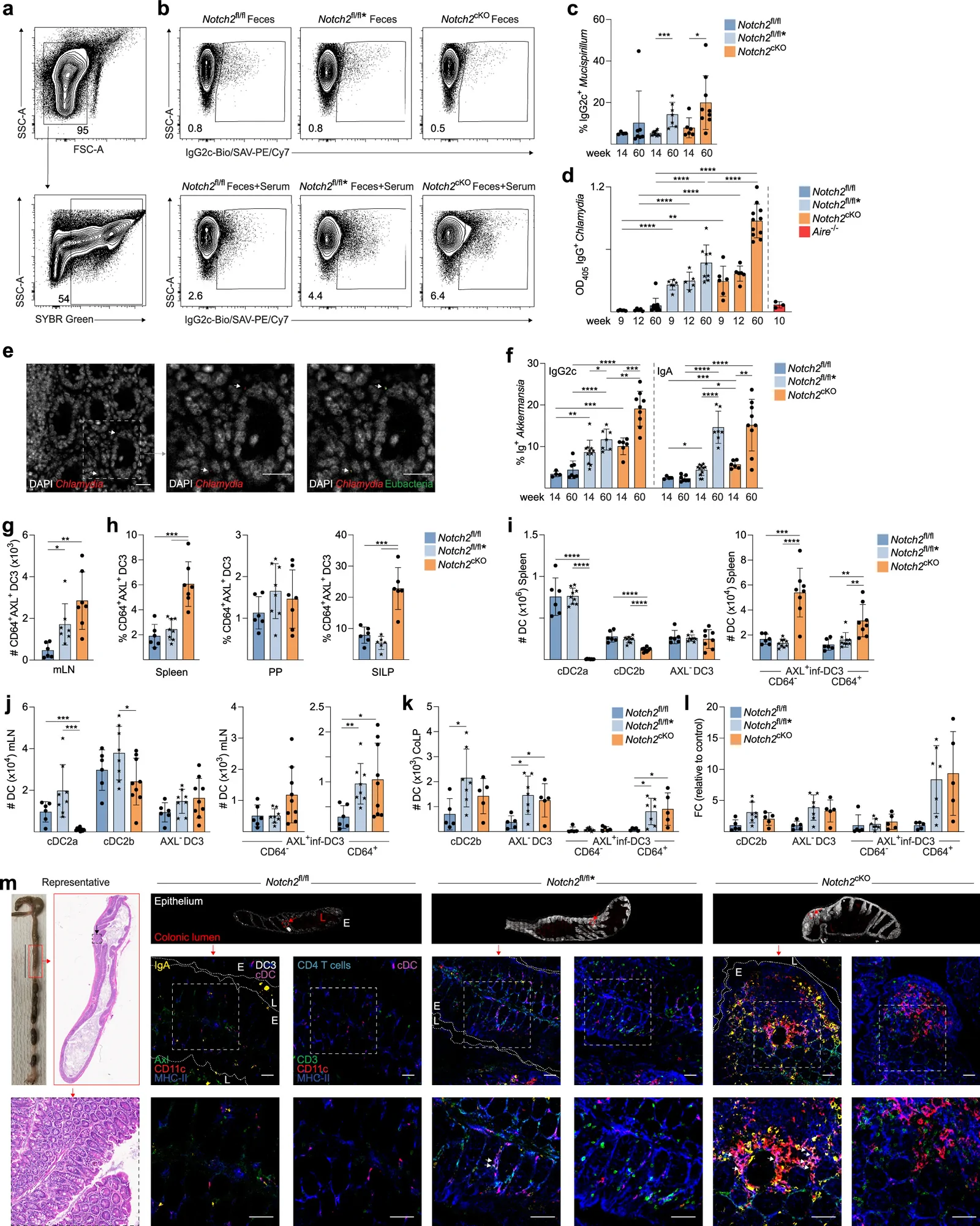
Expansion of AXL^+^inf-DC3 exacerbates intestinal inflammation. Related to Fig. 5. (**a**) Flow cytometry plots showing the gating strategy for fecal bacteria. Size: FSC/SSC (top), and Bacteria: SYBR-Green^+^ (bottom). (**b**) Representative flow cytometry plots showing IgG2c staining of fecal bacteria without (top) or with (bottom) serum staining using serum and fecal homogenate from the same mouse of each indicated genotype. (**c**) Frequency of IgG2c^+^ *Mucispirillum ASF457* following staining with serum from *Notch2*^fl/fl^ (dark blue), *Notch2*^fl/fl^* co-housed with *Notch2*^cKO^ (light blue, star symbol), and *Notch2*^cKO^ (orange) was determined by flow cytometry at the corresponding age in weeks. (14wks (groups): *n* = 4, 11, and 6 mice, 60wks (groups): *n* = 7, 7, and 9 mice, pooled data from two independent experiments). (**d**) Serum anti-*Chlamydia muridarum* specific IgG antibodies from *Notch2*^fl/fl^ (blue), *Notch2*^fl/fl^* co-housed with *Notch2*^cKO^ (light blue), *Notch2*^cKO^ (orange), and autoimmune reference *Aire*^−/−^ (red) mice at the indicated age in weeks were detected by ELISA. (9wks (groups): *n* = 5, 6, and 6 mice, 12wks (groups): *n* = 5, 5, and 6 mice, 60wks (groups): *n* = 12, 9, and 11 mice, *Aire*^−/−^: *n* = 3 mice, pooled data from two independent experiments). (**e**) Confocal fluorescence microscopy of *Chlamydia* in the colon of *Notch2*^cKO^ mice, stained with DAPI (epithelium, white), and fluorescent in situ hybridization (FISH) probes against *Chlamydia* (Chla232, red) and, where indicated, Eubacteria (Eub338, green). Images correspond to Fig. 5f. Scale bars: 20 μm. Arrow indicates the *Chlamydia* and/or Eubacteria staining. Dashed square indicates the magnified area. The images are representative of two independent experiments (each n = 3). (**f**) Frequency of IgA^+^ (left), and IgG2c^+^ (right) *Akkermansia muciniphila* following staining with serum from *Notch2*^fl/fl^ (blue), *Notch2*^fl/fl^* co-housed with *Notch2*^cKO^ (light blue), and *Notch2*^cKO^ (orange) was determined by flow cytometry at the corresponding age in weeks. (14wks (groups): *n* = 4, 11, and 6 mice, 60wks (groups): *n* = 7, 7, and 9 mice, pooled data and representative plot of two independent experiments). (**g-h**) CD64^+^AXL^+^inf-DC3 among DC2/DC3, pre-gated as in Extended Data Fig. 2g, were analyzed by flow cytometry to determine total numbers (**g**) and frequencies (**h**) of the population across the indicated tissues of young *Notch2*^fl/fl^ (dark blue), *Notch2*^fl/fl^* co-housed with *Notch2*^cKO^ (light blue, star symbol), and *Notch2*^cKO^ (orange). (mLN (groups): *n* = 6, 7, and 7 mice, Spleen/PP (groups): *n* = 6, 8, and 7 mice, SILP (groups): *n* = 6 mice, pooled data from two to three independent experiments). (**i–l**) Analysis of DC subsets, pre-gated as in Extended Data Fig. 2g, across indicated tissues of 25-30-week-old *Notch2*^fl/fl^ (dark blue), *Notch2*^fl/fl^* co-housed with *Notch2*^cKO^ (light blue), and *Notch2*^cKO^ (orange). Total cell numbers are indicated for spleen (**i**), mLN (**j**), and CoLP (**k**). (**l**) Shown is the FC relative to control average. (Spleen/mLN (groups): *n* = 6, 9, and 8 mice, CoLP (groups): *n* = 5, 7, and 5 mice, pooled data from two to three independent experiments). (**m**) Confocal fluorescence microscopy of immune cell populations in colon sections from 25-30-week-old *Notch2*^fl/fl^, *Notch2*^fl/fl^*, and *Notch2*^cKO^. A representative macroscopic colon image, H&E-stained sections and confocal images for each genotype are shown for orientation. Consecutive sections from each genotype were stained for either: (left, section 1) DC3 using CD11c (red), MHC-II (blue), AXL (green) and PC using IgA (yellow); (right, section 2) T cells using CD3 (green) and CD4 (blue), and DC using CD11c (red), MHC-II (blue). Images correspond to Fig. 5j. The bottom left image of each quadrant corresponds to the main panel in Fig. 5j and is shown for direct comparison. Scale bar: 2 cm. Red square and dashed black square indicate the magnified area. Scale bars: 50 μm. Arrow indicates the AXL^+^inf-DC3. Dashed square indicates the magnified area. Epithelial tissue is outlined by dashed lines. (*n* = 5 mice, representative data of two independent experiments). Source data

Because dysbiosis is frequently associated with impaired intestinal barrier function^50^, we determined intestinal permeability in *Notch2*^fl/fl^, *Notch2*^cKO^ and *Notch2*^fl/fl^* (co-housed with *Notch2*^cKO^) mice. FITC-dextran intestinal leakage was increased in both *Notch2*^fl/fl^* and *Notch2*^cKO^ mice (Fig. 5a), suggesting enhanced translocation of microbial products. Consistently, serum IgG2c from *Notch2*^fl/fl^* and *Notch2*^cKO^ but not WT mice showed greater reactivity against autologous and heterologous fecal suspensions (Fig. 5b and Extended Data Fig. 5a,b). IgA and IgG2c were reactive against both *Mucispirillum* (ASF457) and *Chlamydia muridarum* (Fig. 5c–e and Extended Data Fig. 5c,d). Consistent with persistent antigen exposure, anti-*Chlamydia* IgG2c titers increased over time. We confirmed the presence of *Chlamydia* within the CoLP of cKO mice (Fig. 5f and Extended Data Fig. 5e). Nevertheless, we also detected anti-*Akkermansia* antibodies in *Notch2*^fl/fl^* and *Notch2*^cKO^ mice (Extended Data Fig. 5f), suggesting a broad exposure to microbiota-derived antigens, compatible with increased permeability.

**Extended Data Fig. 6:**
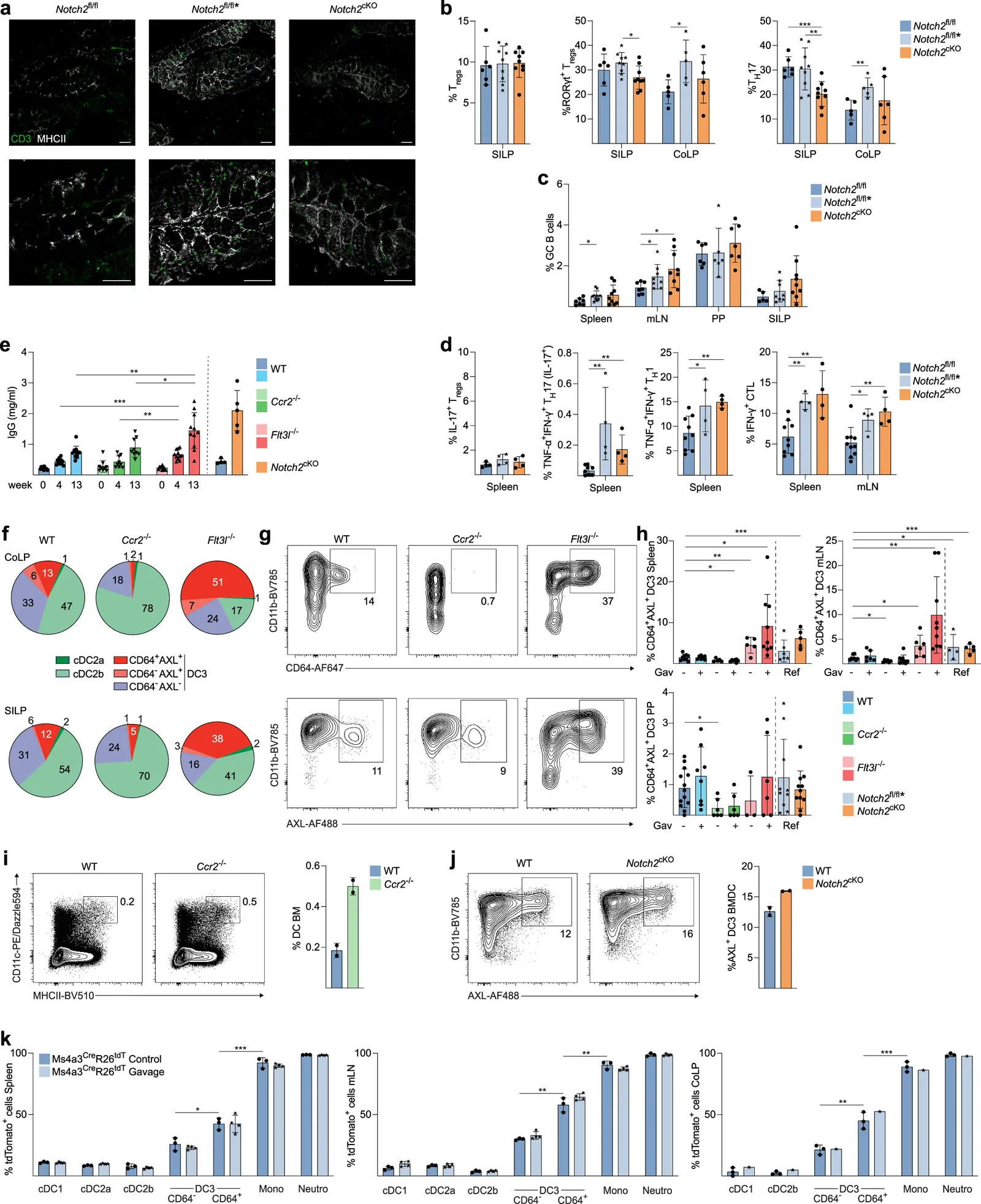
Expansion of AXL^+^inf-DC3 exacerbates intestinal inflammation. Related to Fig. 5. (**a**) Confocal fluorescence microscopy of immune cell populations in colon section from 25-30-week-old *Notch2*^fl/fl^, *Notch2*^fl/fl^*, and *Notch2*^cKO^. Sections from each genotype stained for CD3 (green) and MHC-II (white). Scale bars: 100 μm. (*n* = 5 mice, representative data of two independent experiments). (**b**) Frequency of FoxP3^+^ T_reg_ within CD4^+^ T cells (left) pre-gated as in 3j, RORγt within T_reg_ (middle), and RORγt within CD4^+^ T cells (right) was determined by flow cytometry. (SILP (groups): *n* = 6/9/9 mice, CoLP (groups): *n* = 5/5/6 mice, pooled data from two to three independent experiments). (**c**) Frequency of GC within B cells, pre-gated as in 3 l, in the indicated tissues of 25-30-week-old *Notch2*^fl/fl^ (dark blue), *Notch2*^fl/fl^* co-housed with *Notch2*^cKO^ (light blue), and *Notch2*^cKO^ (orange) was determined by flow cytometry. (Spleen (groups): *n* = 7/10/9 mice, mLN (groups): *n* = 7/8/9 mice, PP (groups): *n* = 6/6/7 mice, SILP (groups): *n* = 5/8/9 mice, pooled data from two to three independent experiments). (**d**) Frequency of IL-17 expression within T_reg_ (left) pre-gated as in 3j, TNF-α^+^IFN-γ^+^ within IL-17^+^T_H_17 (RORγt^+^CD4^+^ T cells) (middle left), TNF-α^+^IFN-γ^+^ within T_H_1 (RORγt^−^CD4^+^ T cells) (middle right), IFN-γ^+^ within cytotoxic T lymphocytes (CTL, CD8^+^ T cells) (right) from the indicated tissues of 25-30-week-old *Notch2*^fl/fl^ (blue), *Notch2*^fl/fl^* co-housed with *Notch2*^cKO^ (light blue), and *Notch2*^cKO^ (orange), determined by flow cytometry after PMA and ionomycin stimulation. (T_reg_ (groups): *n* = 4 mice, T_H_17/T_H_1/CTL (groups): 9 or 10/4/4 mice, pooled data from two to three independent experiments). (**e–h**) Experimental setup is described in Fig. 5m-p and includes C57BL/6 (turquoise), *Ccr2*^−/−^ (green), *Flt3l*^−/−^ (red), *Notch2*^fl/fl^ (dark blue), *Notch2*^fl/fl^* co-housed with *Notch2*^cKO^ (light blue), and *Notch2*^cKO^ (orange) mice. (**e**) Serum immunoglobulin concentrations for the described mice were determined by ELISA for each timepoint. (Sample sizes for each group are indicated from left to right: *n* = 11/9/11/4/5 mice, pooled data from two independent experiments). (**f–h**) DC populations were analyzed by flow cytometry at the age of 30 weeks in the CoLP (top) and SILP (bottom) for each genotype after gavage. (**f**) Pie charts showing DC2 and DC3 subset frequencies pre-gated as in Extended Data Fig. 2g for each genotype. (**g**) Representative flow cytometry plots of CD64^+^AXL^+^inf-DC3. (**h**) Frequency of CD64^+^AXL^+^inf-DC3 in spleen (top left), mLN (top right middle), and PP (bottom) without (minus) or after (plus) gavage (Gav). (Sample sizes for each group are indicated from left to right. Spleen: *n* = 9/7/5/7/5/9/6/5 mice, mLN: *n* = 8/6/6/9/6/9/4/5 mice, PP: *n* = 13/8/6/6/3/6/10/11 mice, pooled data from two to four independent experiments). (**i**) Flow cytometry plots and frequency of CD11c^+^MHC-II^+^ DC within live cells from bone marrow (BM) of C57BL/6 (blue) or *Ccr2*^−/−^ (green). (*n* = 2 mice, one representative experiment). (**j**) BM cells from C57BL/6 and *Notch2*^cKO^ were cultured in the presence of FLT3L for 7 days. Shown are representative flow cytometry plots (left) and quantification (right) of AXL^+^DC3, pre-gated as CD11c^+^MHC-II^+^CD26^+^XCR1^−^SiglecH^−^. (*n* = 2, representative of two independent experiments). (**k**) *Ms4a3*^Cre^*Rosa26*^tdTomato^ mice (15-week-old) were gavaged with fecal homogenates (OD_600_ = 20) derived from *Notch2*^cKO^ mice. The frequency of reporter-positive cells within the indicated cell types in the spleen (left), mLN (middle), and CoLP (right) of control (dark blue) and gavaged mice (light blue) was analyzed by flow cytometry after 15 weeks. Frequency of tdTomato-labeling was assessed within cDC1 (XCR1^+^), cDC2a (ESAM^+^), cDC2b (CX3CR1^+^CD16/32^−^), CD64±^−^DC3 (CX3CR1^+^CD16/32^+^), monocytes (CX3CR1^+^Ly6C^+^) and neutrophils (Ly6G^+^Ly6C^+^). (Spleen/mLN: *n* = 3 and 4 mice, CoLP: *n* = 3 mice and 1 mouse, pooled data from two independent experiments). Data are shown as mean ± SD. Statistical analysis was performed using an unpaired two-tailed *t*-test. *P > 0.05, **P > 0.01, ***P > 0.001, ****P > 0.0001. Source data

We next wondered whether the expansion of AXL^+^inf-DC3s was also linked to the dysbiotic phenotype, given their localization and inflammatory profile. The induction of dysbiosis by co-housing also promoted AXL^+^inf-DC3s accumulation in *Notch2*^fl/fl^* mice, with frequencies increasing in mLN and nearly doubling in the colon (Fig. 5g and Extended Data Fig. 5g,h). Colonic AXL^+^inf-DC3s remained expanded for up to 30 weeks (Fig. 5h,i and Extended Data Fig. 5i–l), which is conceivable with a chronic exposure to microbial products. Dysbiosis also promoted epithelial MHC-II upregulation and CD4^+^ T cell accumulation, consistent with local inflammation (Extended Data Fig. 5m and Extended Data Fig. 6a). In *Notch2*^cKO^ mice, we identified tertiary lymphoid structures containing IgA-producing B cells, CD4^+^ T cells and AXL^+^inf-DC3s (Fig. 5j, Extended Data Fig. 5m). Colonic T_reg_ cells were reduced, whereas GC B cells and IgA-secreting PC increased in both *Notch2*^cKO^ and *Notch2*^fl/fl^* mice (Fig. 5k and Extended Data Fig. 6b,c). Proinflammatory T cell subsets, including IL-17^+^FOXP3^+^ex-T_reg_, TNF-α^+^IFN-γ^+^T_H_1 and T_H_17 cells, accumulated upon dysbiosis. In contrast, T_H_17 cells failed to expand in *Notch2*^cKO^ mice^16^, suggesting that the genetic defect may have contributed to the initial establishment of these pathobionts within our mouse colony (Fig. 5l and Extended Data Fig. 6b,d).

However, to directly assess the contribution of AXL^+^inf-DC3s to the inflammatory response and the overall phenotype, we challenged *Ccr2*^−/−^ and *Flt3l*^−/−^ mice with highly diluted *Notch2*^cKO^ fecal content (Fig. 5m–p and Extended Data Fig. 6e–h). These strains displayed reduced and increased frequencies of AXL^+^inf-DC3s, respectively, already at steady state. *Ccr2*^−/−^ mice showed a delayed and attenuated antibody response, consistent with reduced AXL^+^inf-DC3 frequencies, which may depend on CCR2 for their bone marrow (BM) egress^51^ (Extended Data Figs. 2d and 6i). In contrast, *Flt3l*^−/−^ mice exhibited accelerated and heightened antibody responses, in line with increased AXL^+^inf-DC3 abundance. Notably, despite their partial in vivo independence from FLT3L^52^, compatible with low expression of *Flt3* (Extended Data Fig. 2d), AXL^+^inf-DC3s could still develop in FLT3L-supplemented BM cultures (Extended Data Fig. 6j), suggesting that they may possess a selective advantage over other DC subsets under steady-state and dysbiotic conditions, where they reached frequencies around 40% in the CoLP and SILP (Fig. 5m–p and Extended Data Fig. 6e–h). Collectively, these results directly link AXL^+^inf-DC3 abundance with the inflammatory phenotype. tdTomato labeling of AXL^+^inf-DC3s remained unchanged in *Ms4a3*^Cre^*Rosa26*^tdTomato^^29,31^ mice before and after establishment of dysbiosis (Extended Data Fig. 6k), arguing against their expansion through increased monocytic contribution. However, as about 30% of DC3s (refs. ^29,31^) and 50% of AXL^+^inf-DC3 were tdTomato^+^, their lineage of origin remains to be determined.

Together, these data suggest that AXL^+^inf-DC3s reinforce the inflammatory circuit linking dysbiosis, barrier dysfunction and microbial antigen exposure, promoting sustained antibody responses against microbiota-derived antigens.

### Colonization by three pathobionts is sufficient to drive autoimmune features

**Fig. 6:**
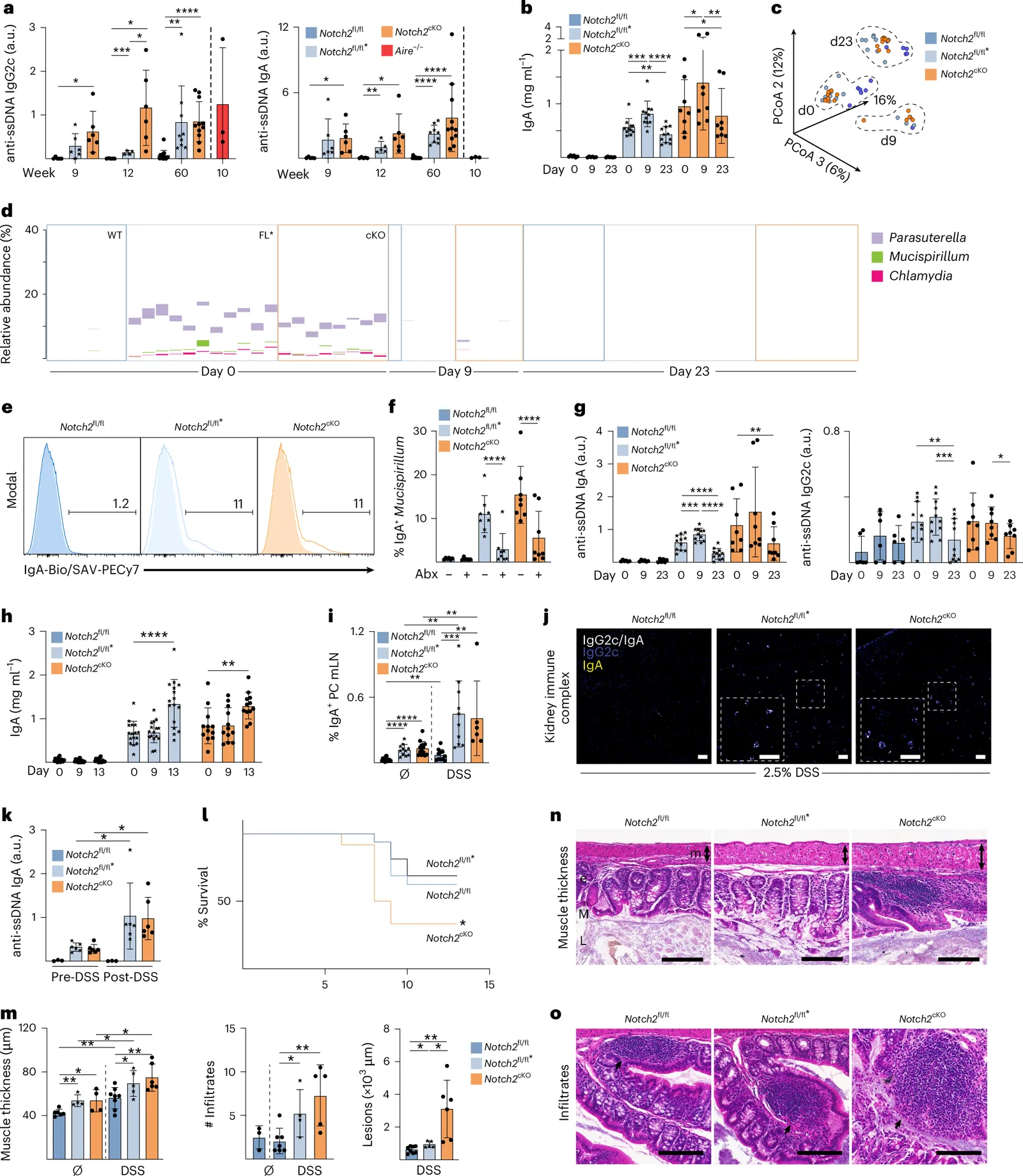
Colonization by three pathobionts is sufficient to drive autoimmune features. **a**, Serum anti-ssDNA specific IgG2c (left) and IgA (right) antibody concentration in *Notch2*^fl/fl^ (dark blue), *Notch2*^fl/fl^* co-housed with *Notch2*^cKO^ (light blue), *Notch2*^cKO^ (orange) and autoimmune reference *Aire*^−/−^ (red) mice at the indicated age in weeks was measured by ELISA. Data are presented as arbitrary units (a.u.) (9 weeks (groups): *n* = 5/6/6 mice, 12 weeks (groups): *n* = 5/4 or 5/6 mice, 60 weeks (groups): *n* = 14/9/11 mice, *Aire*^−/−^: *n* = 3 mice, pooled data from two independent experiments). **b**–**g**, Antibiotic treatment was administered via drinking water of 15-week-old mice for 9 days, followed by regular water for 2 weeks (until day 23). Serum and fecal samples were collected at days 0, 9 and 23. **b**, Serum immunoglobulin concentrations in 15-week-old mice throughout the antibiotic treatment study (groups: *n* = 6/11/8 mice, pooled data from two independent experiments). **c**,**d**, 16S rRNA sequencing was performed on fecal samples collected during the antibiotic treatment. **c**, 3D-PCoA of species-level Jaccard distances from 16S rRNA profiles at each timepoint. Percentages represent the variance explained by each PC. **d**, Relative abundance of bacteria in feces of C57BL/6, co-housed *Notch2*^fl/fl^*, and *Notch2*^cKO^ (groups: *n* = 6/11/8 mice, pooled data from two independent experiments). For day 9, shown are mice with detectable reads. **e**–**g**, Analysis before and after antibiotic (Abx) treatment as described in (**b**). **e**,**f**, Frequency of serum IgA-bound *Mucispirillum ASF457* before and after antibiotic treatment. **e**, Representative flow cytometry histograms indicate IgA binding before (line only) or after antibiotic (colored shape) treatment. **f**, Frequencies of IgA^+^ *Mucispirillum ASF457* bound by serum IgA from 15-week-old mice before (minus) and after (plus) antibiotic treatment (groups ± Abx: *n* = 6/8/8 mice, pooled data from two independent experiments). **g**, Serum anti-ssDNA specific IgA (left) and IgG2c (right) antibody concentrations throughout the antibiotic treatment study. Data are presented as arbitrary units (a.u.) (groups: *n* = 6/11/8 mice, pooled data from two independent experiments). **h**–**o**, Colitis was induced by administering 2.5% DSS in drinking water for 7 days, followed by regular water for 6 days in 25- to 30-week-old *Notch2*^fl/fl^, co-housed *Notch2*^fl/fl^* and *Notch2*^cKO^ mice. **h**, Serum immunoglobulin concentrations at the indicated timepoints during the DSS-induced colitis study (groups: *n* = 16/15/12 mice, pooled data from three independent experiments). **i**, Frequency of IgA^+^CD138^+^ PC within live cells in the mLN without (Ø) or with DSS treatment (no DSS (groups): *n* = 14/11/13 mice, DSS (groups): *n* = 11/10/6 mice, pooled data from three independent experiments). **j**, Confocal microscopy of kidney immune complex deposition after DSS treatment, stained for IgG2c (blue) and IgA (yellow). Scale bars: 150 μm. Dashed square indicates the magnified area. The images are representative of two independent experiments (each *n* = 3 mice). **k**, Serum anti-ssDNA specific IgA antibody concentrations before and after DSS treatment. Data are presented as arbitrary units (a.u.) (groups ± DSS: *n* = 3/6/6 mice, pooled data from three independent experiments). **l**, Survival frequency throughout the 13-day study period (groups: *n* = 16/16/12 mice, pooled data from three independent experiments). **m**–**o**, H&E-stained colons from each genotype were assessed. **m**, Quantification of muscle thickness without (Ø) or with DSS treatment (left), number of infiltrates (middle), and lesion length (right). Sample sizes for each group are indicated from left to right. Muscle thickness: *n* = 6/4/4/8/5/6 mice, number infiltrates: *n* = 3/7/4/5 mice, lesion length: *n* = 8/5/6 mice, pooled data from three independent experiments. **n**–**o**, Representative H&E-stained colons showing muscle thickness (**n**) and tissue infiltrates (**o**). m, muscle; e, epithelium; M, mucus; L, lumen. **n**, Arrows indicate muscle thickness. **o**, Arrows indicate tissue infiltrates. **n**–**o**, Scale bars: 125 μm. The images are representative of two independent experiments (each *n* = 3 mice). Data are shown as mean ± s.d. Statistical analysis was performed using an unpaired two-tailed *t*-test for comparisons between independent groups or a paired two-tailed *t*-test for repeated measures involving the same mice across multiple timepoints. *P > 0.05, **P > 0.01, ***P > 0.001, ****P > 0.0001. Source data

**Extended Data Fig. 7:**
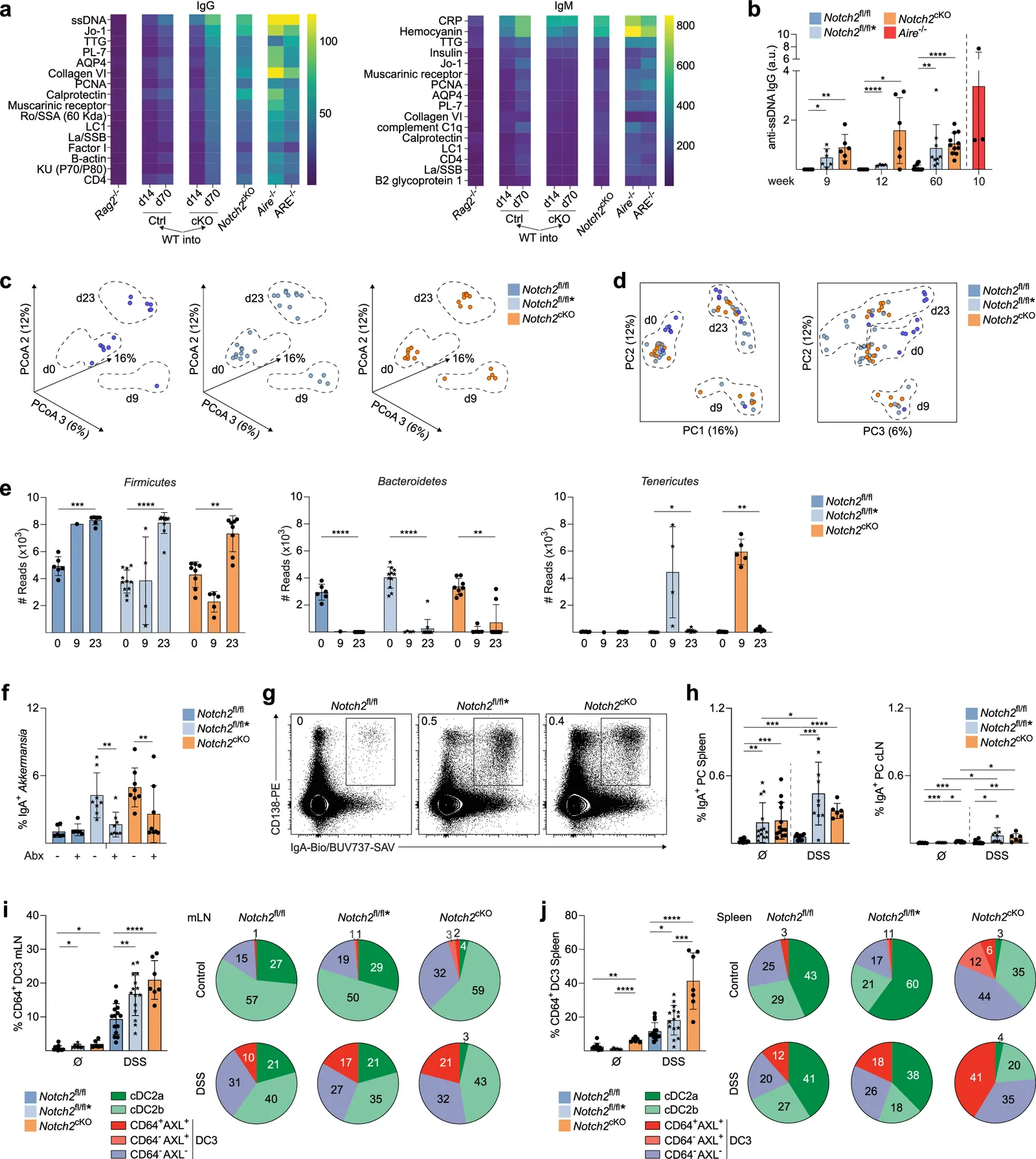
Colonization by three pathobionts is sufficient to drive autoimmune features. Related to Fig. 6. (**a**) Autoreactive serum antibodies were screened using an autoantigen array for IgG (left) and IgM (right). Serum samples were obtained from *Rag2*^−/−^, C57BL/6 co-housed with *Notch2*^fl/fl^ after 14 or 70 days, C57BL/6 co-housed with *Notch2*^cKO^ after 14 or 70 days, 50-week-old *Notch2*^cKO^, the autoimmune references *Aire*^−/−^ and *Ifng* 3′UTR deleted Adenylate-Uridylate Rich element (ARE-del^−/−^). Heatmaps show the top 16 autoantigen hits. Each condition represents pooled serum from 3-4 mice. Scale indicates a relative signal to noise ratio (SNR). (**b**) Serum anti-ssDNA specific IgG antibody concentration in *Notch2*^fl/fl^ (blue), *Notch2*^fl/fl^* co-housed with *Notch2*^cKO^ (light blue), *Notch2*^cKO^ (orange), and autoimmune reference *Aire*^−/−^ (red) mice at the indicated age in weeks was measured by ELISA. Data are presented as arbitrary units (a.u.). (9wks (groups): *n* = 5/6/6 mice, 12wks (groups): *n* = 5/4/6 mice, 60wks (groups): *n* = 14/9/11 mice, *Aire*^−/−^: *n* = 3, pooled data from two independent experiments). (**c-e**) Antibiotic treatment was performed as described in Fig. 6b on 15-week-old *Notch2*^fl/fl^ (dark blue), *Notch2*^fl/fl^* co-housed with *Notch2*^cKO^ (light blue), and *Notch2*^cKO^ (orange). 16S rRNA sequencing was performed on the fecal material collected for 23 days during the antibiotic treatment study. (**c**) 3D-PCoA plot of Jaccard distances at the species level based on the 16S rRNA profiles from mice of each genotype at d0, d9 and d23. Each plot represents a genotype. Percentages represent the variance explained by each PC. (**d**) 2D-PCoA plots of Jaccard distances at the species level based on the 16S rRNA profiles from mice of each genotype at d0, d9 and d23. PCoA1 vs PCoA2 (left), and PCoA2 vs PCoA3 (right). Percentages represent the variance explained by each PC. (**e**) Number of reads within the phyla *Firmicutes* (left), *Bacteroidetes* (middle), and *Tenericutes* (right) for the indicated genotypes and timepoints. (Groups: *n* = 6/11/8 mice, pooled data from two independent experiments). (**f**) Antibiotic treatment was performed as described in Fig. 6b on 15-week-old *Notch2*^fl/fl^ (dark blue), *Notch2*^fl/fl^* co-housed with *Notch2*^cKO^ (light blue), and *Notch2*^cKO^ (orange). Frequency of IgA^+^ *Akkermansia muciniphila* bound by serum immunoglobulins was determined by flow cytometry of serum-stained bacteria before (minus) and after (plus) antibiotic treatment. (Groups ± Abx: *n* = 6/8/8 mice, pooled data from two independent experiments) (**g–j**) The DSS-colitis study was performed as described in Fig. 6h on 25–30-week-old *Notch2*^fl/fl^ (blue), *Notch2*^fl/fl^* co-housed with *Notch2*^cKO^ (light blue), and *Notch2*^cKO^ (orange) mice. (**g-h**) IgA^+^ PC were analyzed by flow cytometry in mice, without (Ø) or with DSS treatment. (**g**) Representative flow cytometry plots of IgA^+^CD138^+^ PC within splenic live cells of DSS treated mice. (**h**) Frequency of CD138^+^IgA^+^ PC within live cells in the spleen (left) and cLN (right) was determined by flow cytometry in mice of the indicated genotypes, without (Ø) or with DSS treatment. (Sample sizes for each group are indicated from left to right. Spleen: *n* = 13/13/13/11/10/6 mice, cLN: *n* = 7/8/8/11/10/6 mice, pooled data from two to three independent experiments). (**i-j**) DC populations were analyzed by flow cytometry in mice, without (Ø) or with DSS treatment. Frequency of CD64^+^AXL^+^inf-DC3 (left), and pie charts showing DC2/DC3 subset frequencies (right) in the mLN (**i**) and spleen (**j**) of mice across genotypes and conditions. Cells were pre-gated as in Extended Data Fig. 2g. (Sample sizes for each group are indicated from left to right. mLN: *n* = 12/8/6/14/15/7 mice, Spleen: *n* = 9/6/6/14/15/7 mice, pooled data from three independent experiments). Data are shown as mean ± SD. Statistical analysis was performed using an unpaired two-tailed *t*-test for comparisons between independent groups, or a paired two-tailed *t*-test for repeated measures involving the same mice across multiple timepoints. *P > 0.05, **P > 0.01, ***P > 0.001, ****P > 0.0001. Source data

Despite extensive evidence linking dysbiosis to autoimmunity, conflicting associations with individual bacterial strains and other factors have complicated efforts to define individual contributions to disease pathogenesis^53,54^. We observed that specific colonization with *Mucispirillum*, *Chlamydia* and *Parasutterella* was sufficient to establish persistent dysbiosis, expansion of AXL^+^inf-DC3 and an increase in systemic antibody responses and lead to autoantibody production that shared the same isotypes (Fig. 6a, Extended Data Fig. 7a,b and Supplementary Fig. 4a). To prove the causality, we treated WT, *Notch2*^fl/fl^* and *Notch2*^cKO^ mice with broad-spectrum antibiotics for 9 days and monitored them for 3 weeks. Total and specific IgA transiently increased at day 9 before declining by day 23 (Fig. 6b), reflecting reduction in *Mucispirillum*, *Chlamydia* and *Parasutterella* reads by 16S sequencing (Fig. 6c–f, Extended Data Fig. 7c–f and Supplementary Fig. 4b). Surprisingly, anti-ssDNA IgA and IgG2c autoantibodies showed similar kinetics (Fig. 6g), linking antimicrobial and autoreactive antibody responses.

To our surprise, barrier disruption by dextran sulfate sodium (DSS) treatment did not result in increased total IgA in WT eubiotic mice but exacerbated disease and phenotype in *Notch2*^fl/fl^* and *Notch2*^cKO^ mice, suggesting that transient barrier leakage is not sufficient to promote increased antibody titers and autoimmune features but specific pathobiont colonization is required (Fig. 6h,i, Extended Data Fig. 7g,h and Supplementary Fig. 4c). Immune complex deposition and autoantibody titers notably increased in dysbiotic groups following DSS treatment (Fig. 6j,k). *Notch2*^fl/fl^* displayed a comparable phenotype to *Notch2*^cKO^ following DSS treatment, as determined by IgA^+^ PC expansion, autoantibody titers and immune complex deposition, compatible with a memory response. However, these mice showed an intermediate phenotype in terms of tissue pathology and lethality, consistent with expansion of T_reg_ cells (Supplementary Fig. 4d,e) and inflammatory TNF-α^+^IFN-γ^+^T_H_17 (Supplementary Fig. 4f) cells, that failed to develop in *Notch2*^cKO^ mice and may contain bacterial exposure (Fig. 6l–o and Supplementary Fig. 4g,h). DSS treatment expanded AXL^+^inf-DC3 in all mice, further supporting their function in regulating microbiome driven immunity (Extended Data Fig. 7i,j and Supplementary Fig. 4i).

Collectively, these findings support our hypothesis that colonization by *Mucispirillum*, *Chlamydia* and *Parasutterella* promotes dysbiosis that results in the development of chronic intestinal inflammation and the occurrence of autoimmune symptoms, exacerbating disease severity following acute colitis.

### AXL^+^inf-DC3s respond to intestinal dysbiosis

**Fig. 7:**
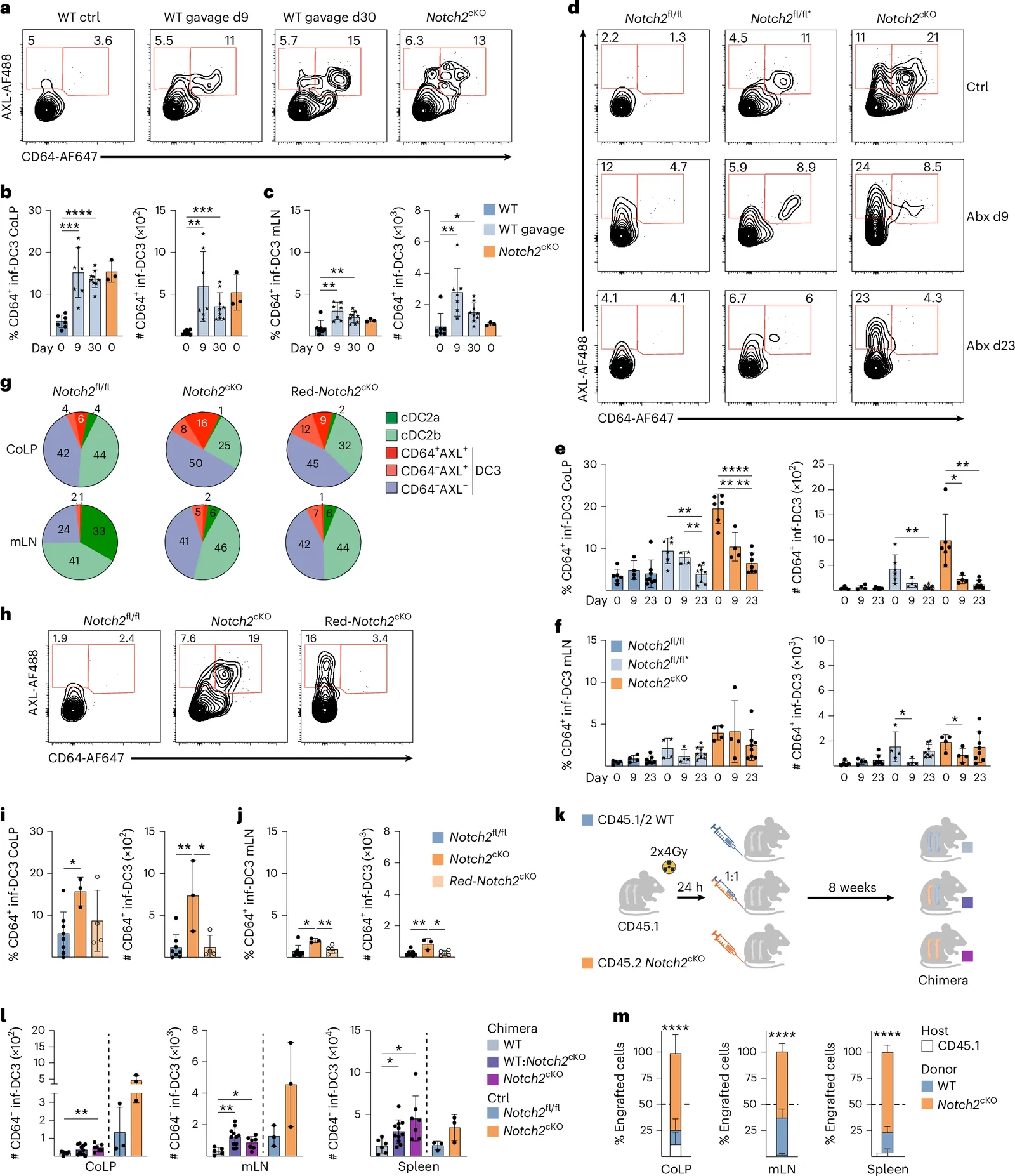
AXL^+^inf-DC3s respond to intestinal dysbiosis. **a**–**c**, C57BL/6 mice (7–10 weeks) were gavaged with fecal homogenates (OD_600_ = 20) derived from *Notch2*^cKO^ mice. DC populations in CoLP and mLN were analyzed by flow cytometry at the indicated timepoints in C57BL/6 (dark blue), C57BL/6 gavaged with *Notch2*^cKO^ fecal material (light blue), and *Notch2*^cKO^ (orange). **a**, Representative flow cytometry plots showing CD64^+^AXL^+^inf-DC3 (red gate) and CD64^−^AXL^+^inf-DC3s (light red gate) among DC2/DC3s, pre-gated as in Extended Data Fig. 2g, in the CoLP of each genotype and condition. **b**,**c**, Frequencies (left) and total cell numbers (right) of CD64^+^AXL^+^inf-DC3 in the CoLP (**b**) and mLN (**c**) are shown for each genotype and condition. Sample sizes for each group are indicated from left to right (CoLP/mLN: *n* = 7, 7, 9, and 3 mice, pooled data from two independent experiments). **d**–**f**, Antibiotics were administered via drinking water of 15- to 20-week-old *Notch2*^fl/fl^ (dark blue), *Notch2*^fl/fl^* co-housed with *Notch2*^cKO^ (light blue), and *Notch2*^cKO^ (orange) for 9 days, followed by regular water for 2 weeks (until day 23). DC populations in CoLP and mLN were analyzed by flow cytometry at each timepoint. **d**, Representative flow cytometry plots showing CD64^+^AXL^+^inf-DC3 (red gate) and CD64^−^AXL^+^inf-DC3 (light red gate) among DC2/DC3, pre-gated as in Extended Data Fig. 2g, in the CoLP of each genotype and condition. **e**,**f**, Frequencies (left) and total cell numbers (right) of CD64^+^AXL^+^inf-DC3 are shown for CoLP (**e**) and mLN (**f**) of each genotype and condition (CoLP (timepoint): *n* = 6, 4, and 7 or 8 mice, mLN (timepoint): *n* = 4 or 5, 4, and 7 or 8 mice, pooled data from two independent experiments). **g**–**j**, Analysis of DC subsets within CoLP (top) and mLN (bottom) of 8- to 12-week-old *Notch2*^fl/fl^ (dark blue), *Notch2*^cKO^ (orange) and rederived *Notch2*^cKO^ (light orange) mice. **g**, Pie charts showing average DC2 and DC3 subset frequencies, pre-gated as in Extended Data Fig. 2g. **h**, Representative flow cytometry plots showing CD64^+^AXL^+^inf-DC3s (red gate) and CD64^−^AXL^+^inf-DC3s (light red gate) in the CoLP of each genotype and condition. **i**,**j**, Frequencies (left) and total cell numbers (right) of CD64^+^AXL^+^inf-DC3s are shown for CoLP (**i**) and mLN (**j**) of each genotype and condition (groups: *n* = 8, 3, and 4 mice, pooled data from two to three independent experiments). **k**–**m**, BM chimeras were generated by transferring total BM from CD45.1/2 (WT), CD45.2 (*Notch2*^cKO^) or a 1:1 mixture of both (WT: *Notch2*^cKO^) into sublethally irradiated CD45.1 recipient mice, followed by reconstitution for 8 weeks. **k**, Schematic representation of the BM chimera setup. **l**,**m**, Quantification (**l**) of CD64^−^AXL^+^inf-DC3 and frequency of donor-derived cells (**m**) within this population, pre-gated as in Extended Data Fig. 2g, in the indicated tissues. Sample sizes for each group are indicated from left to right (*n* = 6, 11, 7, 3, and 3 mice, pooled data from two independent experiments). Data are shown as mean ± s.d. Statistical analysis was performed using an unpaired two-tailed *t*-test. *P > 0.05, **P > 0.01, ***P > 0.001, ****P > 0.0001. Source data

**Extended Data Fig. 8:**
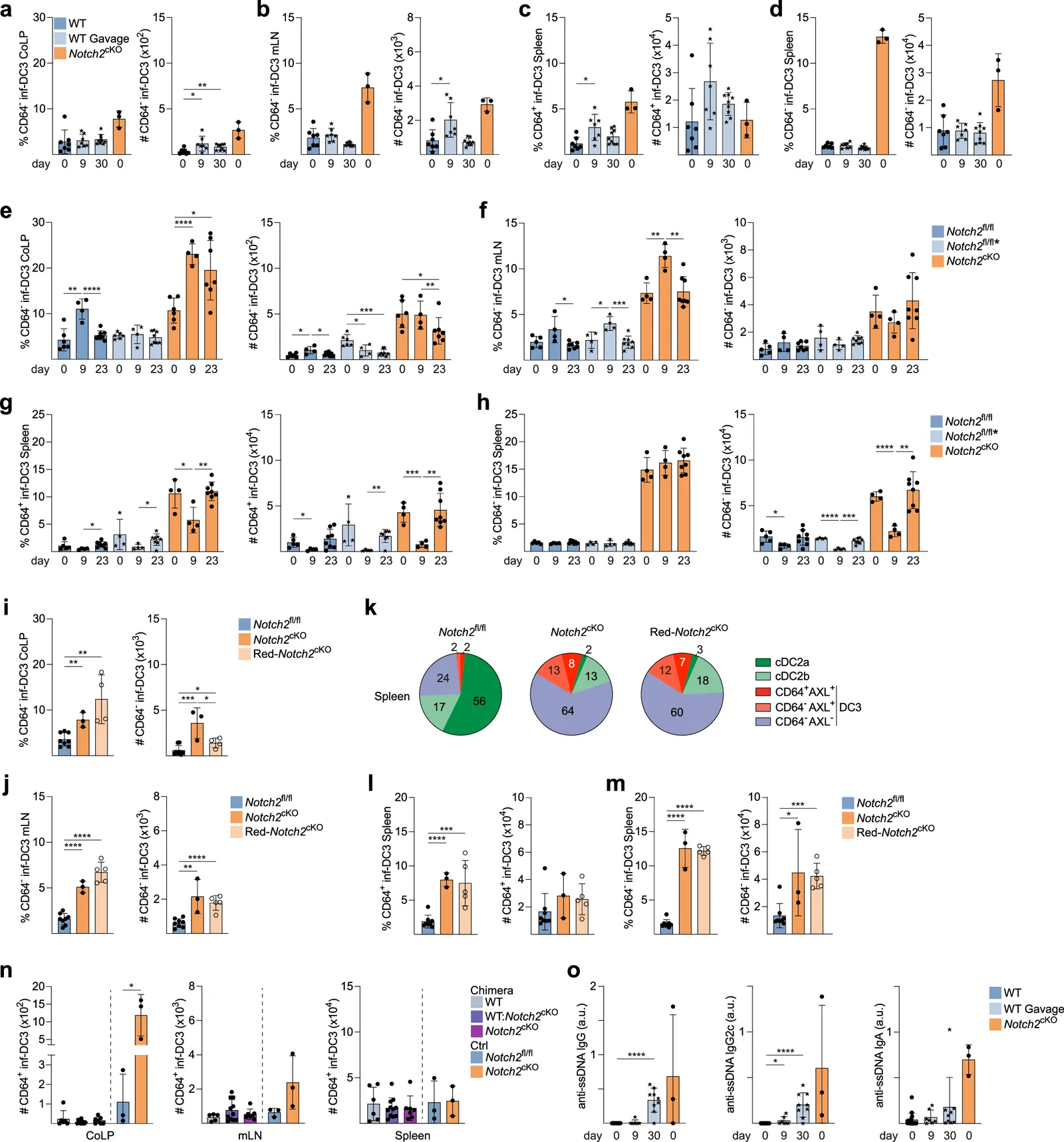
AXL^+^inf-DC3 respond to intestinal dysbiosis. Related to Fig. 7. (**a–d**) C57BL/6 mice (7–10-week-old) were gavaged with fecal homogenates (OD_600_ = 20) derived from *Notch2*^cKO^ mice. CD64^−^AXL^+^inf-DC3 and CD64^+^AXL^+^inf-DC3 among DC2/DC3, pre-gated as in Extended Data Fig. 2g, in CoLP, mLN and spleen were analyzed by flow cytometry at each timepoint in C57BL/6 (dark blue), C57BL/6 gavaged with *Notch2*^cKO^ fecal material (light blue), and *Notch2*^cKO^ (orange). (**a–d**) Frequencies (left) and total cell numbers (right) of the indicated populations are shown for each genotype and condition in the CoLP (**a**), mLN (**b**), and spleen (**c, d**). (Sample sizes for each group are indicated from left to right. CoLP/mLN/spleen: *n* = 7, 7, 9, and 3 mice, pooled data from two independent experiments). (**e–h**) Antibiotics were administered via drinking water of 15-20-week-old *Notch2*^fl/fl^ (dark blue), *Notch2*^fl/fl^* co-housed with *Notch2*^cKO^ (light blue), and *Notch2*^cKO^ (orange) for 9 days, followed by regular water for 2 weeks (until day 23). Shown are frequencies (left) and total cell numbers (right) of CD64^−^AXL^+^inf-DC3 and CD64^+^AXL^+^inf-DC3, pre-gated as in Extended Data Fig. 2g, in CoLP (**e**), mLN (**f**) and spleen (**g, h**). (CoLP (time-point): *n* = 6, 4, and 7 or 8 mice, mLN (time-point): *n* = 4 or 5, 4, and 7 or 8 mice, spleen (time-point): *n* = 4 or 5, 4, and 8 mice, pooled data from two independent experiments). (**i–m**) Shown are frequencies (left) and total cell numbers (right) of CD64^−^AXL^+^inf-DC3 and CD64^+^AXL^+^inf-DC3 among DC2/DC3, pre-gated as in Extended Data Fig. 2g, in CoLP (**i**), mLN (**j**) and spleen (**m**) analyzed in 8-12-week-old *Notch2*^fl/fl^, *Notch2*^cKO^, and rederived *Notch2*^cKO^ mice as indicated. (*n* = 8, 3 and 4 or 5 mice, pooled data from two independent experiments). (**k**) Pie charts showing average DC subset frequencies for the indicated subsets in the spleen. (**n**) BM chimeras were generated as described in Fig. 7k. Quantification of CD64^+^AXL^+^inf-DC3, pre-gated as in Extended Data Fig. 2g, in the indicated tissues. (Sample sizes are indicated from left to right. *n* = 6, 11, 8, 3, and 3 mice, pooled data from two independent experiments). (**o**) C57BL/6 mice (7–10-week-old) were gavaged with fecal homogenates obtained from *Notch2*^cKO^ mice (OD_600_ = 20) and anti-ssDNA IgG (top), IgG2c (middle), and IgA (bottom) antibody titers were measured at the indicated timepoints by ELISA. Steady-state C57BL/6 and *Notch2*^cKO^ (day 0), and C57BL/6 gavaged (day 9, 30). Data are presented as arbitrary units (a.u.). (day 0: *n* = 16 and 3 mice, day 9: *n* = 7 mice, day 30: *n* = 9 mice, pooled data from two independent experiments). Data are shown as mean ± SD. Statistical analysis was performed using an unpaired two-tailed *t*-test. *P > 0.05, **P > 0.01, ***P > 0.001, ****P > 0.0001. Source data

AXL^+^inf-DC3 expansion correlated with both pathobiont exposure and intestinal barrier disruption, although its contribution to the establishment of the inflammatory response remained unclear. We first analyzed the kinetic of DC expansion following oral gavage with the dysbiotic community. Already by day 9, AXL^+^inf-DC3 frequencies and numbers in the colon and mLN reached equal levels as observed in *Notch2*^cKO^ mice and remained elevated thereafter (Fig. 7a–c). However, only AXL^+^inf-DC3 expressing CD64 expanded following microbiota transfer, supporting a specialized functional role by this subset. In contrast, the increased frequency of CD64^−^AXL^+^inf-DC3 in *Notch2*^cKO^ mice may reflect a genotype-driven expansion that could contribute to increased susceptibility to intestinal pathobionts and increased disease severity upon DSS treatment (Extended Data Fig. 8a–d).

Further supporting a specialized role for AXL^+^inf-DC3s in mediating host responses to intestinal dysbiosis, antibiotic treatment reduced bacterial burden and antibody titers (Fig. 6b–d) and was accompanied by a marked contraction of the AXL^+^inf-DC3 pool in both dysbiotic WT and *Notch2*^cKO^ mice (Fig. 7d–f). Also in this context, the effect was largely restricted to the CD64^+^ fraction, whereas CD64^−^ cells remained unaltered, suggesting that these two subsets are regulated through distinct pathways and that the expansion of latter is genetically driven (Extended Data Fig. 8e–h).

To gain a better understanding of the genetic and pathobiont contribution, we rederived and analyzed *Notch2*^cKO^ under specific-pathogen-free conditions (Red-*Notch2*^cKO^). AXL^+^inf-DC3 were expanded in these mice. However, their expansion across intestinal tissues was limited to the CD64^−^ fraction, suggesting a genetically driven and microbiome independent process (Fig. 7g–j and Extended Data Fig. 8i–m). To determine whether AXL^+^inf-DC3 expansion was cell-intrinsic or driven by extrinsic factors, we generated WT, *Notch2*^cKO^ and mixed (1:1) BM chimeras and analyzed the DC compartment after 8 weeks. Of note, mice were kept under antibiotic treatment (Fig. 7k). In line with the above results, exclusively the CD64^−^ fraction of AXL^+^inf-DC3 expanded, but only if donor cells were *Notch2* deficient. In mixed chimeras, donor-derived cells were predominantly *Notch2*^cKO^ derived, indicating a cell-intrinsic competitive advantage (Fig. 7l,m and Extended Data Fig. 8n). Aside from the expected lack of cDC2a, other DC subsets were largely unaffected. However, several peripheral DC populations showed preferential contribution of cKO-derived cells in mixed chimeras, suggesting a broader competitive advantage (Supplementary Fig. 5a,b). The colonic B cell compartment was unaffected, showing equal contribution by WT and cKO cells (Supplementary Fig. 5c).

Collectively, our data show that the expansion of CD64^−^AXL^+^inf-DC3s is driven by *Notch2* deficiency and is microbiota independent, whereas CD64^+^AXL^+^inf-DC3s tracked pathobiont colonization, expanding after microbiota transfer and contracting upon antibiotic treatment, with kinetics that parallel antibody and autoantibody responses (Extended Data Fig. 8o).

### Dysbiosis-expanded AXL^+^inf-DC3s promote autoimmunity

**Fig. 8:**
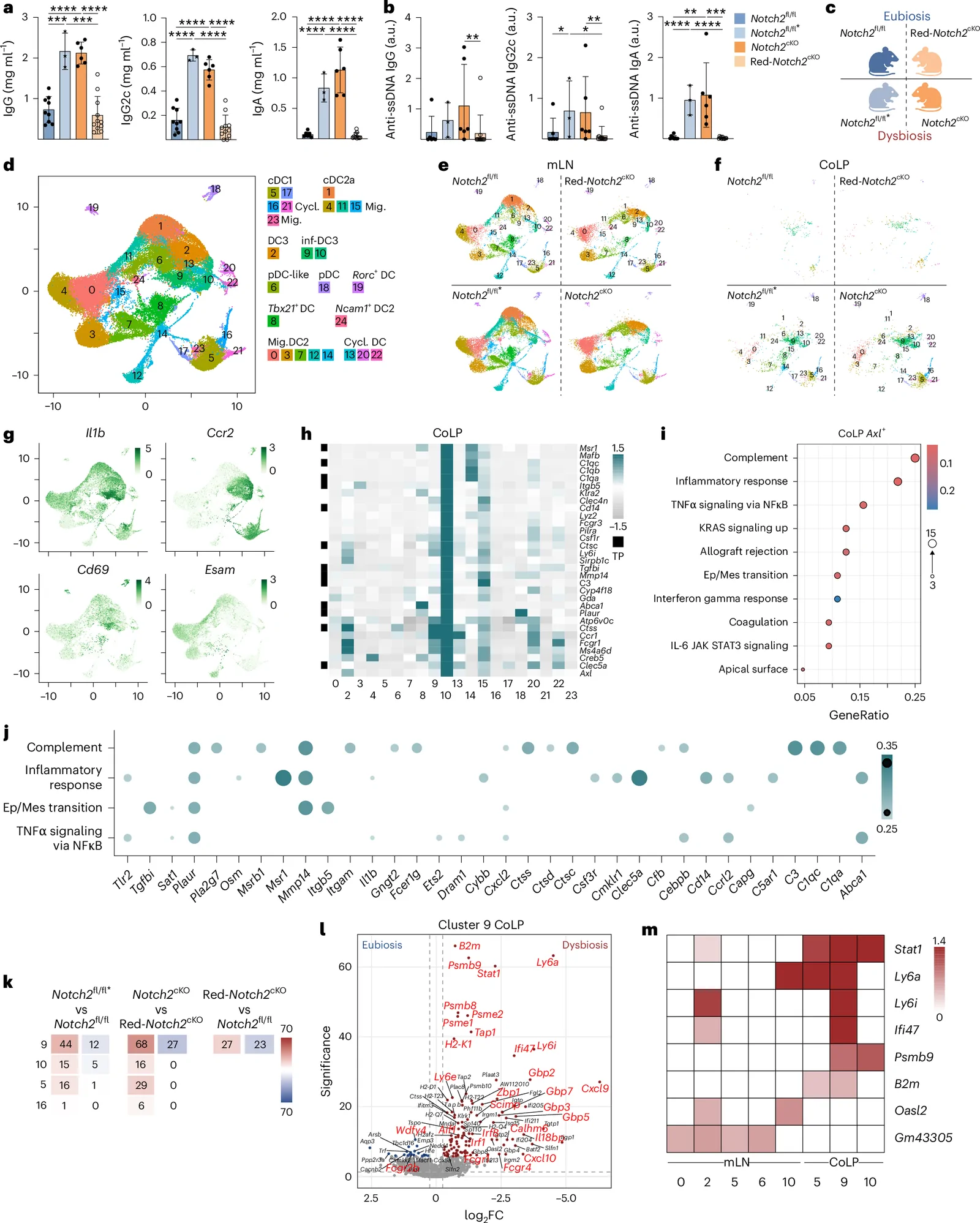
Dysbiosis-expanded AXL^+^inf-DC3s promote autoimmunity. **a**, Serum IgG (left), IgG2c (middle), and IgA (right) concentrations in 8- to 12-week-old *Notch2*^fl/fl^ (dark blue), *Notch2*^fl/fl^* co-housed with *Notch2*^cKO^ (light blue), *Notch2*^cKO^ (orange) and rederived *Notch2*^cKO^ (light orange) mice were determined by ELISA. Sample sizes for each group are indicated from left to right (*n* = 9, 3, 6, and 11 mice, pooled data from at least three independent experiments). **b**, Serum anti-ssDNA specific IgG (left), IgG2c (middle) and IgA (right) antibody concentrations in 7- to 12-week-old *Notch2*^fl/fl^ (dark blue), *Notch2*^fl/fl^* co-housed with *Notch2*^cKO^ (light blue), *Notch2*^cKO^ (orange) and rederived *Notch2*^cKO^ (light orange) were measured by ELISA. Data are presented as arbitrary units (a.u.). Sample sizes for each group are indicated from left to right (*n* = 6 or 9, 3, 6, and 11 mice, pooled data from at least three independent experiments). Data are shown as mean ± s.d. Statistical analysis was performed using an unpaired two-tailed *t*-test (**a**) and a two-sided Mann^−^Whitney U test (**b**). *P > 0.05, **P > 0.01, ***P > 0.001, ****P > 0.0001. **c**–**m**, scRNA-seq was performed on sort-purified Lin^−^CD11c^+^CD26^+^MHC-II^+^ DC from the mLN and CoLP of 8- to 12-week-old *Notch2*^fl/fl^ (dark blue), rederived *Notch2*^cKO^ (light orange), *Notch2*^fl/fl^* co-housed with *Notch2*^cKO^ (light blue) and *Notch2*^cKO^ (orange) mice. Details are provided in Methods. **c**, Representation of the genotype and condition plotting scheme. **d**, UMAP showing cluster distribution including cells from each genotype and tissue. Mig. DC, migratory DCs; Cycl. DC, cycling DCs. **e**,**f**, UMAP showing cluster distribution for each genotype separately in the (**e**) mLN and (**f**) CoLP. **g**, UMAP showing relative expression levels of the indicated genes including cells from each genotype and tissue. **h**–**j**, Genes positively correlated with *Axl* were identified using Spearman rank correlation (ρ≥0.2) across CoLP cells. **h**, Heatmap of genes that are co-expressed with *Axl* across all CoLP clusters. Values represent row-scaled (z-score) average expression per cluster. TP, present in top pathways. **i**, Pathway enrichment analysis for all genes correlating with all *Axl* expressing cells within the CoLP. The most enriched Hallmark pathways are displayed as dot plot. Analysis was performed using the Molecular Signatures Database (MSigDB V7.5.1). Dot size represents the number of core enriched genes. Color indicates the adjusted *P* value. GeneRatio represents the proportion of input genes overlapping each pathway. **j**, Dot plot showing genes within the top enriched CoLP pathways. Dot size and color represent the Spearman correlation coefficient (ρ). **k**, Number of upregulated (red) and downregulated (blue) DEGs between the indicated genotypes and conditions across CoLP clusters. **l**, Volcano plot showing the top DEGs between eubiotic *Notch2*^fl/fl^/Red-*Notch2*^*c*KO^ and dysbiotic *Notch2*^fl/fl^*/*Notch2*^cKO^ cluster 9 (colonic inf-DC3). Significance is represented as −log_10_(adjusted *P* value). **m**, Heatmap of all dysbiosis induced genes that are upregulated in at least two clusters when comparing each dysbiotic to eubiotic conditions. Color scale represents the minimal log_2_ fold change across comparisons. White indicates no induction. Source data

Taking advantage of the Red-*Notch2*^cKO^ model, we established that *Notch2* deficiency selectively promoted the expansion of the CD64^−^AXL^+^inf-DC3 fraction, whereas dysbiosis in both WT and cKO mice drove expansion of the CD64^+^ fraction. Given that *Notch2* deficiency broadly altered gene expression across cDC subsets, including reduced expression of *Dnase1l3*, we asked whether autoimmune features would persist in Red-*Notch2*^cKO^ mice in the absence of dysbiosis. Surprisingly, Red-*Notch2*^cKO^ mice lacked elevated antibody and autoantibody titers (Fig. 8a,b), establishing pathobiont exposure as the critical trigger for autoimmune manifestations in both WT and cKO mice.

**Extended Data Fig. 9:**
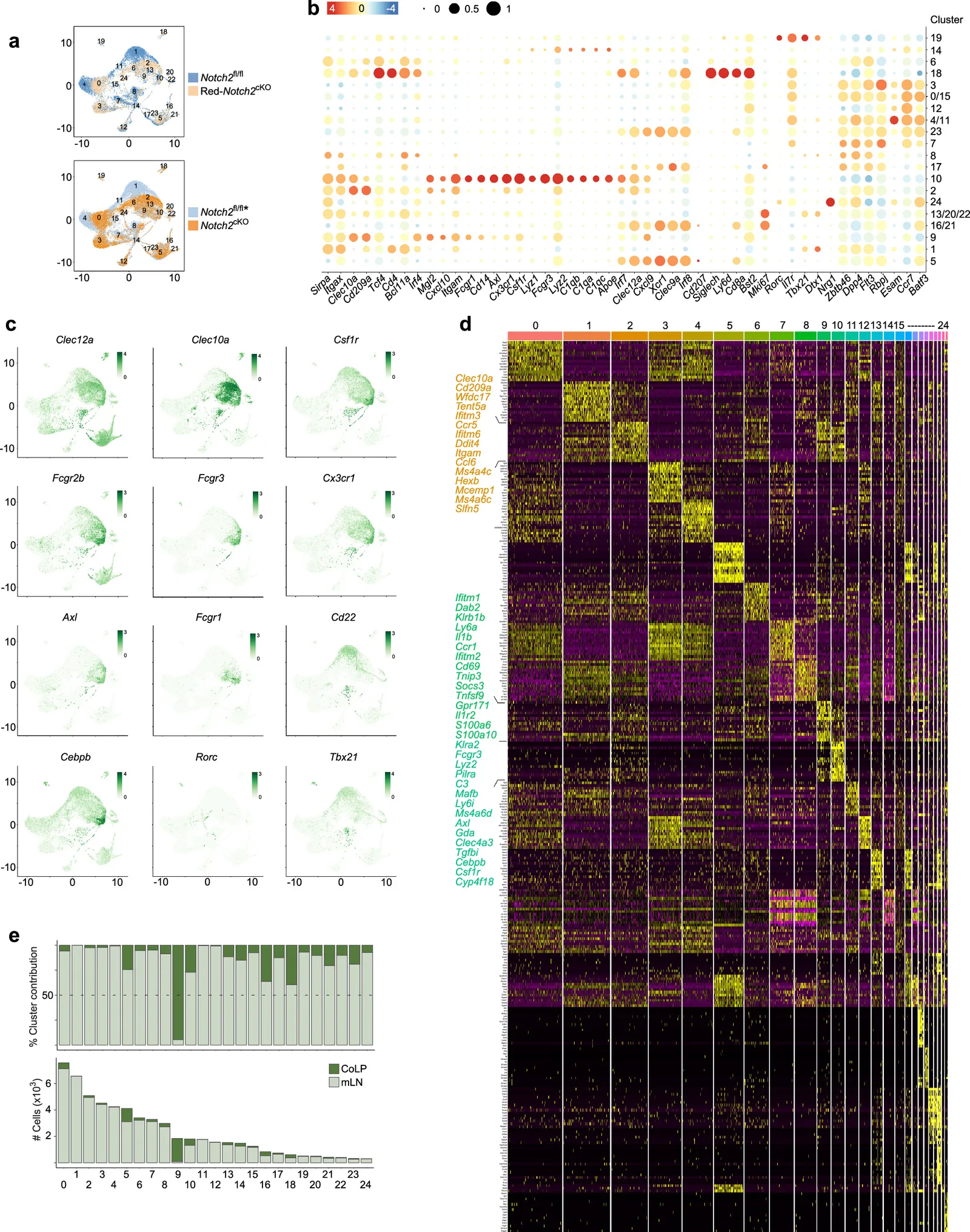
Dysbiosis-expanded AXL^+^inf-DC3 promote autoimmunity. Related to Fig. 8. (**a-e**) Cells were prepared as described in Fig. 8c. (**a**) UMAP showing all cells colored based on genotype. (**b**) Dot plot showing the relative expression levels of the indicated genes across all clusters. Dot size represents the proportion of expressing cells, and color indicates the relative expression level. (**c**) UMAP showing relative expression levels of the indicated genes including cells from each genotype and tissue. (**d**) Heatmap showing relative expression levels of the top 15 DEGs with a detection frequency >50% for each genotype and tissue. (**e**) Contribution by frequency (top) and total cell numbers (bottom) of each tissue to the overall cluster composition is plotted. Source data

**Extended Data Fig. 10:**
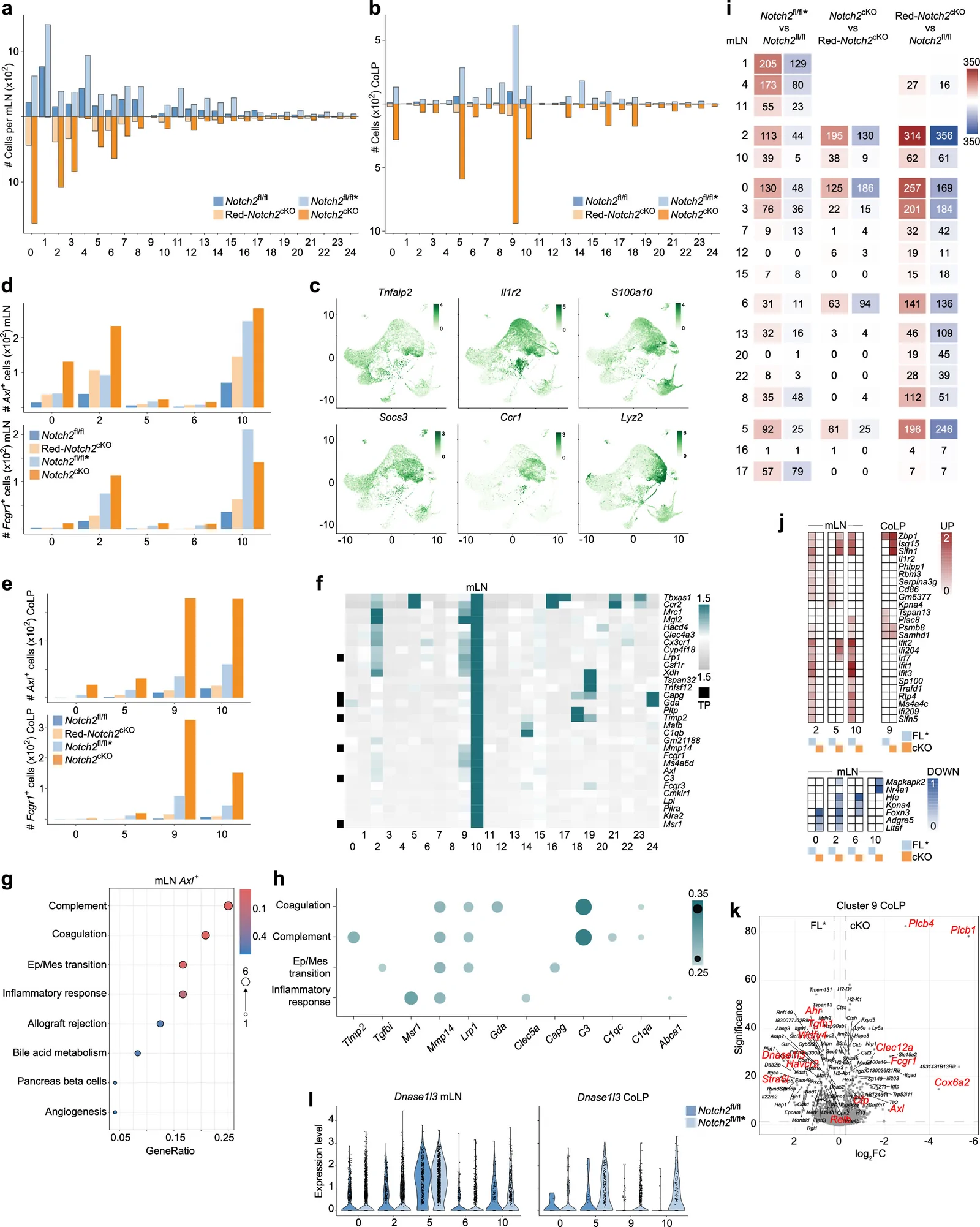
Dysbiosis-expanded AXL^+^inf-DC3 promote autoimmunity. Related to Fig. 8. (**a-b**) Contribution of the indicated genotypes to each cluster is plotted as total cell numbers in mLN (**a**) and CoLP (**b**). (**c**) UMAP showing relative expression levels of the indicated genes including cells from each genotype and tissue. (**d-e**) Total number of *Axl* (top) or *Fcgr1* (bottom) expressing cells within the indicated mLN (**d**), and CoLP (**e**) clusters across all genotypes. (**f-h**) Genes positively correlated with *Axl* were identified using Spearman rank correlation (ρ≥0.2) across mLN cells. (**f**) Heatmap of genes that are co-expressed with *Axl* across all mLN clusters. Values represent row-scaled (z-score) average expression per cluster. TP: Present in top pathways. (**g**) Pathway enrichment analysis for all genes correlating with all *Axl* expressing cells within the mLN. The most enriched Hallmark pathways are displayed as dot plot. Analysis was performed using the Molecular Signatures Database [MSigDB (V7.5.1)]. Dot size represents the number of core enriched genes. Color indicates the adjusted *P* value. The GeneRatio represents the proportion of input genes overlapping each pathway. (**h**) Dot plot showing genes within the top enriched mLN pathways. Dot size and color represent the Spearman correlation coefficient (ρ). (**i**) Number of (red) upregulated and (blue) downregulated DEGs between the indicated genotypes and conditions across mLN clusters. (**j**) Heatmap of all dysbiosis upregulated (red) and downregulated (blue) genes that are altered in at least two clusters and one dysbiotic condition relative to each eubiotic condition. Color scale represents the minimal log_2_ fold change across comparisons. White indicates no induction. (**k**) Volcano plot showing the top DEGs between control *Notch2*^fl/fl^* and deficient *Notch2*^cKO^ cluster 9 (colonic inf-DC3). Significance is represented as -log_10_(adjusted *P* value). (**l**) Violin plots showing log-normalized gene expression levels for *Dnase1l3* across the indicated clusters and all genotypes in mLN (left) and CoLP (right). Source data

Given the close association between dysbiosis and AXL^+^inf-DC3 expansion, we performed scRNA-seq on DC isolated from colon and mLN of eubiotic WT (*Notch2*^fl/fl^) and rederived cKO (Red-*Notch2*^cKO^) mice, as well as dysbiotic WT (*Notch2*^fl/fl^) and cKO (*Notch2*^cKO^) mice (Fig. 8c). Approximately 60,000 mLN and 6,000 colonic cells from three mice per group were analyzed. All cells were analyzed jointly, and cluster identities were annotated using canonical and cluster-defining genes (Fig. 8d–f, Extended Data Fig. 9a–d and Supplementary Table 5). We identified 25 clusters (0–24) distributed across tissues and conditions (Extended Data Fig. 9e and Extended Data Fig. 10a,b). Consistent with previous analyses, cDC2a (cluster 1) and their migratory counterparts (clusters 4 and 11) were absent in *Notch2*^cKO^. pDC (cluster18) and *Rorc*^+^ DCs (cluster 19) formed distinct populations, whereas pDC-like cells (cluster 6) remained embedded within the cDC compartment. Several clusters displayed proliferative signatures (clusters 13, 16 and 20–22).

Clusters 2, 9 and 10 classified as DC3s based on *Fcgr2b*/*Fcgr3* (CD16/CD32) and *Cx3cr1* expression (Extended Data Fig. 9b–d). Clusters 9 and 10 were enriched for inflammatory and antimicrobial transcripts, consistent with inf-DC3 identity (Fig. 8g and Extended Data Figs. 9b,d and 10c). Expression of *Axl* and *Fcgr1* (CD64) further resolved the AXL^+^inf-DC3 subsets (Extended Data Figs. 9c and 10d,e). Cluster 9 represented CD64^+^AXL^+^inf-DC3, showing the prominent expansion in the colon of dysbiotic WT and cKO mice. Cluster 10 corresponded to the genetically expanded CD64^−^AXL^+^inf-DC3 population. Although inflammatory genes such as *Il1b* were expressed across all DC3 clusters (clusters 2, 9 and 10), AXL^+^inf-DC3 (clusters 9 and 10) displayed the strongest inflammatory signature, with high expression of complement genes and genes related to antibacterial functions (Fig. 8h–j and Extended Data Figs. 9b and 10f–h). Importantly, cluster 9 was restricted to the CoLP, compatible with a specialized function in sensing pathobiont growth, whereas clusters 2 and 10 were also present in mLN (Extended Data Figs. 9e and 10a,b).

Cell counts corroborated flow cytometry data, confirming expansion of multiple DC subsets in dysbiotic mice regardless of genotype, consistent with marked enlargement of mLN, PP and cecal patches (Extended Data Figs. 10a,b, 3q and 5i–l). Colonic clusters responding to dysbiosis included clusters 0, 5, 9 and 10 (Extended Data Fig. 10b), which expressed activation-, migration- and lymphoid-organizing genes, including *Gcsam*, *Ccr2*, *Ccr7*, *Ccl5, Ccl6, Ccl9*, *Cx3cr1*, *Ccr1* and *Ccr5* (Extended Data Fig. 9b–d). Among these, AXL^+^ inf-DC3s (clusters 9 and 10) exhibited the strongest transcriptional response to dysbiosis (Fig. 8k). Comparison of cluster 9 under eubiotic and dysbiotic conditions revealed induction of interferon- and innate immunity-associated genes, including guanylate binding proteins, together with enhanced antigen processing and presentation programs (Fig. 8l and Supplementary Table 6). Although dysbiosis-induced changes were detected in both tissues (Extended Data Fig. 10i), the shared signature was limited and included *Stat1* and *Psmb9*, genes linked to autoimmunity and chronic inflammatory disease^55^, which were prominently induced in colonic AXL^+^inf-DC3 (Fig. 8m). Comparison of dysbiotic WT and cKO cells identified *Notch2*-dependent transcriptional alterations beyond baseline genotype effects (Extended Data Fig. 10j,k), including colonic *Dnase1l3* induction in WT mice (Extended Data Fig. 10l).

Collectively, these data identify AXL^+^inf-DC3s as the principal dysbiosis-responsive population, whose transcriptional program supports a chronic inflammatory state and enhanced presentation of microbial and putative tissue-derived antigens, thereby driving autoimmune manifestations.

## Discussion

DC3s were recently identified as a distinct DC subset that is transcriptionally related to, yet developmentally distinct from, monocyte-derived DCs^29^. Their expansion in patients with systemic lupus erythematosus (SLE)^30^ suggested a potential role in autoimmune disease, but their tissue distribution, functional specialization, and pathogenic relevance remained poorly understood. In the present study, we demonstrate that *Notch2* deficiency profoundly remodels the DC compartment beyond the previously reported loss of ESAM^+^ cDC2a cells^16,17^. The most prominent alteration was the expansion of a DC3 population enriched for inflammatory, immunoproteasome, complement and scavenger receptor-associated genes, which we termed AXL^+^inf-DC3s. This subset was present at low frequency in gut-associated lymphoid tissues of WT mice but accumulated extensively in *Notch2*-deficient animals, where they localized adjacent to activated epithelial structures and tertiary lymphoid aggregates enriched in CD4^+^ T cells and IgA-producing plasma cells. Their transcriptional profile and tissue distribution suggest specialization at mucosal barrier sites. Given the established scavenger functions of AXL in macrophages^56^ and its expression by regulatory DC populations in tumors^15^, AXL^+^inf-DC3s are likely involved in sensing and clearing dying cells, microbial products or infected epithelial cells. Their enrichment for antigen processing and presentation pathways, together with their close association with adaptive immune cells, further supports a role in coordinating immune responses at the intestinal barrier.

Initially, we hypothesized that the expansion of AXL^+^inf-DC3s and the autoimmune manifestations observed in *Notch2*^cKO^ mice resulted primarily from intrinsic defects in DC function. This interpretation was supported by the marked reduction of *Dnase1l3* expression across multiple DC subsets. DNASE1L3 is essential for extracellular DNA clearance, and a loss-of-function mutation was associated with a familial early-onset of SLE^57–60^. Consistent with this, *Notch2*-deficient mice developed elevated antibody titers and immune complex deposition in the kidney, phenotypes reminiscent of *Dnase1l3*-deficient mice. However, several observations suggested a more complex mechanism. In addition to renal immune pathology, *Notch2*^cKO^ mice displayed abnormalities concentrated at intestinal barrier sites, including increased epithelial activation, tertiary lymphoid structure formation and increased intestinal permeability. Although FITC-dextran assays demonstrated impaired barrier integrity, we were unable to detect systemic bacterial dissemination, suggesting that disease may be driven by enhanced exposure to microbial products rather than overt translocation of viable bacteria. Furthermore, recent evidence implicates DNASE1L3 together with DNASE1 in biofilm degradation^60^, raising the possibility that impaired DNASE1L3 expression may facilitate colonization by biofilm-forming pathobionts^17^.

Unexpectedly, co-housing and fecal transfer experiments revealed that autoimmune features also developed in *Notch2*-sufficient recipients. Thus, dysbiosis rather than *Notch2* deficiency was sufficient to induce disease manifestations. These findings indicate that *Notch2* deficiency may have acted primarily as a susceptibility factor, facilitating the establishment of a pathogenic microbial community, likely through a combination of impaired T_H_17 immunity^16,17^, defective microbial control and reduced DNASE1L3 mediated clearance mechanisms. Once established, however, the dysbiotic community could transfer inflammatory and autoimmune features to immunocompetent hosts without antibiotic preconditioning.

The discovery of a transferable pathobiome provided a unique opportunity to identify candidate microbial drivers. Although dysbiosis has long been associated with autoimmune diseases including inflammatory bowel disease, rheumatoid arthritis and SLE, the specific organisms and mechanisms involved remain controversial^53,61–66^. Our analysis identified three candidate pathobionts, *Mucispirillum schaedleri* MGBC140179, *Chlamydia muridarum* and *Parasutterella* MGBC134540, that consistently associated with disease transmission and autoimmune manifestations^43,45–48,67–72^.

Each of these organisms has previously been linked to dysbiosis or inflammatory disease. Particularly notable was the enrichment of *M. schaedleri* accompanied by loss of the health-associated commensal *Akkermansia muciniphila*. Because *A. muciniphila* promotes mucosal tolerance and supports short-chain fatty acid-mediated immune regulation, its replacement by *M. schaedleri* may shift the intestinal environment from a tolerogenic to a proinflammatory state^43,46,47,67–69^. Consistent with this possibility, *Notch2*-deficient DCs exhibited reduced expression of short-chain fatty acid-responsive genes, potentially further amplifying inflammatory responses. *C. muridarum* is particularly intriguing given its longstanding association with chronic inflammatory and autoimmune disorders. Persistent infection, chronic activation of immune responses and molecular mimicry have all been proposed as mechanisms linking *Chlamydia* species to autoimmunity. Further, its identification at autoimmune lesions also supports a direct role to autoimmune pathogenesis^48,70–72^. Similarly, *Parasutterella* has repeatedly been associated with dysbiosis and reduced microbial diversity and has been detected within inflamed intestinal tissues^45^. However, whether disease induction reflects activity of individual species, synergistic interactions among them or broader alterations of the microbial community remains unresolved. Nevertheless, the fact that transfer of this microbial consortium reproduced intestinal inflammation, AXL^+^inf-DC3 expansion, autoantibody production and immune complex deposition strongly supports a causal contribution of the pathobiome.

Several observations further suggest that microbial stimulation and AXL^+^inf-DC3 expansion are mechanistically linked. Rederivation of *Notch2*^cKO^ mice allowed us to distinguish genetic from microbiota-dependent regulation of this population. Although expansion of the CD64^−^ fraction reflects a cell-intrinsic consequence of *Notch2* deficiency, the expansion of CD64-expressing AXL^+^inf-DC3 closely tracked microbial burden and pathobiont colonization. Broad-spectrum antibiotic treatment substantially reduced both AXL^+^inf-DC3 abundance and autoantibody titers, indicating that this population responds directly to microbial perturbation and may contribute to downstream humoral immune activation. The observation that DSS-induced barrier disruption alone failed to reproduce the autoimmune phenotype further suggests that disease is not simply a consequence of increased permeability but instead requires specific microbial stimuli. Nevertheless, the precise contribution of AXL^+^inf-DC3s to disease remains to be established. Their expansion may initially represent a protective response aimed at containing pathobionts through scavenging, antigen capture and immune activation. However, chronic stimulation appears to convert this response into a self-sustaining inflammatory circuit. Equipped with multiple Fc receptors, scavenger receptors, complement-associated molecules and antigen-presentation machinery, AXL^+^inf-DC3s are ideally positioned to sense microbial perturbations and amplify adaptive immune responses. Whether they directly promote autoantibody generation through molecular mimicry, loss of tolerance or persistent inflammatory signaling remains an important question for future investigation. Furthermore, it will be important to determine whether direct targeting of this subset could provide therapeutic benefit in established autoimmune conditions. It will also be important to assess whether transfer of this dysbiotic microbial community to autoimmune-prone strains exacerbates or accelerates disease development, thereby helping to explain the variability in susceptibility to autoimmunity. Such susceptibility is known to have a strong, albeit complex, genetic component, as evidenced by familial concordance.

The developmental origin of AXL^+^inf-DC3s also remains unresolved. Although these cells share transcriptional features with monocytes and monocyte-derived DCs, several observations argue against a conventional monocytic origin. They differentiate in vitro in response to FLT3L, express multiple DC-associated markers, lack expression of canonical monocyte-derived DC markers such as CD88 and iNOS^14,73^ and exhibit intermediate labeling in *Ms4a3* fate-mapping models. Importantly, labeling frequencies remain unchanged during dysbiosis-driven expansion, arguing against substantial monocyte recruitment. Nevertheless, the distinction among inflammatory DC, cDC2b, DC3 and monocyte-associated lineages remains incompletely resolved^16,18,20,29^, particularly under inflammatory conditions where progenitor plasticity may reshape developmental trajectories.

Collectively, our findings identify AXL^+^inf-DC3s as a previously unrecognized intestinal DC3 subset that directly responds to microbial perturbations and links dysbiosis to chronic adaptive immune activation. We propose a model in which genetic susceptibility factors, such as impaired *Notch2* signaling, facilitate colonization by specific pathobionts. These microbes drive expansion of AXL^+^inf-DC3s, chronic mucosal inflammation and sustained antibody responses that ultimately culminate in systemic autoimmune manifestations. More broadly, our study provides mechanistic evidence that specific pathobionts, rather than barrier dysfunction alone, can promote autoimmune pathology through activation of specialized mucosal DC populations. Targeting this host–microbiome axis may therefore represent a promising therapeutic strategy for autoimmune diseases driven by chronic intestinal inflammation.

## Methods

### Mice

Mice were maintained in the specific-pathogen-free AAALAC-accredited NIDCR animal facility according to institutional guidelines (National Institutes of Health, license number 22-1111, 21-1063, LCIM-6). Housing conditions: 20-24.5°C, 30-70% humidity, 14 h light/10 h dark cycle. The following strains were bred and maintained on a C57BL/6 background: C57BL/6J, CD45.1, *Notch2*^fl/fl^, *Notch2*^fl/fl^*CD11c*^Cre^, *Klf4*^fl/fl^*CD11c*^Cre^, *Flt3l*^−/−^, *Ccr2*^−/−^ and *Rag2*^−/−^; the respective parental strains were originally purchased from the Jackson Laboratory. *Klf4*^fl/fl^ mice were obtained from Mutant Mouse Resource and Research Center (line 29877). Serum from *Aire*^−/−^ mice was obtained from M. Lionakis. Serum from *Ifng* 3′UTR-deleted adenylate-uridylate-rich element (ARE-del^−/−^) was obtained from H. Young. Mice that were sex- and age-matched were used in the following age groups unless otherwise indicated: (i) young, 8 to 12 weeks of age; (ii) middle, 25 to 30 weeks of age; and (iii) old, over 50 weeks of age. Pilot experiments were used to estimate the sample sizes required to achieve adequate statistical power. Animals were randomly assigned to experimental groups based on genotype and age, and samples were processed in parallel under identical experimental conditions. All data points were included in the analyses except in cases where sample preparation failed because of technical issues. Data collection and analysis were not performed blind to the conditions of the experiments.

### Co-housing and cage-switching

Mice were co-housed by placing selected animals in the same cage. Unless otherwise stated, co-housing was initiated after weaning. Cage switching was performed by transferring selected mice into a used donor cage containing residual fecal material for three consecutive days.

### BM chimera

For BM chimera experiments, 6-week-old CD45.1 recipient mice received a total body irradiation (2 × 4 Gy) using a cesium-137 irradiator. The following day, total BM cells were isolated from 12-week-old donor CD45.1/.2 or CD45.2 *Notch2*^cKO^ mice. A total of 5 × 10^6^ BM cells were intravenously injected per recipient, either from each individual donor or a 1:1 mixture of both. Antibiotic trimethoprim-sulfamethoxazole water was maintained for 8 weeks from irradiation up to the harvest.

### Antibiotic treatment

Mice were treated with an antibiotic cocktail (0.01 mg ml^−1^ amphotericin-B; 1 mg ml^−1^ ampicillin; 0.5 mg ml^−1^ vancomycin; 1 mg ml^−1^ neomycin, 1 mg ml^−1^ metronidazole). Peripheral blood samples were collected at days 0, 9 and 23.

### DSS-induced colitis

Drinking water was supplemented with 2.5% DSS for 7 days followed by regular water for 6 days. Weight loss was monitored for 13 days, and mice losing more than 20% body weight were euthanized. Blood was collected at days 0, 9 and 13. Colon tissues were processed for H&E staining as described below. Tissue infiltration was quantified from longitudinal center-cut sections, and muscle thickness was measured bilaterally in feces-distended regions and averaged per sample.

### FITC-Dextran gavage

Mice were fasted for 4 to 6 h before and during the experiment. FITC-Dextran (FD4; 600 mg kg^−1^) was administered by oral gavage in 200 μl PBS. Blood was collected 4 h later, and plasma fluorescence was measured at 490 nm excitation and 520 nm emission.

### Immunohistochemistry

Frozen spleens, kidneys and colons were embedded in O.C.T., stored at −80 °C, sectioned at 8μm, and mounted as individual or consecutive sections onto Superfrost Plus slides. Sections were incubated at 22 °C for 30 min, fixed in ice-cold acetone for 10 min, air-dried and stored at −30 °C. Pre-warmed slides were rehydrated in PBS for 5 min, blocked in MACS buffer containing 5% goat serum, stained for 2 to 3 h at 22°C, and mounted in Prolong Gold. Spleen and intestine slides were acquired using a Leica SP8 confocal (white light laser, ×20 (N.A. 0.8) objective, controlled by LASX software). For kidneys, a widefield Nikon TiE (8-line Spectra Light engine, Hamamatsu BT fusion camera, Sutter Instruments filter wheel) was used. All images were analyzed using FIJI. For paraffin sections, tissues were fixed in Carnoy’s fixative for approximately 24 h at 4 °C, washed in 70% EtOH, paraffin embedded and sectioned at 8 μm.

H&E staining slides were deparaffinized in xylene for 2 min, rehydrated in descending alcohol series (100%, 95% and 70% EtOH solutions) for 4 min each, immersed in water for 5 min at 22 °C and stained with hematoxylin for 5 min. Following two washes, slides were immersed in Differentiation Solution for 1 min, washed, placed in Bluing Reagent for 1 min and washed again. Slides were then soaked in 100% EtOH for 30 sec and immediately immersed in Eosin-Y stain for 2 min. Slides were dehydrated in ascending alcohol series (70%, 95% and 100% EtOH solutions) for 4 min each and placed in xylene for 2 min. Once dried, the stained slides were Cytoseal mounted. H&E images were acquired with the S60 NanoZoomer Digital Scanner by Hamamatsu at ×40 magnification.

For fluorescent in situ hybridization, paraffin sections were melted for 30 min in a hybridization oven at 60 °C, deparaffinized in xylene, rehydrated in two ethanol washes (95% and 90%), and H_2_O for 10 min. Slides were hybridized overnight at 50 °C in hybridization buffer (0.9 M NaCl, 20 mM Tris-HCl, 0.1% SDS, pH 7.2) containing standard Eubacteria (1μM, EUB338-AF647, 5′-GCTGCCTCCCGTAGGAGT-3′) and *Chlamydia* (1μM, Chla232-AF555, 5′- TAGCTGATATCACATAGA-3′) probes. Sections were washed at each step (0.9 M NaCl, 20 mM Tris-HCl, pH 7.2), stained with DAPI, mounted in Prolong Gold and imaged on a Leica SP8 confocal microscope (×20).

Immunofluorescence staining on paraffin sections was achieved as follows. Paraffin was melted for 30 min at 60 °C. Slides were then rehydrated in xylene, 90% EtOH, 70% EtOH and H_2_O. Slides were subsequently transferred to PBS for IHC-F staining procedure as described above.

### Blood collection

Blood was collected by retro-orbital bleeding, or terminal intracardiac puncture. Serum was isolated using clot activator containing BD Microtainer serum separation tubes according to the manufacturer’s instructions.

### Immunoglobulin ELISA

Serum antibodies were analyzed by ELISA using MaxiSorp 96-well plates coated o/n at 4°C with unconjugated goat anti-mouse antibody isotypes (4 μg ml^−1^). Plates were washed with PBS/0.05% Tween 20, blocked with PBS/4% BSA for 1 h at 22°C, and incubated with samples for 2 to 3 h at 22 °C. Plates were washed with TBS/0.05% Tween 20 and bound antibodies were detected using alkaline phosphatase-conjugated goat anti-mouse isotype antibodies (2 μg ml^−1^) for 2 h at 22 °C. pNPP (1 mg ml^−1^) in substrate solution (0.1 M glycine, 1 mM MgCl_2_, 1 mM ZnCl_2_, pH 10.4) was used for colorimetric detection. Absorbance was measured at OD_405_ on a SpectraMaxPlus 384. Standard curves were generated in GraphPad Prism (v.10), using a four-parameter logistic sigmoidal curve. For ssDNA and dsDNA autoantibodies, plates were pre-coated with poly-D-lysine (0.05 mg ml^−1^) for 8 to 10 h at 4°C. Plates were washed with PBS and incubated with 10 μg ml^−1^ calf thymus DNA (ThermoFisher) or calf thymus ssDNA (Sigma) overnight at 4°C. Detection of serum immunoglobulin was performed as explained above. Relative quantification, reported as arbitrary units (a.u.), was determined using a standard curve generated from serial threefold dilutions of the corresponding isotype starting at 0.33 μg ml^−1^. Serum antibodies against *C. muridarum* were assessed by ELISA as described above using Immulon 2HB microtiter plates coated with 0.1 ml of 10 μg ml^−1^ *C. muridarum* elementary bodies^74^.

### Systemic autoimmune-associated antigen array

Serum IgG and IgM autoantibodies were screened by GeneCopoeia using the OmicsArray systemic autoimmune-associated antigen array containing 120 antigens and 8 internal controls according to the manufacturer’s protocol. Signal to noise ratios were calculated for each antigen, with a ratio >3 considered to be above background.

### Immune cell isolation

Myeloid cell suspensions from spleen, cervical lymph node, skin-draining inguinal lymph node, and mLN, lung and kidney were prepared by mechanical dissociation followed by digestion with DNaseI (0.1 mg ml^−1^) and Collagenase D (0.2 mg ml^−1^) in PBS/HEPES buffer (15 mM HEPES, pH 7.3) for 30 min at 37°C using a magnetic stirrer. LN cells were washed and resuspended in MACS buffer (PBS, 0.5% BSA, 2 mM EDTA, pH 7.3). Bone marrow (BM) was isolated from the femurs and tibias by crushing in MACS buffer. Red blood cells in spleen and BM suspensions were lysed with ammonium-chloride-potassium (ACK) buffer for 90 s at 22°C, after which cells were resuspended in MACS buffer. Lung and kidney suspensions were obtained following a 40% Percoll centrifugation and resuspension in MACS buffer. For B and T cell staining, lymphoid organ suspensions were prepared by mechanical dissociation followed by red blood cell lysis and resuspension in MACS buffer. PP were collected from small intestine and processed as LN. Small intestine and colon were cut into ~1-inch segments and opened longitudinally after removal of fecal content and washed in PBS/HEPES buffer. Intraepithelial cells (IELs) were isolated by incubation in IEL buffer (PBS, 10% FCS, 15 mM HEPES, 5 mM EDTA, pH 7.3) for 20 min at 22°C under stirring. Supernatant containing IEL was washed and lymphocytes collected following a 40% to 70% Percoll gradient centrifugation and resuspended in MACS buffer. SILP and CoLP suspensions were prepared by mechanical dissociation and digestion (SILP/CoLP: HBSS w/o NaHCO_3_, 0.1 mg ml^−1^ DnaseI, 0.2 mg ml^−1^ Collagenase D, pH 7.3; CoLP addition: 1% fatty acid-free BSA) for 50 min at 37°C using a magnetic stirrer, followed by 40% Percoll centrifugation and resuspension in MACS buffer.

### Flow cytometry surface staining

Cells in MACS buffer were incubated with anti-CD16/32 (clone 93) for 10 min, stained by adding primary antibodies for 30 to 45 minutes and, where applicable, washed and incubated with fluorochrome-coupled streptavidin (SAV) for 15 min. All staining steps were performed at 4 °C in the dark. Cells were washed and resuspended in MACS buffer containing 7AAD and acquired using a BD FACSymphony A3 or Cytek Aurora 5L. FlowJo and SpectroFlo were used for data analysis.

### Intracellular flow cytometry staining

Prior to surface staining, cells were washed with PBS and stained with a fixable viability dye for 20 min at 22°C. Following surface staining, cells were fixed and permeabilized according to the manufacturer’s instructions (eBioscience Foxp3 Staining Buffer Set). Intracellular antibody staining was performed for 45 min.

### Cell culture

Cells were re-stimulated for 4 h at 37°C, in complete media (IMDM Glutamax, 10% FBS, 100 U ml^−1^ Penicillin, 100 μg ml^−1^ Streptomycin, 0.1 mM MEM non-essential amino acids solution, 0.05 mM 2-mercaptoethanol) in the presence of 50 ng ml^−1^ PMA, 5 μg ml^−1^ ionomycin, and 5 μg ml^−1^ Brefeldin A. Cytokine production was assessed by intracellular cytokine staining followed by flow cytometry. To induce DC differentiation, 1.5 × 10^6^ BM cells per ml were cultured in complete IMDM in the presence of 100 ng ml^−1^ recombinant hFLT3L for 7 days.

### LEGENDScreen and Infinity Flow

Splenocytes from three *Notch2*^fl/fl^*CD11c*^Cre−^ and three *Notch2*^fl/fl^*CD11c*^Cre+^ mice were prepared as described above. The cell suspensions were stained with CD3-Bio and CD19-Bio and bound with anti-biotin microbeads before negatively purifying the CD3^−^CD19^−^ fraction over an LS column according to manufacturer’s instructions. Cells were stained with a backbone staining including Bst2-PacificBlue, MHC-II-BV510, CD24-BV605, CD172-PE/Dazzle594, Xcr1-BV650, SiglecH-APC, CD11c-APC/Fire750, SAV-PE/Cy7 and CD11b-BV785. Genotypes were distinguished using an antibody mixture containing CD45-PerCP/Cy5.5 for *Notch2*^fl/fl^*CD11c*^Cre−^ and CD45.2-AF700 for *Notch2*^fl/fl^*CD11c*^Cre+^. Following backbone staining, cells were pooled and screened on a BD LSRFortessa for the surface expression of 255 markers using the LEGENDScreen Kit according to the manufacturer’s instructions. We included the DC-specific CD26 (DPP4)^11,75,76^ to distinguish CD26^−^monocyte-derived cells from CD26^+^ cDCs. The resulting data were analyzed with the ‘infinityFlow” package to generate 2D-UMAP projections (ebecht/infinityFlow, GitHub repository)^30,77^. 3D UMAP was generated using ‘umap” from ‘uwot” (n_components=3). Clusters were determined using ‘cytof_cluster” from ‘cytofkit2’ (method=Rphenograph, Rphenograph_k = 30). UMAP visualization was generated using all backbone and expressed InfinityFlow markers, including the genotype-defining CD45 and CD45.2 markers. Clustering analysis was performed after excluding CD45 and CD45.2 to avoid genotype-driven cluster separation. An expression heatmap displaying z-scores of highly expressed and cluster-defining proteins was generated using ‘pheatmap”.

### Bulk RNA-seq

Splenic DCs were prepared and lineage depleted as indicated in the LEGENDScreen method. Lin(CD3, CD19)^−^ cells were sorted into DC3s (CD11c^+^MHC-II^+^XCR1^−^CD16/32^+^ESAM^−^) and cDC1s (CD11c^+^MHC-II^+^XCR1^+^) from *Notch2*^fl/fl^ and *Notch2*^cKO^ mice and cDC2a (CD11c^+^MHC-II^+^XCR1^−^CD16/32^−^ESAM^+^) from *Notch2*^fl/fl^ using a BD FACSymphony S6. Purity (>95%) was confirmed by post-sort analysis. RNA was isolated using the RNeasy Mini Kit with on-column Dnase digestion. Libraries were prepared using the TruSeq Stranded Total RNA Library Prep Kit and sequenced on an Illumina NextSeq 2000 (55 bp paired-end). Reads were quality controlled with ‘FastQC”, aligned to the mm10 genome and quantified using ‘quantMode” from ‘STAR” (v2.7.3a). Genes with less than five reads were excluded. DEGs were identified using DESeq2 (adjusted *P* < 0.05, |log_2_ fold change|> 1) and visualized by volcano plots. Significance is represented as −log_10_(adjusted *P* value). GSEA pathway analysis was performed using ‘fgseaMultilevel” from ‘fgsea” (v1.28.0) with ‘msigdbr” (v7.5.1) Hallmark gene sets.

### Single-cell RNA-seq

#### scRNA-seq1 (lymphoid organs)

The procedure was performed as previously described^22^. Lin(CD3, CD19)^−^ cells were enriched using LS columns as described above. CD11c^+^ cells were sort-purified from spleens, sLNs and mLNs of C57BL/6 or *Notch2*^fl/fl^*CD11c*^Cre+^ using a BD FACSAria II equipped with a custom built-in violet laser. Purity (>95%) was confirmed by post-sort analysis. Cell suspensions were individually multiplexed using Hashtag oligonucleotide (HTO) antibodies. A total of 10 K CD11c^+^ cells from each tissue and genotype were pooled and processed using the Chromium Single Cell 3′ v3 kit (10x Genomics) and Chromium Single Cell Controller for cDNA, gene expression (GEX) and HTO library preparation. Libraries were sequenced using the Illumina NovaSeq 6000 (GEX) and Illumina NextSeq 500 (HTO) platforms as previously described^22^. Data were processed by QC filtering, demultiplexing, alignment to the mm10 genome with Cre sequence inclusion, HTO assignment and annotation of cells and tissue origin, filtering based on QC parameters, clustering analysis and cell-type annotation using Immunological Genome Project (ImmGen) ref.^22^.

#### scRNA-seq2 (colonic)

Cells from mLNs were depleted of Lin (CD3, CD19)^−^ cells using LS columns, and CoLP cells were prepared as described above. Samples were multiplexed with HTO antibodies and pooled before sorting. CD26^+^CD11c^+^MHC-II^+^ cells were sort-purified from eubiotic *Notch2*^fl/fl^, dysbiotic *Notch2*^fl/fl^*, eubiotic rederived *Notch2*^fl/fl^*CD11c*^Cre+^ and dysbiotic *Notch2*^fl/fl^*CD11c*^Cre+^ using a BD FACSymphony S6. Purity (>95%) was confirmed by post-sort analysis. Approximately 60 K mLN DCs and 6 K CoLP DCs were processed using the Chromium Single Cell Controller X and Chromium Single Cell 3′ v4 kit (10x Genomics). GEX and HTO libraries were paired-end sequenced on an Illumina NovaSeq 6000 (configuration: 150-10-10-150; R1-I1-I2-R2). Data were processed as above. Elevated HTO background was addressed by determining the top HTO per cell and applying a minimum UMI threshold together with a dominance ratio relative to the second-highest HTO to ensure confident singlet assignment.

#### Both scRNA-seq datasets

Analyses were performed with Seurat v5 (ref. ^78^) using functions ‘PlotUMAP”, ‘FeaturePlot” and ‘VlnPlot” to plot UMAPs, marker expression and violin plots, respectively. DEGs were identified using”FindMarkers” (test.use=MAST). Plots were generated using ‘ggplot2”, ‘RColorBrewer” and ‘svglite”. Significance in the volcano plots is represented as −log_10_(adjusted *P* value). GSEA was performed using ‘GSEA” from ‘ClusterProfiler”, including all mus muculus Hallmark gene sets (10–500 genes) from ‘msigdbr v7.5.1”. Pathways were visualized using ‘dotplot” from ‘enrichplot” (showCategory=20). Default parameters were used unless stated otherwise. Positive gene correlations were calculated using Spearman’s rank correlation coefficient (⍴) across log-normalized single-cell expression values from each tissue individually. Hallmark pathway enrichment was performed using ‘ClusterProfiler” on positively correlated genes (ρ ≥ 0.2). The background universe consisted of all genes included in the correlation analysis. *P* values were adjusted using the Benjamini–Hochberg method.

### Antibodies for flow cytometry and microscopy

Cells for flow cytometry and tissues for microscopy were stained using the antibodies listed in the Reporting Summary.

### Fecal pellet collection

Fecal samples were collected in a sterile hood using 70% ethanol-disinfected materials and sterile pipette tips.

### Colony-forming units

Fecal pellets were homogenized in PBS. Multiple dilutions were plated onto red blood agar plates, incubated in a 37 °C anaerobic chamber and quantified.

### Shotgun sequencing

Fecal pellets were collected into barcoded tubes containing Transnetyx stabilization buffer. Samples were shipped and processed by Transnetyx. Data were processed and analyzed using the One Codex platform.

### 16S rRNA sequencing

Fecal pellets were collected into sterile 1.5 ml tubes, frozen on dry ice and stored at −80 °C. Frozen samples were homogenized in PM1 solution. The NCI Microbiome and Genetics Core prepared the DNA, amplified and sequenced the V4 region of the 16 rRNA (515F-806R). Demultiplexed paired-end FASTQ files were pre-processed and analyzed in QIIME2 v2.2020.2^79^. α- and β-diversity analyses were performed at a sampling depth of 25803 sequences. Principal-coordinate analysis plots were generated using the Emperor plugin.

### Fecal gavage

Fecal pellets were homogenized in PBS and filtered. The stock concentration was measured using the Nanodrop OD_600_. For high OD gavage, a concentration of OD_600_ = 20, was used. For low-OD gavage, a concentration of OD_600_ = 0.005 was used. Young mice between 6 and 8 weeks of age were gavaged with 100 μl of the homogenized fecal samples.

### Bacterial flow cytometry

Blood and fecal pellets were collected and prepared as described above. Bacterial flow cytometry^80^ was performed on either individual or pooled samples. Fecal suspensions were adjusted to OD_600_ = 1 in bacterial staining buffer (BSB; PBS, 1% BSA, 0.025% sodium azide), mixed 1:1 with BSB containing mouse serum (1:50), and incubated o/n at 4 °C. Samples were washed and stained with either anti-mouse IgA-Bio or IgG2c-Bio for 60 min, followed by SAV-PE/Cy7 for 30 min. After washing, samples were resuspended in SYBR-Green (1:10,000, in BSB) for 1 h and acquired on a BD FACSymphony A3.

For flow cytometry on *Akkermansia muciniphila* (BAA-835), bacteria were grown in Brain Heart Infusion broth (BHI, ATCC Medium 44) containing L-cysteine (0.5 g liter^−1^) under anaerobic conditions at 37 °C for 4 to 7 days. OD_600_ was adjusted to 1 and stained with serum as described above.

For flow cytometry on *Mucispirillum ASF457*, bacteria were grown in Anaerobic Mucispirillum Medium (AMM)^67^ under anerobic conditions at 37 °C for 4 to 7 days. OD_600_ was adjusted to 1 and stained with serum as described above. One liter of AMM contains 18 g brain heart broth, 15 g tryptone soy broth, 5 g yeast extract, 2.5 g K_2_HPO_4_, 1 mg hemim, 0.5 mg vitamin K1, 0.4 g Na_2_CO_3_, 1.01 g KNO_3_, 3% fetal calf serum and 0.5 mg L-cysteine hydrochloride.

### Statistical analysis

Statistical analyses were performed using GraphPad Prism (GraphPad Software). Differences between groups were assessed by unpaired or paired two-tailed Student’s *t* test, two-sided Mann-Whitney U tests, as appropriate. Survival curves were determined by log-rank Mantel-Cox test. *P* values less than 0.05 were considered significant (*P < 0.05, **P < 0.01, ***P < 0.001, ****P < 0.0001). Where data did not meet the assumption of normality, nonparametric tests were used. For datasets in which normality was not formally assessed, data were assumed to follow normal distribution.

### Reporting summary

Further information on research design is available in the Nature Portfolio Reporting Summary linked to this article.

## Online content

Any methods, additional references, Nature Portfolio reporting summaries, source data, extended data, supplementary information, acknowledgements, peer review information; details of author contributions and competing interests; and statements of data and code availability are available at 10.1038/s41590-026-02599-z.

## Supplementary information

Supplementary Information Supplementary Figs. 1–5.

Reporting Summary

Peer Review File

Supplementary Table 1 List of DEGs from bulk RNA-seq analysis between DC3, cDC2a and cDC1 of *Notch2*^fl/fl^ and *Notch2*^cKO^, as depicted in the volcano plots in Fig. 1 and associated figures.

Supplementary Table 2 Top 15 expressed genes per cluster from WT and *Notch2*^cKO^, as depicted in the heatmap in Fig. 2.

Supplementary Table 3 List of DEGs from scRNA-seq analysis between WT and *Notch2*^cKO^ clusters, corresponding to Fig. 2 and associated figure volcano plots.

Supplementary Table 4 List of microbes across all taxonomic ranks identified by shotgun sequencing of fecal material from *Notch2*^fl/fl^ and *Notch2*^cKO^ mice, related to Fig. 4 and associated figure.

Supplementary Table 5 Top 15 expressed genes per cluster from all tissues (mLN and CoLP) and genotypes (*Notch2*^fl/fl^, rederived *Notch2*^cKO^, *Notch2*^fl/fl^* co-housed with *Notch2*^cKO^, *Notch2*^cKO^) combined, as shown in the Extended Data Fig. 9 heatmap.

Supplementary Table 6 List of DEGs from scRNA-seq analysis between *Notch2*^fl/fl^, rederived *Notch2*^cKO^, *Notch2*^fl/fl^* co-housed with *Notch2*^cKO^ and *Notch2*^cKO^ cluster 9, corresponding to Fig. 8 and associated figure volcano plots.

## Source data

Source Data All Figures All source data.

## Data Availability

**Accession numbers**: All sequencing datasets were deposited under the umbrella BioProject PRJNA1265721 at the NCBI Sequence Read Archive (SRA). The *Notch2*^cKO^ scRNA-seq dataset is available at the NCBI Gene Expression Omnibus (GEO) under accession number GSE297642 (Fig. 2) and GSE326772 (Fig. 8). The C57BL/6 samples were previously submitted to GEO under the accession number GSE223841. The bulk RNA-seq dataset is available under the accession number GSE297493. The steady state metagenomic shotgun sequencing data can be found under the accession number PRJNA1264487. The 16S rRNA sequencing data from the co-housing study are available under the accession number PRJNA1264490, and those from the antibiotic treatment study are available under the accession number PRJNA1264494. **Interactive scRNA-seq applications**: Interactive Shiny applications for visualization of the scRNA-seq datasets are available at https://tuss-rox-lab.shinyapps.io/shinyapp/ (Fig. 2) and https://tuss-rox-lab.shinyapps.io/shinyapp-1/ (Fig. 8). Source data are provided with this paper.
