# Supplementary material for: Dysbiosis-induced expansion of AXL-positive inflammatory type 3 dendritic cells triggers preclinical autoimmunity
Source: Nat Immunol. 2026 Jul 21;27(9):1856–73. doi: 10.1038/s41590-026-02599-z (PMC13506347; doi:10.1038/s41590-026-02599-z)
Supplement: Supplementary file 1 [file 41590_2026_2599_MOESM1_ESM.pdf]

nature immunology

Supplementary information

https://doi.org/10.1038/s41590-026-02599-z

Dysbiosis-induced expansion of AXL-positive inflammatory type 3 dendritic cells triggers preclinical autoimmunity

In the format provided by the authors and unedited

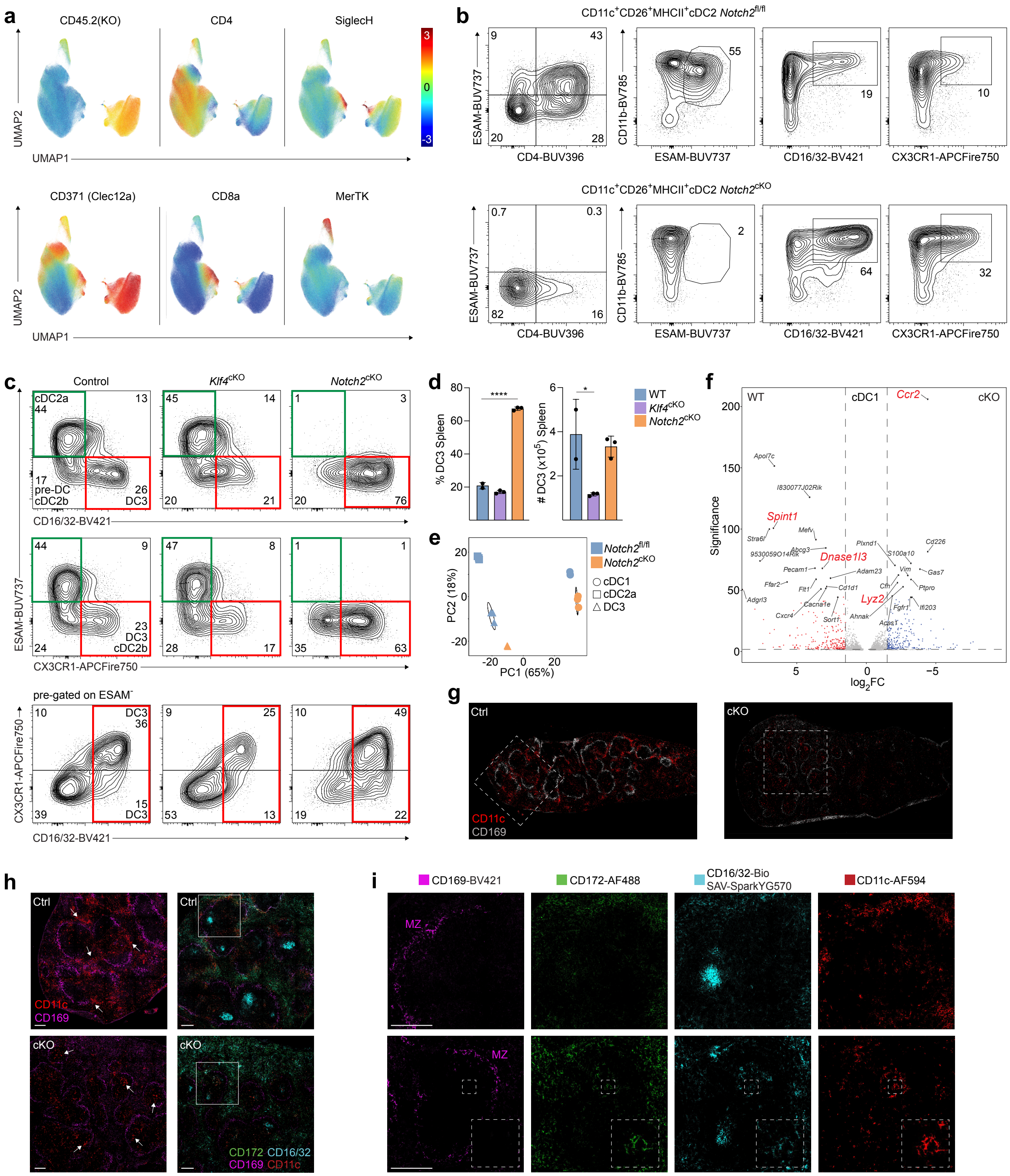

Supplementary Fig. 1. *Notch2* deficiency alters all DC subsets. Related to Fig. 1. (**a**) Cells were prepared as described in Fig. 1a. UMAP showing relative expression levels of the indicated markers within

cDC2s. (**b**) Flow cytometry plots of co-expression profiles on splenic cDC2 from *Notch2*$^{fl/fl}$ and *Notch2*^{cKO} using the indicated markers. Representative plots of three independent experiments (*n* = 3 mice). (**c-d**) Analysis of splenic DC subsets (CD3$^-$CD19$^-$CD11c$^+$MHCII$^+$CD26$^+$) in C57BL/6, *Klf4*^{cKO}, and *Notch2*^{cKO}. Flow cytometry plots (**c**) and frequencies (**d**) of splenic DC3 (XCR1$^-$ESAM$^-$CX3CR1$^+$CD16/32$^+$). (*n* = 2, 3 and 3 mice, one representative experiment). Data are shown as mean +/- SD. Statistical analysis was performed using an unpaired two-tailed *t*-test. *P>0.05, **P>0.01, ****P>0.0001. (**e-f**) Bulk RNA-seq was performed as described in Fig. 1g. (**e**) PCA of the sorted subsets (blue symbols: *Notch2*$^{fl/fl}$; orange symbols: *Notch2*^{cKO}). (**f**) Volcano plots showing the top 30 DEGs between *Notch2*$^{fl/fl}$ and *Notch2*^{cKO} cDC1. Significance is represented as -$\log_{10}$(adjusted *P* value). (**g-i**) Confocal fluorescence images of splenic DC populations in *Notch2*$^{fl/fl}$ and *Notch2*^{cKO}. Related to Fig. 1h. Areas highlighted in panel **g** are shown at higher magnification in panels **h-i**. The indicated marker combinations (**g-h**) or individual markers (**i**) are shown: CD169 (white or magenta), CD11c (red), CD172 (green), CD16/32 (cyan). Scale bar: 150mm. Arrows indicate differential CD11c localization. Dashed squares denote magnified regions. The images are representative of two independent experiments (each *n* = 3 mice).

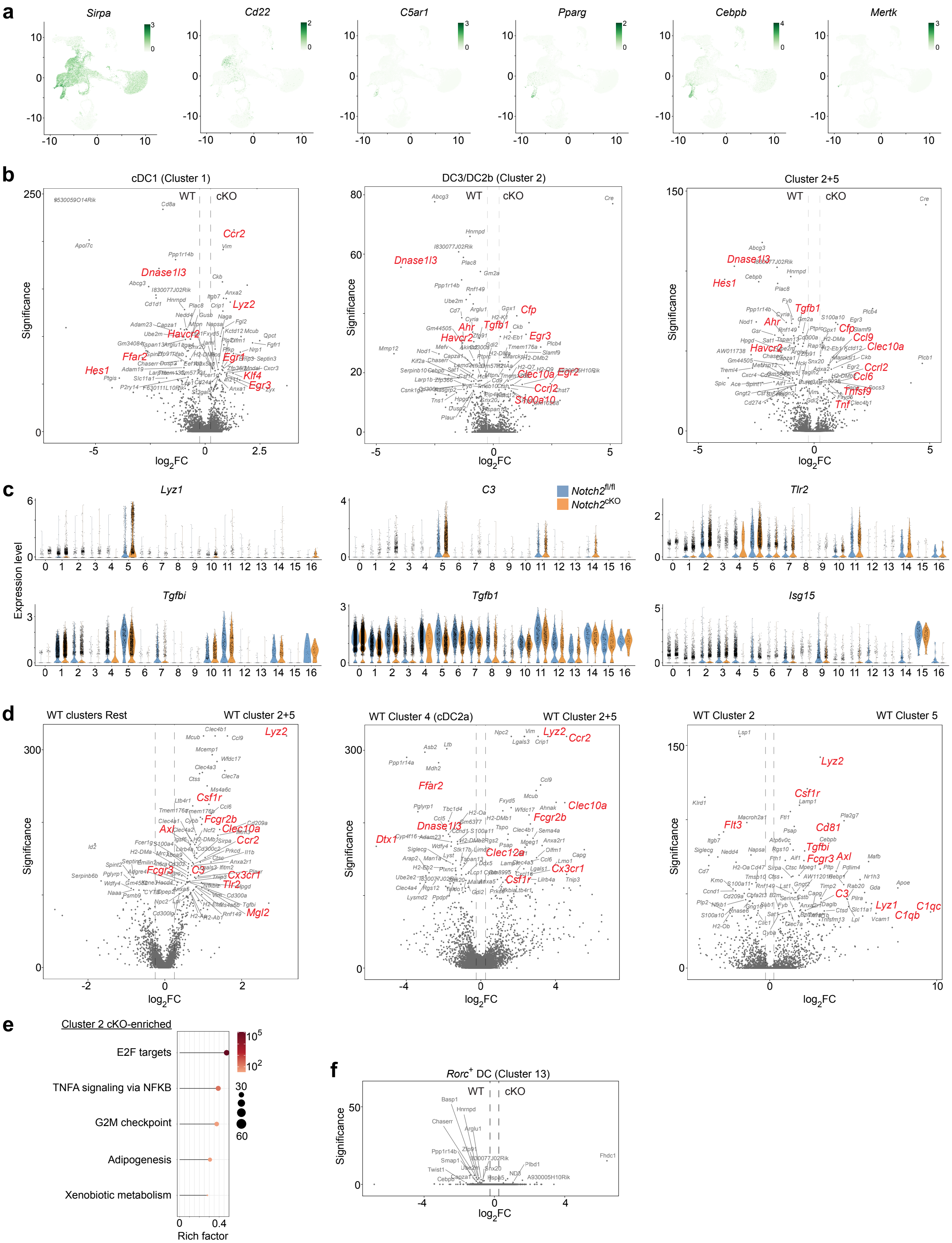

a
Sirpa
Cd22
C5ar1
Pparg
Cebpb
Mertk
b
cDC1 (Cluster 1)
DC3/DC2b (Cluster 2)
Cluster 2+5
WT
cKO
Significance
log2FC
Ccr2
Dnase1l3
Lyz2
Havcr2
Ffar2
Hes1
Egr3
Klf4
Egr3
Ahr
Tgfb1
Cfp
Clec10a
Egr2
Ccrl2
S100a10
Ccl9
Ccl6
Tnfsf9
Tnf
c
Lyz1
C3
Tlr2
Tgfbi
Tgfb1
Isg15
Notch2fl/fl
Notch2cKO
Expression level
d
WT clusters Rest
WT cluster 2+5
WT Cluster 4 (cDC2a)
WT Cluster 2
WT Cluster 5
Csf1r
Fcgr2b
Axl
Fcgr3
C3
Cx3cr1
Tlr2
Mgl2
Dtx1
Clec12a
Flt3
Cd81
Tgfbi
Lyz1
C1qc
C1qb
e
Cluster 2 cKO-enriched
E2F targets
TNFA signaling via NFKB
G2M checkpoint
Adipogenesis
Xenobiotic metabolism
Rich factor
f
Rorc+ DC (Cluster 13)

Supplementary Fig. 2. Pro-inflammatory AXL^{+}inf-DC3 systemically expand in *Notch2*^{cKO} mice. Related to Fig. 2. (**a-f**) Cells were prepared as described in Fig. 2a. (**a**) UMAP showing relative expression levels of the indicated genes including cells from each genotype and tissue. (**b**) Volcano plots showing the top 80 DEGs between WT and *Notch2*^{cKO} cluster 1 (cDC1) (left), top 40 DEGs between WT and *Notch2*^{cKO} cluster 2 (DC2b/DC3) (middle), top 80 DEGs between WT and *Notch2*^{cKO} cluster 2+5 (cDC2b/DC3/inf-DC3) (right). Significance is represented as -$\log_{10}$(adjusted *P* value). (**c**) Violin plots showing log-normalized gene expression levels for the indicated genes across all clusters in WT (blue) and *Notch2*^{cKO} (orange). (**d**) Volcano plot showing the top 80 DEGs between WT Rest (all clusters except 2 and 5) and WT clusters 2+5 (cDC2b/DC3/inf-DC3) (left), WT cluster 4 (cDC2a) and clusters 2+5 (cDC2b/DC3/inf-DC3) (middle), WT cluster 2 (cDC2b/DC3) and cluster 5 (inf-DC3) (right). Significance is represented as -$\log_{10}$(adjusted *P* value). (**e**) GSEA pathway analysis was performed on DEGs of cluster 2 (cDC2b/DC3) WT vs cKO. The cKO-enriched pathways are displayed as dot plot. GSEA was performed using the Molecular Signatures Database [MSigDB (V7.5.1)]. Dot size represents the number of core enriched genes. Color indicates the adjusted *P* value. The rich factor represents the ratio of core enriched genes to the total number of genes in the gene set. (**f**) Volcano plot showing the top DEGs between WT and *Notch2*^{cKO} cluster 13 (*Rorc*$^{+}$ DC). Significance is represented as -$\log_{10}$(adjusted *P* value).

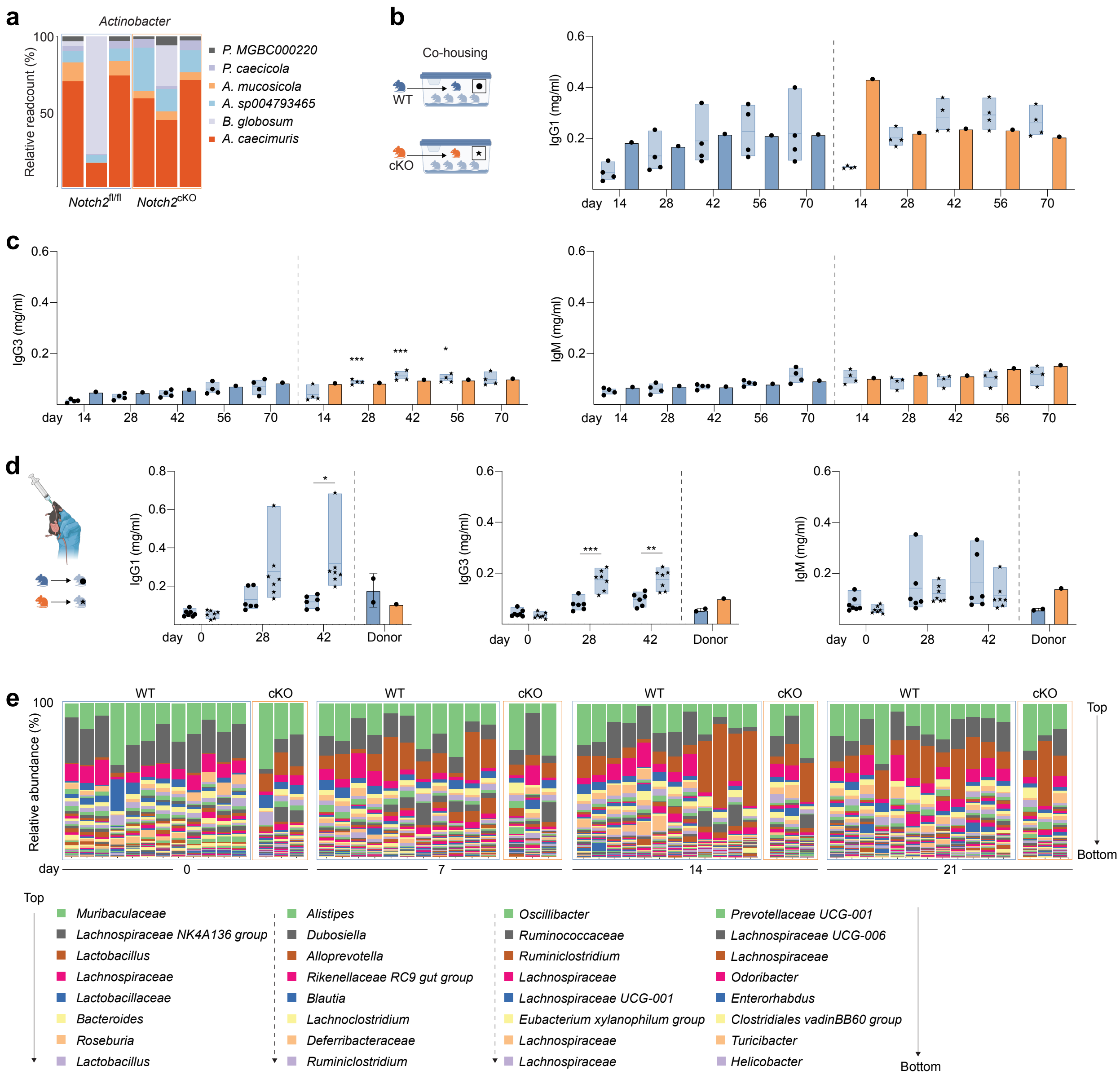

Supplementary Fig. 3. *Notch2*^{cKO} mice have intestinal dysbiosis that can be transferred. Related to Fig. 4. (**a**) Shotgun sequencing was performed as described in Fig. 4b. (*n* = 3 mice, representative images of two independent experiments are shown). Shown is the quantification of bacterial species within the *Actinobacteria* phylum in *Notch2*$^{fl/fl}$ (blue square) and *Notch2*^{cKO} (orange square) mice. (**b-c**) Co-housing was performed as described in Fig. 4g. Serum immunoglobulin concentrations for each time-point were determined by ELISA. C57BL/6 co-housed with *Notch2*$^{fl/fl}$ (light blue, circle symbol), control *Notch2*$^{fl/fl}$ (dark blue), C57BL/6* co-housed with *Notch2*^{cKO} (light blue), and control *Notch2*^{cKO} (orange). Statistical analysis

was performed between the WT groups. (recipient *n* = 4 mice per experiment, donor: *n* = 1 per experiment, representative data of three independent experiments). (**d**) Oral gavage was performed as described in Fig. 4h. Shown are the schematic representation of the experimental design and serum immunoglobulin concentrations at each time-point, determined by ELISA, in C57BL/6 gavaged with *Notch2*$^{fl/fl}$ fecal material (light blue, circle symbol), C57BL/6* gavaged with *Notch2*^{cKO} fecal material (light blue, star symbol), *Notch2*$^{fl/fl}$ (dark blue), and *Notch2*^{cKO} (orange). (recipient *n* = 6 and 7 mice, donor: *n* = 2 mice and 1 mouse, pooled data from two independent experiments). (**e**) The experimental setup for 16S rRNA sequencing of fecal samples from co-housed mice is outlines in Fig. 4i. Shown is the relative abundance of indicated bacteria at their lowest taxonomic rank (top to bottom) in C57BL/6 (blue square) and *Notch2*^{cKO} (orange square). (C57BL/6: *n* = 12 mice, *Notch2*^{cKO}: *n* = 3 mice, representative data from two independent experiments are shown). Data are shown as mean +/- SD. Statistical analysis was performed using an unpaired two-tailed *t*-test. *P>0.05, **P>0.01, ***P>0.001, ****P>0.0001

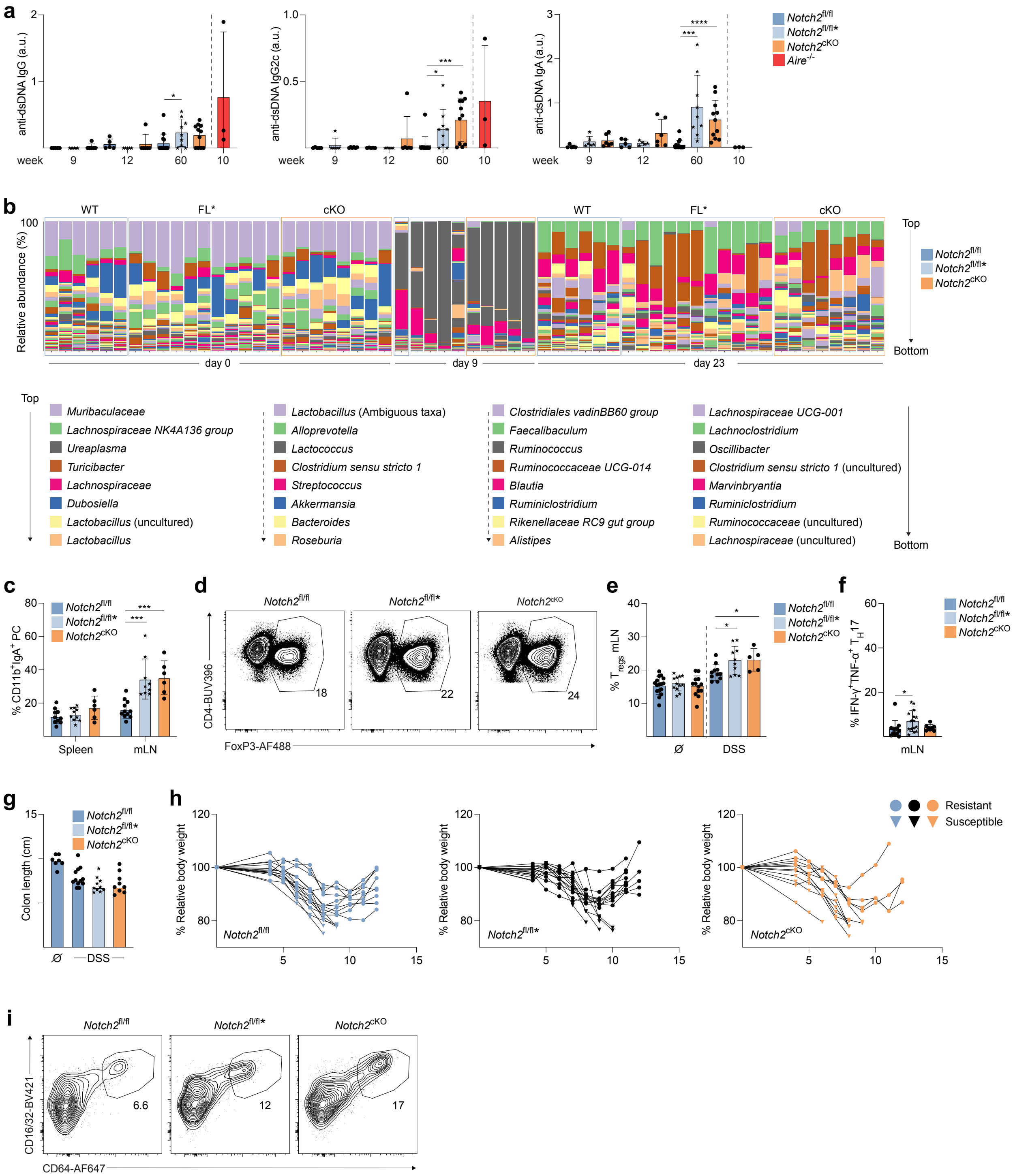

Supplementary Fig. 4. Colonization by three pathobionts is sufficient to drive autoimmune features. Related to Fig. 6. (**a**) Serum anti-dsDNA specific IgG (left), IgG2c (middle), and IgA (right) antibody

concentrations in *Notch2*$^{fl/fl}$ (dark blue), *Notch2*$^{fl/fl}$* co-housed with *Notch2*^{cKO} (light blue), *Notch2*^{cKO} (orange), and autoimmune reference *Aire*$^{-/-}$ (red) mice at the indicated age in weeks were measured by ELISA. Data are presented as arbitrary units (a.u.). (9wks (groups): *n* = 5, 6, and 6 mice, 12wks (groups): *n* = 5, 5, and 6 mice, 60wks (groups): *n* = 14, 9, and 11 mice, *Aire*$^{-/-}$: *n* = 3, pooled data from two independent experiments). (**b**) Antibiotic treatment was performed as described in Fig. 6b on 15-week-old *Notch2*$^{fl/fl}$ (dark blue), *Notch2*$^{fl/fl}$* co-housed with *Notch2*^{cKO} (light blue), and *Notch2*^{cKO} (orange). 16S rRNA sequencing was performed on the fecal material collected for 23 days during the antibiotic treatment study. Shown is the relative abundance of the indicated bacteria at their lowest taxonomic rank (top to bottom). (Groups: *n* = 6, 11, and 8 mice, pooled data from two independent experiments). (**c-i**) The DSS-colitis study was performed as described in Fig. 6h on 25–30-week-old *Notch2*$^{fl/fl}$ (blue), *Notch2*$^{fl/fl}$* co-housed with *Notch2*^{cKO} (light blue), and *Notch2*^{cKO} (orange) mice. (**c**) Frequency of CD11b expression within CD138$^+$IgA$^+$ PC was determined by flow cytometry for the indicated genotypes and tissues in DSS treated mice. (Spleen: *n* = 10, 10, and 6 mice, mLN: n = 11, 9, and 6 mice, pooled data from two to three independent experiments). (**d-e**) Analysis of T_{reg} and T_H17 populations in mice of the indicated genotype without (Ø) or with DSS treatment. (**d**) Representative flow cytometry plots for DSS treated splenic FoxP3$^+$ T_{reg}, as pre-gated in 3j. (**e**) Frequencies of FoxP3$^+$ T_{reg} among CD4$^+$ T cells in the mLN. (Sample sizes are indicated from left to right. *n* = 16, 12, 13, 11, 10, and 5 mice, pooled data from three independent experiments). (**f**) Frequencies of pathogenic TNF-a$^+$IFN-g$^+$ expressing IL-17$^+$$T_H17$ (RORgt$^+$CD4$^+$ T cells, pre-gated as in 3j) within the mLN of DSS treated mice of the indicated genotypes were determined by flow cytometry after PMA and ionomycin stimulation. (Groups: *n* = 12, 15, and 8 mice, pooled data from three independent experiments). (**g**) Quantification of colon length from each genotype. (Groups: *n* = 7, 12, 10 and 9 mice, pooled data from three independent experiments). (**h**) Relative body weight curves are shown for each genotype over the course of the DSS treatment (Groups: *n* = 16, 16, and 12 mice, pooled data from three independent experiments). (**i**) DC populations were analyzed by flow cytometry in mice, without (Ø) or with DSS treatment. Shown are representative flow cytometry plots of CD64$^+$AXL$^+$inf-DC3 among DC2/DC3, pre-gated as in Extended Data Fig. 2g, from the mLN of DSS treated mice of each genotype. Data are shown as mean +/- SD. Statistical analysis was performed using an unpaired two-tailed *t*-test for comparisons between independent groups, or a paired two-tailed *t*-test for repeated measures involving the same mice across multiple time-points. *P>0.05, **P>0.01, ***P>0.001, ****P>0.0001

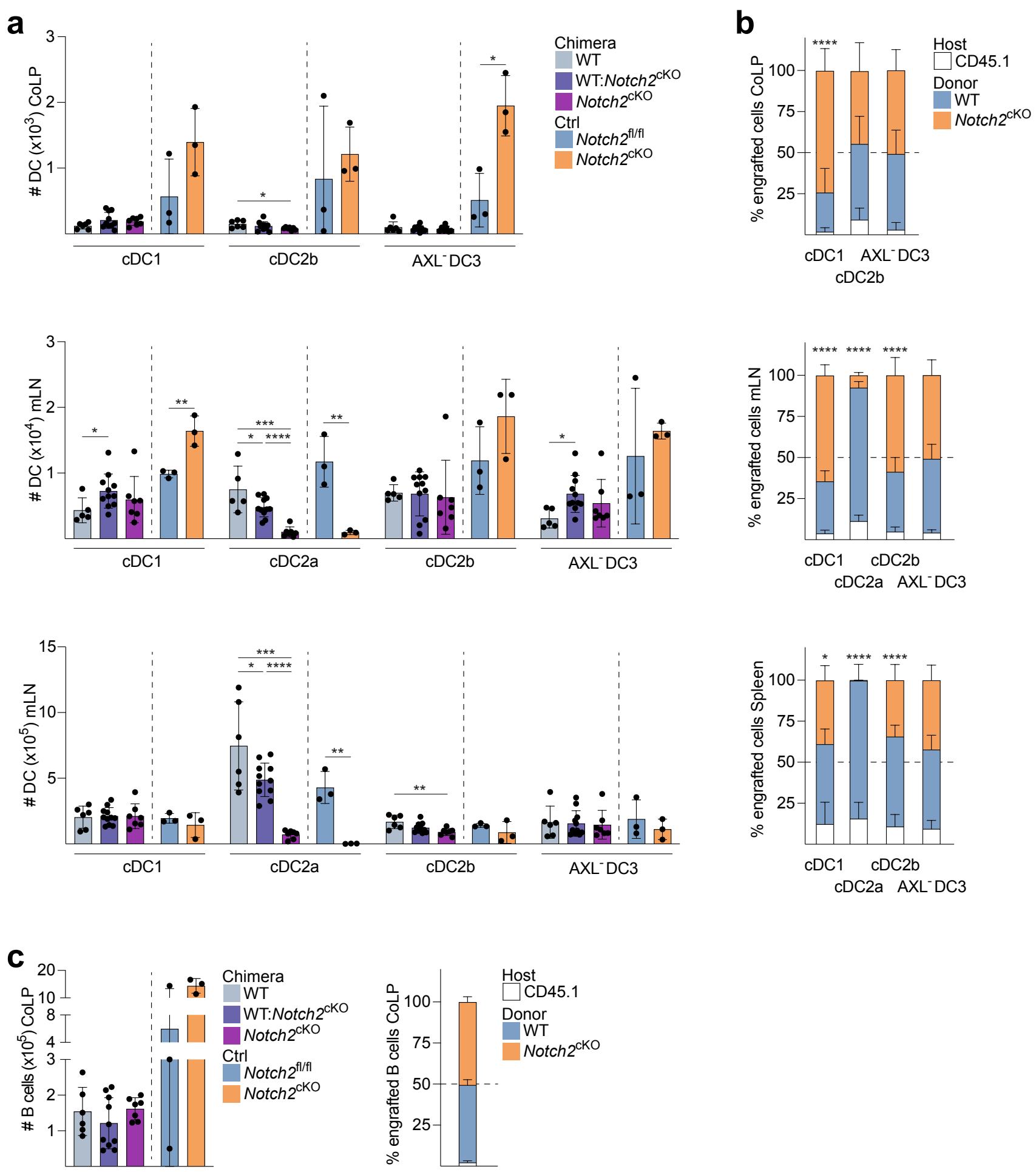

Supplementary Fig. 5. AXL$^+$inf-DC3 respond to intestinal dysbiosis. Related to Fig. 7. (**a-c**) BM chimeras were generated as described in Fig. 7k. Total donor cell numbers and frequencies of donor-derived DC subsets (**a-b**), pre-gated as in Extended Data Fig. 2g, and CD19$^+$B220$^+$B cells (**c**) in the indicated tissues are shown and compared with steady-state control mice. (Sample sizes are indicated from left to right. CoLP: *n* = 6, 10, 7, 3, and 3 mice, mLN: *n* = 5, 11, 7, 3, and 3 mice, spleen: *n* = 6, 11, 7, 3, and 3 mice, pooled data from two independent experiments). Data are shown as mean +/- SD. Statistical analysis was performed using an unpaired two-tailed *t*-test. *P>0.05, **P>0.01, ***P>0.001, ****P>0.0001
