# Supplementary material for: Dysbiosis-induced expansion of AXL-positive inflammatory type 3 dendritic cells triggers preclinical autoimmunity
Source: Nat Immunol. 2026 Jul 21;27(9):1856–73. doi: 10.1038/s41590-026-02599-z (PMC13506347; doi:10.1038/s41590-026-02599-z)
Supplement: Supplementary file 2 [file 41590_2026_2599_MOESM2_ESM.pdf]

nature portfolio

Corresponding author(s): Roxane Tussiwand

Last updated by author(s): Jun 10, 2026

Reporting Summary

Nature Portfolio wishes to improve the reproducibility of the work that we publish. This form provides structure for consistency and transparency in reporting. For further information on Nature Portfolio policies, see our Editorial Policies and the Editorial Policy Checklist.

Statistics

For all statistical analyses, confirm that the following items are present in the figure legend, table legend, main text, or Methods section.

n/a	Confirmed	
☐	☒	The exact sample size (*n*) for each experimental group/condition, given as a discrete number and unit of measurement
☐	☒	A statement on whether measurements were taken from distinct samples or whether the same sample was measured repeatedly
☐	☒	The statistical test(s) used AND whether they are one- or two-sided<br>*Only common tests should be described solely by name; describe more complex techniques in the Methods section.*
☒	☐	A description of all covariates tested
☒	☐	A description of any assumptions or corrections, such as tests of normality and adjustment for multiple comparisons
☐	☒	A full description of the statistical parameters including central tendency (e.g. means) or other basic estimates (e.g. regression coefficient) AND variation (e.g. standard deviation) or associated estimates of uncertainty (e.g. confidence intervals)
☐	☒	For null hypothesis testing, the test statistic (e.g. F, t, r) with confidence intervals, effect sizes, degrees of freedom and P value noted<br>*Give P values as exact values whenever suitable.*
☒	☐	For Bayesian analysis, information on the choice of priors and Markov chain Monte Carlo settings
☒	☐	For hierarchical and complex designs, identification of the appropriate level for tests and full reporting of outcomes
☒	☐	Estimates of effect sizes (e.g. Cohen's *d*, Pearson's *r*), indicating how they were calculated

Our web collection on statistics for biologists contains articles on many of the points above.

Software and code

Policy information about availability of computer code

Data collection	Flow cytometry and cell sorting: Cells were acquired using a BD LSRFortessa, BD FACSymphony A3 or Cytek Aurora 5L. Cells were sorted using a BD FACSAria II with a custom built-in violet laser or BD FACSymphony S6 Sorter.<br>Bulk RNA-seq: Libraries were sequenced using an Illumina NextSeq 2000.<br>scRNA-seq Fig2: Single cells were captured using a 10x Genomics Chromium Single Cell Controller. GEX and HTO libraries were sequenced using the Illumina NovaSeq 6000 and Illumina NextSeq 500, respectively.<br>scRNA-seq Fig8: Single cells were captured using a 10x Genomics Chromium Single Cell Controller X. GEX and HTO libraries were sequenced using the Illumina NovaSeq 6000.<br>Microscopy: Images were acquired using a Leica SP8 confocal equipped with a white light laser using a 20X (N.A. 0.8) objective and controlled by LASX software or acquired using a widefield Nikon TiE equipped with an 8-line Spectra Light engine, a Hamamatsu BT fusion camera and a Sutter Instruments filter wheel. H&E images were acquired with the S60 NanoZoomer Digital Scanner by Hamamatsu.<br>ELISA: The plates were read using a SpectraMaxPlus 384 (Molecular Devices)
Data analysis	Flow cytometry: FlowJo (v10, Tree Star) or SpectroFlo (v2.2.0.4, Cytek Biosciences) .<br>Graphs and statistics: GraphPad Prism (v10, Dotmatix)<br>Sequencing: R (4.3.2, The R Foundation), RStudio (v2023.12.1+402, Posit PBC), and analysis packages as described in the manuscript.<br>Microscopy: ImageJ2 (v2.9.0, FIJI (NIH and LOCI)) or NDP.view2 (Hamamatsu Photonics)<br>Microbiome: OneCodex (Minot et al, 2015) and qiime2 (Caporaso Lab, Boyden et al. 2019).

For manuscripts utilizing custom algorithms or software that are central to the research but not yet described in published literature, software must be made available to editors and reviewers. We strongly encourage code deposition in a community repository (e.g. GitHub). See the Nature Portfolio guidelines for submitting code & software for further information.

Data

Policy information about availability of data

All manuscripts must include a data availability statement. This statement should provide the following information, where applicable:

- Accession codes, unique identifiers, or web links for publicly available datasets
- A description of any restrictions on data availability
- For clinical datasets or third party data, please ensure that the statement adheres to our policy

The Umbrella BioProject accession number PRJNA1265721 links all the following datasets, each of which can be accessed using its unique identifier. The Notch2cKO scRNA-seq dataset is available at the NCBI Gene Expression Omnibus (GEO) under accession number GSE297642 (Fig. 2) and GSE326772 (Fig. 8). The C57BL/6 samples were previously submitted to GEO under the accession number GSE223841. The bulk RNA-seq dataset is available under the accession number GSE297493. The steady state metagenomic shotgun sequencing data can be found under the accession number PRJNA1264487. The 16S rRNA sequencing data from the co-housing study are available under the accession number PRJNA1264490, and those from the antibiotic treatment study under the accession number PRJNA1264494.

Research involving human participants, their data, or biological material

Policy information about studies with human participants or human data. See also policy information about sex, gender (identity/presentation), and sexual orientation and race, ethnicity and racism.

Reporting on sex and gender	*Use the terms sex (biological attribute) and gender (shaped by social and cultural circumstances) carefully in order to avoid confusing both terms. Indicate if findings apply to only one sex or gender; describe whether sex and gender were considered in study design; whether sex and/or gender was determined based on self-reporting or assigned and methods used. Provide in the source data disaggregated sex and gender data, where this information has been collected, and if consent has been obtained for sharing of individual-level data; provide overall numbers in this Reporting Summary. Please state if this information has not been collected. Report sex- and gender-based analyses where performed, justify reasons for lack of sex- and gender-based analysis.*
Reporting on race, ethnicity, or other socially relevant groupings	*Please specify the socially constructed or socially relevant categorization variable(s) used in your manuscript and explain why they were used. Please note that such variables should not be used as proxies for other socially constructed/relevant variables (for example, race or ethnicity should not be used as a proxy for socioeconomic status). Provide clear definitions of the relevant terms used, how they were provided (by the participants/respondents, the researchers, or third parties), and the method(s) used to classify people into the different categories (e.g. self-report, census or administrative data, social media data, etc.) Please provide details about how you controlled for confounding variables in your analyses.*
Population characteristics	*Describe the covariate-relevant population characteristics of the human research participants (e.g. age, genotypic information, past and current diagnosis and treatment categories). If you filled out the behavioural & social sciences study design questions and have nothing to add here, write "See above."*
Recruitment	*Describe how participants were recruited. Outline any potential self-selection bias or other biases that may be present and how these are likely to impact results.*
Ethics oversight	*Identify the organization(s) that approved the study protocol.*

Note that full information on the approval of the study protocol must also be provided in the manuscript.

Field-specific reporting

Please select the one below that is the best fit for your research. If you are not sure, read the appropriate sections before making your selection.

☒ Life sciences ☐ Behavioural & social sciences ☐ Ecological, evolutionary & environmental sciences

For a reference copy of the document with all sections, see nature.com/documents/nr-reporting-summary-flat.pdf

Life sciences study design

All studies must disclose on these points even when the disclosure is negative.

Sample size	Sample sizes for all experiments are provided in the figure legends. No statistical methods were used to pre-determine sample; however, pilot studies were used to estimate the number of animals required to ensure adequate power. At least three biological replicates were used unless otherwise specified.
Data exclusions	Data were excluded only if tissue processing resulted in non-viable samples.
Replication	All experiments were repeated and reliably reproduced. The number of repetitions is indicated in the figure legends. Most plots show pooled data from all experimental repetitions.

Randomization: The experimental groups were defined based on their genotype. Age- and sex-matched mice were used as controls and littermates were included when applicable, as indicated in the manuscript. Mice exhibiting severe barbering were not used for the study.

Blinding: Blinding was not performed during data collection and analysis, as the outcomes were non-subjective.

Reporting for specific materials, systems and methods

We require information from authors about some types of materials, experimental systems and methods used in many studies. Here, indicate whether each material, system or method listed is relevant to your study. If you are not sure if a list item applies to your research, read the appropriate section before selecting a response.

Materials & experimental systems

n/a	Involved in the study	
☐	☒	Antibodies
☒	☐	Eukaryotic cell lines
☒	☐	Palaeontology and archaeology
☐	☒	Animals and other organisms
☒	☐	Clinical data
☒	☐	Dual use research of concern
☒	☐	Plants

Methods

n/a	Involved in the study	
☒	☐	ChIP-seq
☐	☒	Flow cytometry
☒	☐	MRI-based neuroimaging

Antibodies

Antibodies used: Fluorochrome-coupled antibodies for flow cytometry (all) and also used for microscopy (M) are listed below

Antibody Fluorophor Clone Company Catalog Nr. Dilution
Live/Dead 7AAD Biolegend 420403 1:400
Axl AF488 (M) MAXL8DS eBioscience 53-1084-82 1:800
Axl APC MAXL8DS eBioscience 17-1084-80 1:800
B220 Biotin RA3-6B2 Biolegend 103204 1:400
B220 BUV563 RA3-6B2 BD 748868 1:200
CD103 Biotin 2E7 eBioscience 13-1031-85 1:400
CD103 BV711 2E7 Biolegend 121435 1:400
CD105 Biotin MJ7/18 Biolegend 120404 1:200
CD11b Biotin M1/70 Biolegend 101204 1:400
CD11b PerCP-Cy5.5 M1/70 Biolegend 101228 1:800
CD11b BV785 M1/70 Biolegend 101243 1:800
CD11c Purified (M) N418 Biolegend 117302 1:400
CD11c Biotin (M) N418 Biolegend 117304 1:400
CD11c PE/Dazzle594 N418 Biolegend 117348 1:400
CD11c BV711 N418 Biolegend 117349 1:400
CD11c APC Fire 750 N418 Biolegend 117352 1:400
CD115 BV711 AF298 Biolegend 135515 1:800
CD117 APC/Fire750 2B8 Biolegend 105838 1:200
CD135 PE A2F10 Biolegend 135306 1:200
CD135 APC A2F10 Biolegend 135310 1:200
CD138 PE 281-2 Biolegend 142504 1:400
CD16/32 Purified 93 Biolegend 101302 1:200
CD16/32 Biotin (M) 93 Biolegend 101303 1:200
CD16/32 BV421 (M) 93 Biolegend 101332 1:200
CD16/32 BV711 93 Biolegend 101337 1:200
CD16/32 APC/Cy7 93 Biolegend 101328 1:400
CD169 (M) BV421 3D6.112 Biolegend 142421 1:400
CD172a AF488 P84 Biolegend 144024 1:400
CD172a PE/Dazzle594 P84 Biolegend 144016 1:400
CD172a PerCP-eFluor710 P84 eBioscience 46-1721-82 1:200
CD172a AF594 P84 Biolegend 144020 1:400
CD19 Biotin 6D5 Biolegend 115504 1:400
CD19 BV510 (M) 6D5 Biolegend 115546 1:400
CD19 Spark YG570 (M) 6D5 Biolegend 115580 1:400
CD19 BUV805 1D3 BD 568287 1:400
CD21 PeDazzle594 7E9 Biolegend 123440 1:400
CD23 PE/Cy7 B3B4 Biolegend 110614 1:400
CD24 BV605 M1/69 Biolegend 101827 1:800
CD26 PE H194-112 Biolegend 137803 1:200
CD26 PE/Cy7 H194-112 Biolegend 137810 1:200
CD279 BV711 29F.1A12 Biolegend 135231 1:400
CD3 Biotin 17A2 Biolegend 100244 1:400

CD3 FITC (M) 17A2 Biolegend 100204 1:400
CD3 PE/Fire700 17A2 Biolegend 100272 1:200
CD3 PE/Cy7 17A2 Biolegend 100320 1:400
CD317 Pacific Blue 927 Biolegend 127018 1:200
CD317 BV786 927 BD 747603 1:500
CD38 AF700 90 eBioscience 56-0381-82 1:400
CD4 Biotin (M) GK.15 Biolegend 100404 1:400
CD4 BUV395 RM4-4 BD 740209 1:200
CD4 BUV395 RM4-5 BD 568375 1:200
CD4 BV421 (M) RM4-5 Biolegend 100544 1:400
CD4 BUV661 RM4-4 BD 741462 1:400
CD45 PerCP-Cy5.5 30-F11 Biolegend 103132 1:400
CD45.2 AF700 104 Biolegend 109822 1:200
CD64 AF647 X54-5/7.1 Biolegend 139321 1:200
CD8 Biotin (M) 53-6.7 Biolegend 100704 1:400
CD8 BUV496 53-6.7 BD 750024 1:200
CD8 FITC (M) 53-6.7 Biolegend 100706 1:400
CD8 BV650 53-6.7 Biolegend 100742 1:800
CD8 BV785 53-6.7 Biolegend 100750 1:400
CD86 BV650 GL-1 Biolegend 105036 1:400
CD90.2 BV421 30-H12 Biolegend 105341 1:1000
CD90.2 AF700 53-2.1 Biolegend 140324 1:500
CD95 BV605 SA367H8 Biolegend 152612 1:400
CX3CR1 APC/Fire 750 SA011F11 Biolegend 149040 1:400
CX3CR1 PE/Cy7 SA011F11 Biolegend 149016 1:400
Live/Dead DAPI (4',6-Diamidino-2-Phenylindole, Dilactate) (M) Biolegend 422801
ESAM (M) AF647 1G8 BD 567162 1:400
ESAM BUV737 1G8 BD 752446 1:200
FoxP3 AF488 150D Biolegend 320012 1:100
FoxP3 PE/Cy7 FJK-16s Invitrogen 25-5773-82 1:100
Gata3 BUV395 L50-823 BD 565448 1:100
Gata3 PE/Dazzle594 14-9966-82 eBioscience 14-9966-82 1:100
goat anti-mouse IgG, Fcγ fragment specific AF488 Polyclonal Jackson ImmunoResearch 115-545-008 1:400
goat anti-armenian hamster IgG (H+L) AF594 Polyclonal Jackson ImmunoResearch 127-585-160 1:400
goat anti-armenian hamster IgG (H+L) AF647 Polyclonal Jackson ImmunoResearch 127-605-160 1:400
GL7 AF647 GL7 Biolegend 144605 1:400
GL7 PE/Cy7 GL7 Biolegend 144619 1:400
Gr-1 Biotin RB6-8C5 Biolegend 108404 1:400
IFNγ AF647 XMG1.2 Biolegend 505814 1:100
IgA Biotin (M) RMA-1 Biolegend 407003 1:400
IgD BV711 11-26c2a Biolegend 405731 1:400
IgG BV605 Poly4053 Biolegend 405327 1:400
IgG1 AF647 RMG1-1 Biolegend 406617 1:400
IgG2a/c Biotin RMG2a-62 Biolegend 407104 1:400
IgG2a/c AF594 (M) RMG2a-62 Biolegend 407119 1:400
IgG2a/c APC/Fire 750 RMG2a-62 Biolegend 407123 1:400
IgG2b AF488 RMG2b-1 Biolegend 406717 1:400
IgG2b AF647 RMG2b-1 Biolegend 406715 1:400
IgG2b PE/Cy7 RMG2b-1 Biolegend 406713 1:400
IgG3 Biotin RMG3-1 Biolegend 406803 1:400
IgM AF488 RMM-1 Biolegend 406522 1:400
IgM BV510 RMM-1 Biolegend 406531 1:400
IL-17 BV421 TC11-18H10.1 Biolegend 506926 1:100
Ly6C AF700 HK1.4 Biolegend 128024 1:600
Ly6D FITC 49-H4 Biolegend 138606 1:400
Ly6G BV605 1A8 Biolegend 127639 1:400
MHC-II BV510 (M) M5/114.15.2 Biolegend 107636 1:800
MHC-II BV563 M5/114.15.2 BD 748848 1:400
MHC-II AF594 (M) M5/114.15.2 Biolegend 107650 1:400
MHC-II PerCP/Cy5.5 M5/114.15.2 Biolegend 107625 1:800
NK1.1 Biotin PK136 Biolegend 108704 1:400
NK1.1 BV605 PK136 Biolegend 108740 1:200
RORγt BV786 Q31 BD 564723 1:100
SAV BV510 Biolegend 405233 1:500
SAV YG570 (M only) Biolegend 405282 1:500
SAV BV605 Biolegend 405229 1:500
SAV BV650 Biolegend 405232 1:500
SAV BV711 Biolegend 405241 1:500
SAV BUV737 BD 612775 1:500
SAV PE/Cy7 Biolegend 405206 1:500
Sca1 BV785 D7 Biolegend 108139
SiglecH APC 551 Biolegend 129612 1:1000
SiglecH BUV661 440c BD 749806 1:800
SYBR Green Thermo Fisher Scientific S7563 1:10000
Tbet BUV737 O4-46 BD 568166 1:100
Tbet PE/Cy7 eBio4B10 eBioscience 25-5825-82 1:100

TCRβ BUV496 H57-597 BD 749915 1:400
TCRβ BV605 H57-597 Biolegend 109241 1:400
TCRγδ APC/Fire 750 GL3 Biolegend 118136 1:400
Ter119 Biotin TER-119 Biolegend 116204 1:400
TNF FITC MP6-XT22 Biolegend 506304 1:100
XCR1 BV421 ZET Biolegend 148216 1:200
XCR1 PE ZET Biolegend 148204 1:800
XCR1 BV650 ZET Biolegend 148220 1:200
Live/Dead Zombie Aqua Biolegend 423102 1:400

ELISA antibodies (concentration as indicated in methods):
C57BL/6 Mouse Immunoglobulin Panel Southern Biotech 5300-01B
goat anti-mouse IgM, human ads, UNLB Southern Biotech 1020-01
goat anti-mouse IgM, human ads-AP Southern Biotech 1020-04
goat anti-mouse IgA, UNLB Southern Biotech 1040-01
goat anti-mouse IgA, AP Southern Biotech 1040-04
goat anti-mouse IgG Fc-Total IgG, human ads, UNLB Southern Biotech 1030-01
goat anti-mouse IgG, human ads AP Southern Biotech 1030-04
goat anti-mouse IgG1, human ads, UNLB Southern Biotech 1070-01
goat anti-mouse IgG1, human ads-AP Southern Biotech 1070-04
goat anti-mouse IgG2b, human ads, UNLB Southern Biotech 1090-01
goat anti-mouse IgG2b, human ads-AP Southern Biotech 1090-04
goat anti-mouse IgG2c, human ads, UNLB Southern Biotech 1079-01
goat anti-mouse IgG2c, human ads-AP Southern Biotech 1079-04
goat anti-mouse IgG3, human ads, UNLB Southern Biotech 1100-01
goat anti-mouse IgG3, human ads-AP Southern Biotech 1100-04

Validation

All antibodies were validated by the manufacturer

Animals and other research organisms

Policy information about studies involving animals; ARRIVE guidelines recommended for reporting animal research, and Sex and Gender in Research

Laboratory animals

The following strains were bred and maintained on a C57BL/6 background: C57BL/6J, CD45.1, Notch2fl/fl, Notch2fl/flCD11cCre, Klf4fl/flCD11cCre, Flt3l-/-, Ccr2-/-, Rag2-/- and the respective parental strains were originally purchased from the Jackson Laboratory. Klf4fl/fl mice were obtained from Mutant Mouse Resource and Research Center (MMRC, line 29877). Serum from Aire-/- was obtained from Dr. M. Lionakis. Serum from Ifng 3'UTR deleted Adenylate-Uridylate Rich element (ARE-del-/-) was obtained from H. Young. Mice that were sex- and age-matched were used in the following age groups unless otherwise indicated: (i, young) 8-12 weeks of age, (ii, middle) 25-30 weeks of age (iii, old) over 50 weeks of age.

Wild animals

No wild animals were used

Reporting on sex

Both male and female mice were used for all experiments except for co-housing experiments. Since males should not be housed together after weaning, only females were used in this context. For cage-switch and fecal gavage experiments, mice from both sexes were used, and no sex-based biological differences in the outcome were observed.

Field-collected samples

For laboratory work with field-collected samples, describe all relevant parameters such as housing, maintenance, temperature, photoperiod and end-of-experiment protocol OR state that the study did not involve samples collected from the field.

Ethics oversight

Mice were maintained in the specific pathogen free (SPF) AAALAC-accredited NIDCR animal facility according to institutional guidelines (National Institutes of Health, license number 22-1111, 21-1063, LCIM-6).

Note that full information on the approval of the study protocol must also be provided in the manuscript.

Plants

Seed stocks

Report on the source of all seed stocks or other plant material used. If applicable, state the seed stock centre and catalogue number. If plant specimens were collected from the field, describe the collection location, date and sampling procedures.

Novel plant genotypes

Describe the methods by which all novel plant genotypes were produced. This includes those generated by transgenic approaches, gene editing, chemical/radiation-based mutagenesis and hybridization. For transgenic lines, describe the transformation method, the number of independent lines analyzed and the generation upon which experiments were performed. For gene-edited lines, describe the editor used, the endogenous sequence targeted for editing, the targeting guide RNA sequence (if applicable) and how the editor was applied.

Authentication

Describe any authentication procedures for each seed stock used or novel genotype generated. Describe any experiments used to assess the effect of a mutation and, where applicable, how potential secondary effects (e.g. second site T-DNA insertions, mosiacism, off-target gene editing) were examined.

Flow Cytometry

Plots

Confirm that:

☒ The axis labels state the marker and fluorochrome used (e.g. CD4-FITC).

☐ The axis scales are clearly visible. Include numbers along axes only for bottom left plot of group (a 'group' is an analysis of identical markers).

☒ All plots are contour plots with outliers or pseudocolor plots.

☒ A numerical value for number of cells or percentage (with statistics) is provided.

Methodology

Sample preparation	Myeloid cell suspensions from spleen, cervical (cLN), skin-draining inguinal (iLN), and mLN, lung and kidney were prepared by mechanical dissociation followed by digestion with DNaseI (0.1mg/ml) and Collagenase D (0.2mg/ml) in PBS/HEPES buffer (15mM HEPES, pH 7.3) for 30min at 37C using a magnetic stirrer. LN cells were washed and resuspended in MACS buffer (PBS, 0.5% BSA, 2 mM EDTA, pH 7.3). Bone marrow (BM) was isolated from the femurs and tibias by crushing in MACS buffer. Red blood cells (RBC) in spleen and BM suspensions were lysed with ammonium-chloride-potassium (ACK) buffer for 90s at 22C, after which cells were resuspended in MACS buffer. Lung and kidney suspensions were obtained following a 40% Percoll centrifugation and resuspension in MACS buffer. For B and T cell staining, lymphoid organ suspensions were prepared by mechanical dissociation followed by RBC lysis and resuspension in MACS buffer. PP were collected from small intestine (SI) and processed as LN. SI and colon (Co) were cut into ~1-inch segments and opened longitudinally after removal of fecal content and washed in PBS/HEPES buffer. Intraepithelial cells (IELs) were isolated by incubation in IEL buffer (PBS, 10% FCS, 15mM HEPES, 5mM EDTA, pH 7.3) for 20min at 22C under stirring. Supernatant containing IEL was washed and lymphocytes collected following a 40%-70% Percoll gradient centrifugation and resuspended in MACS buffer. SILP and CoLP suspensions were prepared by mechanical dissociation and digestion (SILP/CoLP: HBSS w/o $NaHCO_3$, 0.1mg/ml Dnasel, 0.2mg/ml Collagenase D, pH 7.3; CoLP addition: 1% fatty-acid-free BSA) for 50min at 37C using a magnetic stirrer, followed by 40% Percoll centrifugation and resuspension in MACS buffer.
Instrument	Cells were acquired using a BD LSRFortessa, BD FACSymphony A3 or Cytek Aurora 5L. Cells were sorted using a BD FACSAria II with a custom built-in violet laser or BD FACSymphony S6 Sorter.
Software	FlowJo v10 (Tree Star) and SpectroFlo (Cytek Biosciences) were used for data analysis. The LEGENDScreen data was analyzed using R (The R Foundation), RStudio (Posit PBC), the "infinityFlow" and mass-cytometry based packages as described in the manuscript.
Cell population abundance	Cells were sorted into MACS buffer to confirm a purity of more than 95% by post-sort analysis.
Gating strategy	Cells were pregated using FSC-A/SSC-A to identify lymphocytes based on size. Doublets were excluded by sequential gating on the diagonal singlet population within FSC-A/FSC-H, followed by SSC-A/SSC-H. Dead cells were excluded by gating on either 7-AAD-negative cells or Zombie Aqua-negative cells. Specific cell populations were gated as indicated in the manuscript.

☒ Tick this box to confirm that a figure exemplifying the gating strategy is provided in the Supplementary Information.
