# Supplementary material for: Dysbiosis-induced expansion of AXL-positive inflammatory type 3 dendritic cells triggers preclinical autoimmunity
Source: Nat Immunol. 2026 Jul 21;27(9):1856–73. doi: 10.1038/s41590-026-02599-z (PMC13506347; doi:10.1038/s41590-026-02599-z)
Supplement: Supplementary file 3 [file 41590_2026_2599_MOESM3_ESM.pdf]

nature portfolio

Peer Review File

Dysbiosis-induced expansion of AXL-positive inflammatory type 3 dendritic cells triggers pre-clinical autoimmunity

Corresponding Author: Dr Roxane Tussiwand

This file contains all editorial decision letters in order by version, followed by all author rebuttals in order by version.

Decision Letter:

11th Aug 2025

Dear Roxane,

Thank you for providing a point-by-point response to the referees' comments on your manuscript entitled, "AXL-positive type 3 dendritic cells promote dysbiosis and autoantibody production". As noted previously, while they find your work of considerable potential interest, they have raised quite substantial concerns that must be addressed. In light of these comments, we cannot accept the current manuscript for publication, but would be very interested in considering a revised version that addresses these serious concerns along the lines proposed in your point-by-point rebuttal.

We invite you to submit a substantially revised manuscript, however please bear in mind that we will be reluctant to approach the referees again in the absence of major revisions.

Specifically, the revision should include new experiments to address:

(1) rederivation of the Notch2-cKO mouse line to address the major concern posed by all 3 referees on whether the loss of Notch2 and resulting dysbiosis is causal or a consequence of the increased skewing/development of the inflammatory DC3 population.

(2) examine whether downregulation of Dnase1l3 is regulated by Notch2 or a result of dysbiosis

(3) determine if antibiotic treatment that reduced bacterial mass will also affect the DC3 compartment

(4) examine whether the Axl+ cDC3 subset is present in other tissues, such as lung, skin and kidney, at steady-state or upon dysbiosis

(5) examine the the ontogeny of the inf-DC3 cDC subset by using MS4a3-cre linage tracing experiments

(6) examine whether Notch2 plays a cell-intrinsic role in inF-cDC3 differentiation by performing BM transfers

Please include the additional textual clarifications as indicated in your response letter.

When you revise your manuscript, please take into account all reviewer and editor comments, please highlight all changes in the manuscript text file in Microsoft Word format.

We are committed to providing a fair and constructive peer-review process. Do not hesitate to contact us if there are specific requests from the reviewers that you believe are technically impossible or unlikely to yield a meaningful outcome.

When revising your manuscript:

* Include a “Response to referees” document detailing, point-by-point, how you addressed each referee comment. If no

action was taken to address a point, you must provide a compelling argument. This response will be sent back to the referees along with the revised manuscript.

* If you have not done so already please begin to revise your manuscript so that it conforms to our Article format instructions at http://www.nature.com/ni/authors/index.html. Refer also to any guidelines provided in this letter.

* Include a revised version of any required reporting checklist. It will be available to referees (and, potentially, statisticians) to aid in their evaluation if the manuscript goes back for peer review. A revised checklist is essential for re-review of the paper.

The Reporting Summary can be found here:
https://www.nature.com/documents/nr-reporting-summary.pdf

When submitting the revised version of your manuscript, please pay close attention to our href="https://www.nature.com/nature-portfolio/editorial-policies/image-integrity">Digital Image Integrity Guidelines. and to the following points below:

-- that unprocessed scans are clearly labelled and match the gels and western blots presented in figures.
-- that control panels for gels and western blots are appropriately described as loading on sample processing controls
-- all images in the paper are checked for duplication of panels and for splicing of gel lanes.

Extended Data figures and tables are online-only (appearing in the online PDF and full-text HTML version of the paper), peer-reviewed display items that provide essential background to the Article but are not included in the printed version of the paper due to space constraints or being of interest only to a few specialists. A maximum of ten Extended Data display items (figures and tables) is typically permitted. When re-submitting your manuscript, please ensure that any supplementary figures and tables that are more critical to the manuscript's conclusions are converted to Extended data to increase these data's visibility.

Finally, please ensure that you retain unprocessed data and metadata files after publication, ideally archiving data in perpetuity, as these may be requested during the peer review and production process or after publication if any issues arise.

You may use the link below to submit your revised manuscript and related files:

Link Redacted

Note: This URL links to your confidential home page and associated information about manuscripts you may have submitted, or that you are reviewing for us. If you wish to forward this email to co-authors, please delete the link to your homepage.

If you wish to submit a suitably revised manuscript we would hope to receive it within 6 months. If you cannot send it within this time, please let us know. We will be happy to consider your revision so long as nothing similar has been accepted for publication at Nature Immunology or published elsewhere.

Nature Immunology is committed to improving transparency in authorship. As part of our efforts in this direction, we are now requesting that all authors identified as 'corresponding author' on published papers create and link their Open Researcher and Contributor Identifier (ORCID) with their account on the Manuscript Tracking System (MTS), prior to acceptance. ORCID helps the scientific community achieve unambiguous attribution of all scholarly contributions. You can create and link your ORCID from the home page of the MTS by clicking on 'Modify my Springer Nature account'. For more information please visit please visit www.springernature.com/orcid.

Please do not hesitate to contact me if you have any questions or would like to discuss the required revisions further.

Thank you for the opportunity to review your work.

Kind regards,

Laurie

Laurie A. Dempsey, Ph.D.
Senior Editor
Nature Immunology
l.dempsey@us.nature.com
ORCID: 0000-0002-3304-796X

Version 0:

Reviewer comments:

Reviewer #1 (Remarks to the Author):

Notch2 deficiency impairs the differentiation of ESAM+ cDC2a. In the current manuscript, the authors demonstrate that conditional deletion of Notch2 in the DC lineage leads to broad changes across the DC compartment, with a significant increase in cDC3s, defined as ESAM-CD16/32+Cx3cr1+Axl+. Among the most significantly downregulated genes in Notch2-deficient cDCs was Dnase1l3, while cDC3 exhibited upregulation of inflammatory genes.
This subset accumulated in different tissues, including mLNs, spleen, SI and colon. Notch2cKO mice also displayed increased IgA and IgG titers in the serum and immune complex deposition in the kidney, along with intestinal dysbiosis and alterations in gut microbial composition. These mice were also more susceptible to DSS-induced colitis. Furthermore, co-housing or fecal transfer (from cKO) to WT mice recapitulated the dysbiotic phenotype and elevated Ab titers, both of which were reversed upon antibiotic treatment.

This is an interesting, well-written and well-designed manuscript that provides a significant finding for linking Notch2 with cDC3, gut dysbiosis, antibody (Ab) titers and preclinical features of autoimmunity.

I mostly have minor comments and requests for clarification:

The causal relation remains somewhat unclear. The authors show that the transferred dysbiosis (from Notch2cKO) induces increased Ab titers and expansion of cDC3 in WT mice, suggesting that dysbiosis alone can drive the accumulation of cDC3, likely independent of Notch2. This raises the question of whether cDC3 are drivers or responders in this context. Specifically, the role of cDC3 in promoting the increased Ab titers (as implied in the title) remains unclear.
Additionally, while the transcriptional differences between WT and Notch2-deficient cDC3 are described, it is not clear whether these changes are due to intrinsic loss of Notch2 or secondary to the altered immune and microbial environment (e.g. loss of cDC2a, changes in Th17 responses, decreased Tregs, or preclinical autoimmunity). A more in-depth discussion of these possibilities would be helpful. The title could also be toned down.

The downregulation of Dnase1l3 is interesting but remains somewhat unclear. Is Dnase1L3 also downregulated in WT cDC3 following fecal transfer? Is its downregulation Notch2-dependent, or is it a consequence of dysbiosis?
Are other cDCs in WT mice upon fecal transfer also changed in numbers?

Could the authors elaborate on how the cDC3 subset in their model compares to AXL+Siglec6+ cDCs and cDC3 in humans?

The increased Ab titers observed in co-housed or cKO mice was reversible upon antibiotics treatment. Did they also see a corresponding decrease in cDC3?

Could cDC3 directly be involved in Ab production by influencing B cells or plasma cells? Can they discuss this?

Is the AXL+ cDC3 subset increased in lymphoid (such as sLN) or non-lymphoid tissues, beyond the gut? For instance, in the lung, skin, or kidney? Or is the observed accumulation primarily restricted to gut-associated tissues?

Could the authors further elaborate on the lineage and ontogeny of the cDC3s in the absence of Notch2? Do these cells have a heterogenous origin? Several genes expressed by these cells (such as Ccr2, Csf1r, CD64, etc.) are typically associated with the monocyte/macrophage lineage. Their dependence on Crr2 but not Flt3, along with their substantial monocyte contribution (Fig. S2A), would further support this.

Minor comments:

Fig.1, it would be helpful if the authors annotated the clusters directly in Fig. 1A.
Furthermore, it would be better to omit CD45 and CD45.2 from the markers used for the UMAP to better detect genotype-specific clusters.
Also, for Fig. 1C, it is unclear which clusters from Fig. 1A were included in the analysis. Can the authors clarify which clusters in Fig. 1A correspond to these populations?

Fig. 1C, macrophages were detected in the WT but not in the cKO. Can they comment on this?

The cell number of total splenic cDC3 does not differ between WT and the cKO mice (Fig. S1I). However, using immunofluorescent imaging, cDC3 are not detected in the WT spleen (Fig. S1M). Are the cDC3 in a different location in comparison to the cKO cDC3? Alternatively, are the cDC3s detected in Fig. S1M specifically the AXL+ subset described in Fig. 2?
In Fig. 1I, it would be helpful to show single stainings for the markers in the insets, and to include an inset for the fl/fl control.

Color code is missing in Fig 1 and Fig. S1A.

Fig. 3G-J, it would be helpful to add total cell numbers in the tissue or per gram tissue, and not just frequencies among CD45+ cells.

Reviewer #2 (Remarks to the Author):

The manuscript by Cvijetic et al. provides evidence that that Notch2 deficiency in CD11c+ cells leads to broad changes in the abundance and transcriptional phenotypes of dendritic cells, beyond the previously reported absence of cDC2a. Initially the authors use flow cytometry of splenic dendritic cells and later scRNA-seq analysis of dendritic cells from spleen, mLN and sLN of Notch2cKO to identify changes in dendritic cell subsets. The study then focuses on a population of inflammatory cDC2 (inf-cDC2), here suggested to be a variant of monocyte-derived DC3 (inf-DC3). The authors show that Notch2cKO mice have increased numbers of inf-DC3 and this is accompanied by microbial dysbiosis, increased susceptibility to colitis and enhanced autoantibody production. The authors propose that inf-DC3 play a direct role in regulation of microbiota and autoimmunity.

The role of inf-cDC2/DC3 has not previously been established and the study addresses an important question. The characterization of Notch2cKO mice is extensive and the authors have established a number of important phenotypes. However, the manuscript would benefit from methodological and conceptual refinements to definitively establish the role of “inf-DC3” in the phenotypes described.

Major comments
The most notable absence is any direct evidence linking inf-DC3 to the ensuing immune dysregulation and disease phenotypes in Notch2cKO mice. Although the authors suggest a direct role for inf-DC3 in inflammation that arises in Notch2cKO mice, inflammation drives inf-DC3 differentiation therefore the increase in inf-DC3 could be a consequence rather than a cause of inflammation in these mice. The authors demonstrate many differences in CD11c+ populations (both abundance and differentially expressed genes) by scRNA-seq analysis which could lead to immune dysregulation. Besides the analysis of DC subsets in Fig. 2, CD11c-cre is a very non-specifc cre driver that targets not only dendritic cells, monocytes and macrophages, but also specific subsets of B cells and T cells. To determine the direct role of inf_DC3 in intestinal inflammation, more precise cre-drivers are needed to target DC3 such as Ms4a3. A cell intrinsic role for Notch2 in restraining inf-DC3 differentiation should also be assessed using mixed bone marrow chimeras.
Related to this, loss of Notch signaling leads to impaired intestinal Th17 differentiation attributed to loss of IL23+ (either CD103+CD11b+ or CD103– CD11b+ cDC2s (Lewis et al. Immunity 2011, Ohara et al. JEM 2025). The loss of Th17 cells would likely lead to alterations in the intestinal microbiota that are independent of inf_DC3. The authors should discuss these previous studies in more detail, and perform additional experiments to exclude a role for conventional DC2 subsets such as cDC2a, cDC2b or IL23+ cDC2s in dysbiosis and autoimmune phenotypes, for example using Clec9a-cre Rbpj flox mice which are available.

CD11c-cre will also target CD11c expressing B cells which include populations of antibody producing B cells that have been implicated in autoimmune disease. Although the authors include an analysis of MZB cells, additional experiments should be performed to clarify whether the dysregulated antibody production observed in Notch2cKO mice is due to a cell intrinsic role of Notch2 in CD11c+ B cells.

The authors perform a comprehensive scRNA-seq analysis of DCs within the spleen, mLN and sLN but the analysis of DC subsets in the intestinal lamina propria, the site of inflammation, is limited to flow cytometry characterization and relies on CD16/32 being a conserved DC3 marker across tissues. The authors state that they focused on inf_DC3 because of the pronounced increase in inf_DC3 in spleen of Notch2cKO mice, yet in mLN, there is only a modest increase in inf_DC3 in Notch2cKO mice (Fig. S2c). I was unclear whether the authors were proposing that the increase in inf_DC3 was responsible for ensuing dysbiosis. If so, this requires some additional onceptual and mechanistic explanation. The authors should perform scRNA-seq of DCs from WT vs Notch2cKO mice from the intestinal epithelium and lamina propria to confirm that inf_DC3 are increased at these sites and to determine if there are other relevant changes in DC populations that may be responsible for dysbiosis.

A second major point relates to the definitions of cDC2b/DC3 subsets. The study relies on the use of CD16/CD32 to distinguish cDC2b vs DC3. Although one study has described the existence of distinct cDC2b vs DC3 populations (Rodrigues et al. Immunity 2024), the original description of monocytic DC3 by Liu et al suggested that cDC2b and DC3 were synonymous. Integration of published datasets in which cDC2b or DC3 were described are needed to understand how these transcriptionally defined subsets relate to each other. In Fig. 2A and F, cluster 2 is annotated as a mixed DC3 and cDC2b cluster. If this is the case, these cells could be resolved by sub-clustering, similar to the analysis performed by Rodrigues et al., Immunity 2024. The authors should perform subclustering to see if there are 2 transcriptionally distinct cell types present in cluster 2. However, it is notable that there appears to be uniform Fcgr2b in this cluster but no Fcgr3 expression. This is at odds with the authors proposed definition of DC3 as CD16/32+ cells and the flow cytometry characterization of cluster 2/cDC2b as CD16/32-CX3CR1+ cells and cluster 2/steady-state/WT DC3 as CD16/32+CX3CR1+AXL- cells. Since the study relies on CD16/CD32 as the distinguishing marker for DC3, prospective isolation and scRNA-seq analysis of Esam–CX3CR1+CD16/32+ DC3 vs Esam–CX3CR1+CD16/32– cDCb cells and integration with previous scRNA-seq datasets describing DC3 or cDC2b cells is warranted.

In addition, the use of CD16/CD32 as a discriminatory cDC2b/DC3 marker has not been validated in inflammatory settings. It is possible that cDC2b upregulate CD16/CD32 in these contexts. Given the closely overlapping transcriptomes of cDC2b and DC3 which were only resolved into separate subsets based on fine sub-clustering and lineage tracing. To define inf DC3 cells as DC3 rather than inf cDC2b cells, lineage tracing experiments are required to confirm that the cells characterized as inf DC3 are fate-mapped by Ms4a3-cre (Liu et al. Immunity 2023) rather than Clec9a-cre (Minutti et al.

Nature Immunology 2024).

The definition of cDC2b/DC3 is particularly confusing in Fig. 1E where the authors identify only cDC2a and DC3 clusters in cells isolated from WT mice but no cDC2b whereas cDC2b are present in Notch2cKO. This is at odds with previous studies from Reis e Sousa, Rudensky and Colonna labs which showed that cDC2b were present in the spleen at steady state. On what basis are the authors ascribing clusters 2 and 7 as DC3 rather than cDC2b? The scRNA_seq analysis in Figure 2 gives a much more informative overview of cDC2 heterogeneity which renders Fig. 1 somewhat redundant and would perhaps be better as supplemental data to support Fig. 2.

Minor comments:

Abstract: the authors state that NOTCH2 deficiency impairs the development of ESAM+ type 2a DC2s resulting in defective intestinal TH17 cells but it would be more accurate to state that NOTCH2 deficiency impairs the development of ESAM+ type 2a DC2s and intestinal CD103+CD11b+ cells (which have not been shown to represent cDC2a). Thus far the defect in TH17 cell differentiation has been linked to the IL23+ cDC2 population.

Within the introduction and Fig. 2A, the authors suggest that Rorc-expressing DCs are cDC2s. My understanding is that the definition of Rorc-expressing DCs as cDC2 stemmed from a previous study by Rudensky and this has been superseded by several studies which have shown that these cells are developmentally distinct from conventional dendritic cells, and only a subset of Rorc-expressing APCs express CD11b. In addition, their ontogeny has not been established. A recent study by Colonna's group suggested that they were CLP-derived and more closely related to ILCs. Without supporting evidence, the statements related to Rorc DC ontogeny and their inclusion in the cDC2 family should be omitted as this will only add to the considerable confusion in the field relating to this cell type.

Fig. 1I and S1M – despite the use of CD16/32 as a marker for DC3, the authors did not identify CD16/32 DC3 in WT mice. This requires further explanation.

Reviewer #3 (Remarks to the Author):

Grozdan et al. revisit CD11cCre: Notch2fl/fl mice, exploring the possibility that the Notch2 deficiency not only results in the reported loss of ESAM+ cDC2a, but also additional alterations of the DC compartment. Detailed comparative analysis of mutants and controls, including comprehensive phenotype and transcriptome analyses revealed the accumulation of a cell population resembling a recently described DC3 subset. Using scRNASeq of sorted populations, the authors show that in particular in peripheral tissues, i.e. the intestine, these DC3-like cells cells show prominent expression of Axl and a pro-inflammatory gene signature, including TNF and IL6 expression. Furthermore, DC of Cd11cCre: Notch2fl/fl mice displayed reduced expression of DNaseI|3, which has previously been linked to immuno-pathology. Focusing on the intestine, the authors show that the DC3-like cells accumulate in GALT-like aggregates with T and B cells. Moreover, CD11cCre: Notch2fl/fl mice exhibit increased serum IgA and IgG2b/c serum titers and immune complex depositions in the kidneys. CD11cCre: Notch2fl/fl mice also show dysbiosis, and indeed, the authors show that co-housing of wt animals with mutants induces the accumulation of the DC3-like cells and the serum antibody changes. Moreover, they identify three dominant bacterial species (Parasutterella, Mucispirillum, Chlamydia) driving the alterations.

All data presented by the authors are of high standard, and the experiments are performed with rigor. The study contains a plethora of valuable information; however, concerns remain with respect to the general message and interpretation.

Major points

The authors suggest a causal link between DC dysfunction and systemic autoimmunity. Specifically, their title states that DC3-like cells are inducers of the phenotype, but this statement is problematic. To establish the causal role of DC3-like cells, one would arguably have to perform adoptive transfers. Likewise, to evaluate whether DC3 emergence is intrinsic to Notch2-deficient pre-DCs, adoptive transfer of CD11cCre: Notch2fl/fl pre-DCs into WT recipients followed by assessment of DC3 development from the donor cells.
The question arises whether the accumulation of DC3-like cells and dysbiosis is also observed in Notch2-deficient animals in other facilities or whether the observation is somewhat anecdotal.
As it stands, the Notch2-deficient animals allowed the authors to reveal some potential interesting information on DC3-like cells, the Notch-control of DNAse expression, and on the effect of pathobionts on humoral immunity. However, none of these promising threads are pursued to a conclusive end.

It also remains unclear which of the DC alterations are due to cell-intrinsic effects of the Notch deficiency and which are secondary effects due to environmental changes. It would be good to highlight which of the DC populations express Notch2 and In which DC populations are direct Notch targets such as Hes1 affected (see Fig. 2E) ? Mixed BM chimeras could further help dissect intrinsic effects.

The fact that co-housing, i.e. the mere microbiota transfer, results in an increase of Axl+ DC3 cells, alongside elevated autoantibody levels, challenges the notion of a causal link between DC3 and dysbiosis/autoimmunity. Would the DC3 expansion occur in germ-free or broad-spectrum antibiotic-treated CD11cCre; Notch2fl/fl mice? Is also the DNaseI|3

deficiency observed under dysbiosis in wt mice or is it strictly linked to the Notch2 deficiency?

Peripheral tissue resident DC3 populations remain less well characterized, particularly with regard to lineage tracing with a decisive discriminating factor being that these cells are not derived from monocytes. While splenic and BM DC3 populations were identified using established gating strategies, it is unclear whether these criteria are valid for intestinal DC, whose marker expression often differs by tissue. Notably, the use of CD64, a common intestinal macrophage marker (Tamoutounour et al.) to define DC3 is concerning.
The observation that the CD64+ DC3 accumulation is abrogated in CCR2-deficient mice, and that the DC3 increase compensates for the DC2 loss in Flt3l KO mice, would support monocyte-derivation. Likewise, Klf4 is also required for monocyte development (Kurotaki et al.)
Thorough clarification of the identity of colonic DC3, including Axl-expressing populations, would require single-cell RNA-seq analysis of colonic myeloid cells, including both DC and macrophages, as was done in spleen and LNs. Furthermore, administration of neutralizing anti-CSF1R antibodies may help distinguish intestinal DC3 from macrophages based on differential depletion (see Nonaka et al. 2025).

Related to the above, the discussion states: “DC3 were recently identified as a distinct DC subset that is transcriptionally similar to, but developmentally distinct from monocyte-derived DC.” However, to our knowledge, no study has definitively demonstrated a developmental distinction between DC3 and monocyte-derived DCs. If such literature exists, it should be cited.

Minor points

It is well established that gut DC require retinoic acid for proper differentiation and function, with ALDH enzymes playing a key role in RA synthesis, thereby promoting Treg induction and IgA production (Erkelens et al., 2017). The authors should analyze expression of Aldh genes in intestinal DCs of CD11cCre; Notch2fl/fl mice. Additionally, treatment of these mice with exogenous RA may clarify whether RA supplementation can ameliorate autoimmune-like pathology.

IL-23 is a known driver of Th17-mediated autoimmune diseases such as IBD, RA, and SLE, and IL-23p19 knockout mice show reduced disease severity in colitis models (Yen et al., 2006). that CD11cCre; Notch2fl/fl mice were reported to lack IL-23-producing DC (Ohara et al., 2024). The autoimmune phenotype associated with dysbiosis in Notch2-deficient mice suggests hence a potentially mechanism distinct from classical IL-23–Th17-mediated IBD, a point deserving further discussion.

- Fig. 1I, 5J, S5I
Microscopy images indicating DC3 localization are difficult to interpret. Including DAPI or other structural markers to visualize tissue context and simplifying the color combination would significantly improve clarity.

- Fig. 1G,H
How were DC3 defined for sorting for bulk RNAseq ?

- Fig. 4K
The claim that Akkermansia reads are reduced by co-housing lacks sufficient statistical support. Please include appropriate statistical analysis.

- Fig. 5H, I, N–P
To better assess whether DC3 changes are statistically significant compared to other cell types, frequencies should be expressed as a proportion of total CD45+ cells rather than cDC2 alone, and compared with cDC2a and cDC2b using appropriate statistical tests.

- Fig. 6L, M
Please include body weight curves to complement the DSS-induced colitis data.

- Fig. 6, S6.
Please specify the starting age and duration of co-housing in the methods or figure legend.

Decision Letter:

5th May 2026

Dear Roxane,

Thank you for providing a point-by-point response to the second round of external review for your manuscript entitled "Dysbiosis-induced expansion of AXL-positive inflammatory type 3 dendritic cells triggers pre-clinical autoimmunity", which was seen by the original three referees. We are very interested in the possibility of publishing your study in Nature Immunology, but would like to first revise the manuscript as indicated in your rebuttal letter before we make a final decision

on publication. I think the additional textual clarifications and framing of the response as indicated will be sufficient without further experiments to establish a definitive developmental origin for the cell population.

We therefore invite you to revise your manuscript taking into account all reviewer and editor comments. Please highlight all changes in the manuscript text file in Microsoft Word format.

Additionally, please convert your Supplementary figures into Extended Data Figures to increase the visibility of these data. Please note that articles can have up to 8 main figures and 10 Extended data figures. Note, however, that some of the current Supplementary figures are too large in having more that 12-13 panels per figure (several have more than 20 panels, eg Supplementary Fig. 5w) or extending over 2 pages. Please be judicious in what data need to be depicted in either the main or extended data figures. A static supplementary PDF with Supplementary data is also possible, but these will not pop up in HTML readers. Also, note that all panel callouts should use lowercase letters. Also, please avoid using stippled shading in the bar graphs shown in Fig. 7m,o and Supplementary Fig. 7o,p,q (just use another solid color).

We are committed to providing a fair and constructive peer-review process. Do not hesitate to contact us if there are specific requests from the reviewers that you believe are technically impossible or unlikely to yield a meaningful outcome.

When revising your manuscript:

* Include a “Response to referees” document detailing, point-by-point, how you addressed each referee comment. If no action was taken to address a point, you must provide a compelling argument. This response will be sent back to the referees along with the revised manuscript.

* If you have not done so already please begin to revise your manuscript so that it conforms to our Article format instructions at http://www.nature.com/ni/authors/index.html. Refer also to any guidelines provided in this letter.

* Please include a revised version of any required reporting checklist. It will be available to referees to aid in their evaluation of the manuscript goes back for peer review. They are available here:

Reporting summary:
https://www.nature.com/documents/nr-reporting-summary.pdf

When submitting the revised version of your manuscript, please pay close attention to our href="https://www.nature.com/nature-portfolio/editorial-policies/image-integrity">Digital Image Integrity Guidelines. and to the following points below:

-- that unprocessed scans are clearly labelled and match the gels and western blots presented in figures.
-- that control panels for gels and western blots are appropriately described as loading on sample processing controls
-- all images in the paper are checked for duplication of panels and for splicing of gel lanes.

Please note, Extended Data figures and tables are online-only (appearing in the online PDF and full-text HTML version of the paper), peer-reviewed display items that provide essential background to the Article but are not included in the printed version of the paper due to space constraints or being of interest only to a few specialists. A maximum of ten Extended Data display items (figures and tables) is typically permitted. When re-submitting your manuscript, please ensure that any supplementary figures and tables that are more critical to the manuscript’s conclusions are converted to Extended data to increase these data’s visibility.

Finally, please ensure that you retain unprocessed data and metadata files after publication, ideally archiving data in perpetuity, as these may be requested during the peer review and production process or after publication if any issues arise.

Please use the link below to submit your revised manuscript and related files:

Link Redacted

Note: This URL links to your confidential home page and associated information about manuscripts you may have submitted, or that you are reviewing for us. If you wish to forward this email to co-authors, please delete the link to your homepage.

We hope to receive your revised manuscript by mid-June 2026. If you cannot send it within this time, please let us know. We will be happy to consider your revision so long as nothing similar has been accepted for publication at Nature Immunology or published elsewhere.

Please do not hesitate to contact me if you have any questions or would like to discuss these revisions further.

Nature Immunology is committed to improving transparency in authorship. As part of our efforts in this direction, we are now requesting that all authors identified as ‘corresponding author’ on published papers create and link their Open Researcher and Contributor Identifier (ORCID) with their account on the Manuscript Tracking System (MTS), prior to acceptance. ORCID

helps the scientific community achieve unambiguous attribution of all scholarly contributions. You can create and link your ORCID from the home page of the MTS by clicking on ‘Modify my Springer Nature account’. For more information please visit please visit www.springernature.com/orcid.

We look forward to seeing the revised manuscript and thank you for the opportunity to review your work.

Kind regards,

Laurie

Laurie A. Dempsey, Ph.D.
Senior Editor
Nature Immunology
l.dempsey@us.nature.com
ORCID: 0000-0002-3304-796X

Version 1:

Reviewers' Comments:

Reviewer #1 (Remarks to the Author):

The authors have done an excellent job addressing all of my comments and points. I have no further remarks and fully support the publication.

Reviewer #2 (Remarks to the Author):

The authors have undertaken a substantial series of additional experiments to address the prior comments, including fecal transfer studies, rederivation of cKO mice, and detailed phenotypic analysis of intestinal DC populations by flow cytometry and scRNA-seq. Collectively, these revisions provide important new insight into the sequence of events leading to dysbiosis and autoantibody production, as well as the associated changes in DC compartments across disease progression. I have only two remaining comments regarding the revised manuscript.

The authors identify a subset of CD64–CD16/32+ “inf DC3” cells that is expanded in rederived cKO mice and propose that this population is causally involved in the initiating dysbiosis marked by outgrowth of three pathobionts. However, in the absence of genetic tools to selectively target this subset, or candidate effector molecules such as Dnase1l3, it remains unclear why this population, rather than the cDC2a subset that is also reduced in cKO mice, should be considered the primary driver of dysbiosis. An alternative interpretation is that loss of cDC2a initiates dysbiosis.

I still find the definition of DC3 somewhat confusing. The DC3 subset was defined by the Ginhoux lab and the same lab subsequently reported that these cells were derived from monocyte progenitors, implying that DC3 represents a monocyte-derived DC population. Ms4a3-cre is a very efficient lineage tracer. Thus, the incomplete Ms4a3 fate mapping observed in CD16/CD32+ CD11b+ DCs likely reflects the fact that CD16/CD32 does not discriminate monocyte derived DC3 from CDP derived cDC2b, as reported by Nguyen et al. (PMID: 41272166). It would therefore seem more accurate to describe this population as CD16/32+ cDC2b/DC3. Do the authors observe differences in the proportion of Ms4a3 fate mapped cells within CD64+ vs CD64– CD16/32 DC3 populations?

Reviewer #3 (Remarks to the Author):

The manuscript remains complex but the clarification of causality is a clear improvement.
The authors have responded to most of our points, but some issues remain and are listed below.

Related to point #28
We understand the technical challenges of pre-DC transfer experiments, but it is problematic to dismiss lineage testing solely on the ground of limited cell numbers. Flt3L-Fc could be used to expand DC progenitors and enable adoptive transfer experiments. Although the mixed BM chimera data support a competitive advantage and/or enhanced intrinsic survival or proliferation of Notch2-deficient cells, they do not directly demonstrate that DC3 are derived from CD135+ DC progenitors. Defining a cell type requires not only phenotypic characterization but also clarification of its developmental origin. (https://www.sciencedirect.com/science/article/pii/S1074761320301631)

Related to point #35
CD26 has been proposed as a marker to distinguish DCs from macrophages under steady-state conditions, but to our knowledge, it has not been established as a reliable marker of intestinal DCs under inflammatory conditions. Therefore, when defining a DC3 population in the intestine, where such populations have been only minimally characterized, caution is required when assigning these cells as macrophages or DCs, particularly with respect to their developmental origin.

Moreover, the Ms4a3 fate-mapping data do not conclusively exclude a monocytic origin. It is known that, in Ms4a3 fate-mapping mice, the labeling efficiency of tissue macrophages is lower than that of circulating blood monocytes. Therefore, simply comparing the labeling frequency of the putative inf-DC3 population with that of blood monocytes does not provide sufficient evidence that these cells are not monocyte-derived. The statement “These findings reveal DC3 as a DC lineage phenotypically related to but developmentally different from monocytes and DC2s.” is too strong and should be modified.

Related to point #36
It is known that macrophages also express Axl (https://www.science.org/doi/10.1126/science.aai8132). This has to kept in mind when proposing Axl as marker for identifying DC3.

Related to point #36
The results obtained in CCR2-deficient and Flt3l-deficient mice would lead many readers to interpret the DC3 population discussed by the authors as being derived from CCR2-dependent monocytes, rather than as an Flt3L-dependent DC-lineage population.
Assessing dependence on growth and survival signals is useful for defining cellular identity. Therefore, the authors should examine whether this population depends on CSF1R signaling.
https://www.sciencedirect.com/science/article/pii/S1074761320304532

Decision Letter:

Our ref: NI-A40692B

14th May 2026

Dear Roxane,

Thank you for addressing the remaining referee concerns for your manuscript entitled "Dysbiosis-induced expansion of AXL-positive inflammatory type 3 dendritic cells triggers pre-clinical autoimmunity" (NI-A40692B). I have discussed these changes with the others on the editorial team, and I am please to announce that we are happy in principle to publish it in Nature Immunology, pending minor revisions to comply with our editorial and formatting guidelines.

We will now perform detailed checks on your paper and will send you a checklist detailing our editorial and formatting requirements in about a week. Please do not upload the final materials and make any revisions until you receive this additional information from us.

If you had not uploaded a Word file for the current version of the manuscript, we will need one before beginning the editing process; please email that to immunology@us.nature.com at your earliest convenience.

Thank you again for your interest in Nature Immunology Please do not hesitate to contact me if you have any questions.

Best regards,

Laurie

Laurie A. Dempsey, Ph.D.
Senior Editor
Nature Immunology
l.dempsey@us.nature.com
ORCID: 0000-0002-3304-796X

Open Access This Peer Review File is licensed under a Creative Commons Attribution 4.0 International License, which permits use, sharing, adaptation, distribution and reproduction in any medium or format, as long as you give appropriate credit to the original author(s) and the source, provide a link to the Creative Commons license, and indicate if changes were made.
In cases where reviewers are anonymous, credit should be given to 'Anonymous Referee' and the source.
The images or other third party material in this Peer Review File are included in the article's Creative Commons license, unless indicated otherwise in a credit line to the material. If material is not included in the article's Creative Commons license and your intended use is not permitted by statutory regulation or exceeds the permitted use, you will need to obtain permission directly from the copyright holder.
To view a copy of this license, visit https://creativecommons.org/licenses/by/4.0/

Point by point revision

Overall

We have extensively revised our study and have now incorporated several new experiments including rederived *Notch2*^{cKO}, time-course experiments, bone marrow chimeras, antibiotic-treated mice, as well as a new single-cell RNA sequencing dataset generated from mLN and colonic DC isolated from WT, dysbiotic WT, rederived *Notch2*^{cKO}, and dysbiotic *Notch2*^{cKO} mice. These new experiments are presented in two new figures: new Fig. 7, S7 and new Fig. 8, S8. In addition, several new panels have been included to address specific reviewers' concerns. These changes are labeled as new figures (new Fig. X) to facilitate navigation. Major text revisions and changes are highlighted in blue. Following reviewers' suggestions and in light of the new data, the title has been revised to: "**Dysbiosis-induced expansion of AXL-positive inflammatory type 3 dendritic cells triggers pre-clinical autoimmunity**"

Finally, several authors that contributed with key reagents have been added to the manuscript author list. For clarity, in this point-by-point reply we have included selective panels but referred to the corresponding figures and new figures from the final draft.

Reviewer#1

Notch2 deficiency impairs the differentiation of ESAM+ cDC2a. In the current manuscript, the authors demonstrate that conditional deletion of Notch2 in the DC lineage leads to broad changes across the DC compartment, with a significant increase in cDC3s, defined as ESAM-CD16/32+Cx3cr1+Axl+. Among the most significantly downregulated genes in Notch2-deficient cDCs was Dnase1l3, while cDC3 exhibited upregulation of inflammatory genes. This subset accumulated in different tissues, including mLNs, spleen, SI and colon. Notch2cKO mice also displayed increased IgA and IgG titers in the serum and immune complex deposition in the kidney, along with intestinal dysbiosis and alterations in gut microbial composition. These mice were also more susceptible to DSS-induced colitis. Furthermore, co-housing or fecal transfer (from cKO) to WT mice recapitulated the dysbiotic phenotype and elevated Ab titers, both of which were reversed upon antibiotic treatment.

This is an interesting, well-written and well-designed manuscript that provides a significant finding for linking Notch2 with cDC3, gut dysbiosis, antibody (Ab) titers and preclinical features of autoimmunity.

We are very grateful for the positive feedback on our study.

I mostly have minor comments and requests for clarification:

Point 1

The causal relation remains somewhat unclear. The authors show that the transferred dysbiosis (from Notch2cKO) induces increased Ab titers and expansion of cDC3 in WT mice, suggesting that dysbiosis alone can drive the accumulation of cDC3, likely independent of Notch2. This raises the question of whether cDC3 are drivers or responders in this context.

This was indeed an open question of the study and a major point raised by all three reviewers. The *Notch2*^{cKO} strain has at one point acquired the dysbiotic environment and whether the strain intrinsically had an expansion of the inf-DC3 compartment which may have favored the colonization or whether the colonization induced the sustained expansion of the DC3 to counteract the bacterial burden remained to be resolved. Further, how DC3 were responsible for increased antibody titers was also unresolved. We addressed these questions combining several experimental approaches. The most conclusive result was obtained by rederiving the line under SPF conditions (new Fig. 7G-J and S7I-M). The rederivation would ensure a pathobiont-free intestinal colony and allow us to determine if the DC3 compartment is pre-expanded in response to the genetic deficiency or a consequence of the dysbiosis. As shown in new Fig. 7I-J and S7L and below in **Fig. R1**, rederived *Notch2*^{cKO} (Red-*Notch2*^{cKO}) and WT mice have comparably low frequencies and absolute numbers of AXL^{+}CD64^{+} inf-DC3 in the colon, mLN, as well as spleen, suggesting that this subset is responding to the colonization by the pathobiont.

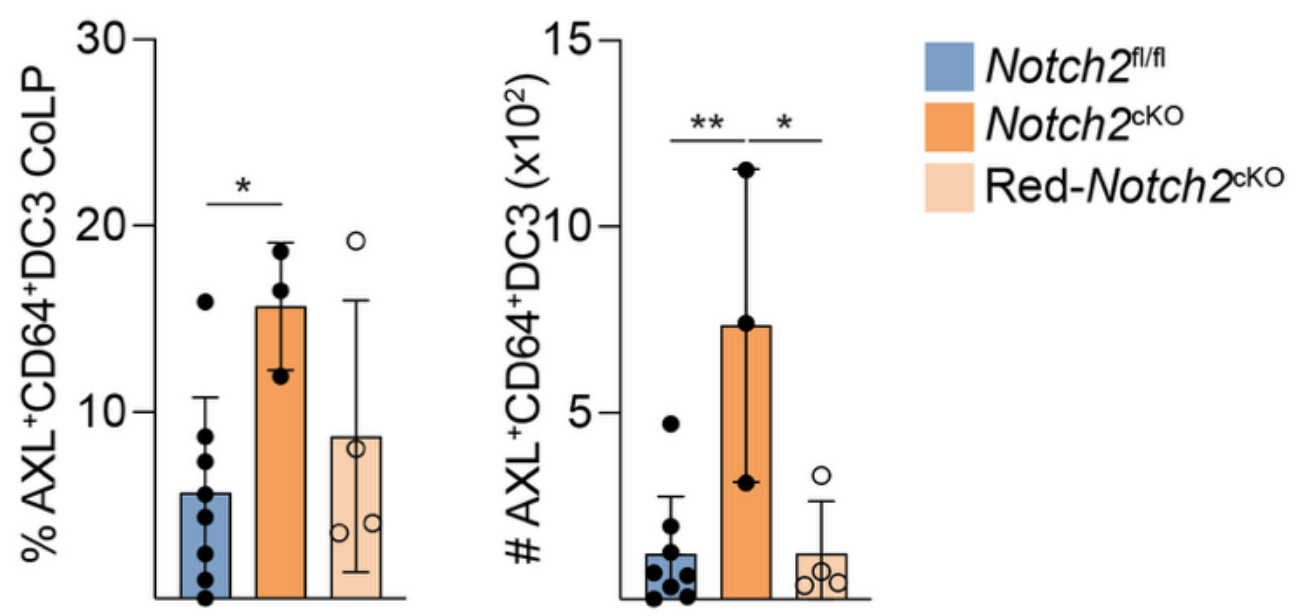

Fig. R1 (Fig. 7I). Steady-state analysis of colonic AXL$^+$CD64$^+$DC3 in *Notch2*$^{fl/fl}$ (blue), *Notch2*^{cKO} (orange), and rederived *Notch2*^{cKO} (light orange) mice. Shown are frequencies (left) and total cell numbers (right) of AXL$^+$CD64$^+$DC3, pre-gated as CD11c$^+$MHCII$^+$CD26$^+$XCR1$^-$CD16/32$^+$.

However, Red-*Notch2*^{cKO} mice had increased frequencies and total AXL$^+$CD64$^-$inf-DC3 cells (new Fig. S7I-J and S7M and **Fig. R2**), suggesting that this subset is expanding in response to the genetic defect.

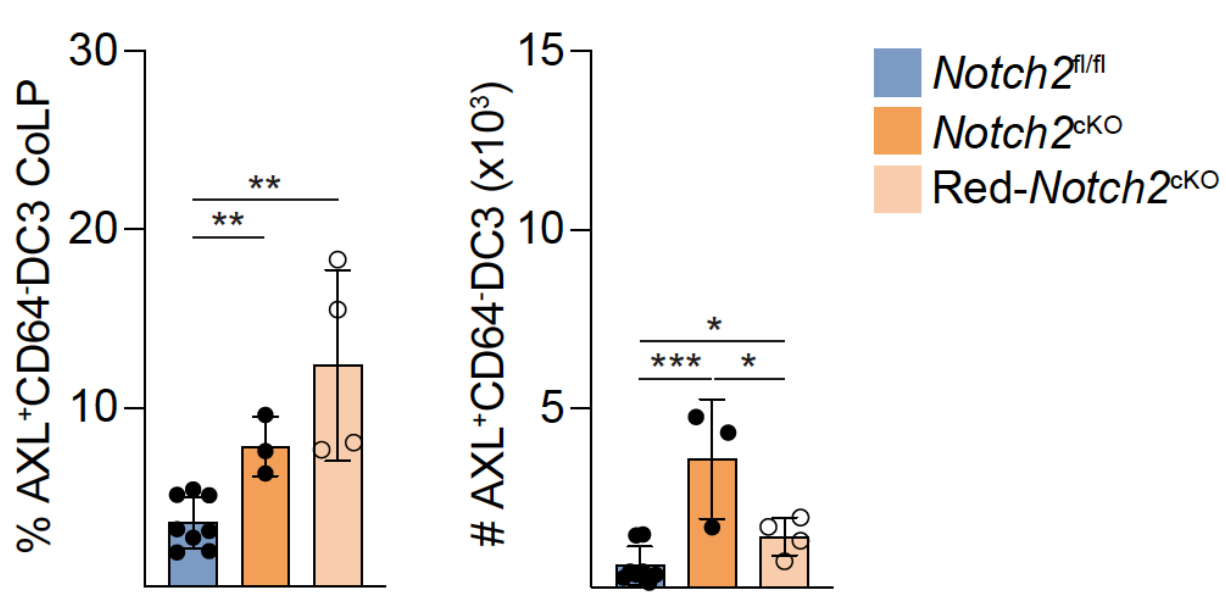

Fig. R2 (Fig. S7I). Steady-state analysis of colonic AXL$^+$CD64$^-$DC3 in *Notch2*$^{fl/fl}$ (blue), *Notch2*^{cKO} (orange), and rederived *Notch2*^{cKO} (light orange) mice. Shown are frequencies (left) and total cell numbers (right) of AXL$^+$CD64$^-$DC3, pre-gated as CD11c$^+$MHCII$^+$CD26$^+$XCR1$^-$CD16/32$^+$.

This level of resolution for AXL$^+$inf-DC3, into two subsets, namely AXL$^+$CD64$^-$ and AXL$^+$CD64$^+$, became apparent during the revision process and was validated by scRNA-seq data. Both subsets of AXL$^+$inf-DC3 are present at negligible frequencies and absolute numbers in WT mice (**Fig. R1 and R2**). CD64$^-$ cells are expanded in the colon of *Notch2*^{cKO} mice, both rederived and dysbiotic, suggesting that this subset may be responsible for the increased susceptibility that characterizes *Notch2*^{cKO} mice to enteropathogenic infections and DSS-induced colitis.

In both strains, WT and *Notch2*^{cKO}, CD64^{+}inf-DC3 expand upon dysbiosis, suggesting that these cells are indeed responders to changes in the microbiome.

Antibiotic treatment of *Notch2*^{cKO} resulted in the contraction of AXL^{+}CD64^{+}inf-DC3 (new Fig. 7D-F), reflecting a reduction in bacterial biomass including the three pathobionts as determined by 16S, while AXL^{+}CD64^{-}inf-DC3 remained expanded (new Fig. S7E-F, S7H) further strengthening the genetically-driven expansion of this subset in line with the Red-*Notch2*^{cKO} data.

We further generated mixed BM chimeras (new Fig. 7K-O and S7N-R), which would enable us to discriminate between cell-intrinsic and cell-extrinsic properties of the two subsets. Also in these settings, AXL^{+}CD64^{+}inf-DC3 did not expand in lethally irradiated recipient mice receiving WT or cKO total BM cells (new Fig. 7L, 7N and S7N), reflecting the results obtained from Red-*Notch2*^{cKO} and antibiotic treated mice. Nevertheless, we cannot exclude a possible contraction of the pool by the antibiotic treatment, that is required by the experimental procedure, as we observed in Fig. 7D-F.

The genetically-driven expansion of AXL^{+}CD64^{-} inf-DC3 was evident when donor cells were of cKO origin (new Fig. 7M, O and S7O), in line with the rederivation and antibiotics experiments. This subset was also expanded in mixed BM chimeras, suggesting a cell-intrinsic effect. As expected also cDC2a were absent in BM chimeras.

Collectively, we confirmed via three independent approaches that AXL^{+}CD64^{+}inf-DC3 are a specific subset responding to dysbiosis.

To characterize the features of these subsets in WT and cKO mice, we generated scRNAseq-data comparing mLN and colonic DCs isolated from WT and rederived cKO at steady-state as well as WT and cKO mice in dybiosis. The complete analysis is now plotted in new Figures 8 and S8. Despite the low abundancy of immune cells within the colon, we managed to obtain sufficient cell numbers. We validated the expansion of AXL^{+}CD64^{+}inf-DC3 (cluster 9) in response to dysbiosis, and the expansion of AXL^{+}CD64^{-} inf-DC3 in response to *Notch2* deficiency (cluster 10). A detailed analysis of the sequencing data allowed us to provide an in-depth characterization of the inflammatory profile for both inf-DC3 subsets.

Since, both AXL^{+}inf-DC3 display a similar inflammatory transcriptional signature, it is possible that they may represent distinct tissue-driven activation states of the same population or reflect

developmentally related but separate lineages. Future studies, that would enable selective targeting, are required but are beyond the scope of this study.

Point 2

Specifically, the role of cDC3 in promoting the increased Ab titers (as implied in the title) remains unclear.

We have adapted the title, which is now "**Dysbiosis-induced expansion of AXL-positive inflammatory type 3 dendritic cells triggers pre-clinical autoimmunity**" to emphasize that the dysbiosis and the DC3 subset that is expanded in the context of dysbiosis is relevant for the induction of autoimmunity. The evidence comes from the new set of experiments in new Fig. 7 and 8 that include the analysis of Red-*Notch2*^{cKO}, that neither show signs of autoimmunity nor increased antibody titers (new Fig. 8A-B), and the data obtained through scRNA-seq of colon and mLNs from steady-state WT and cKO (Red-*Notch2*^{cKO}) mice as well as WT and cKO (*Notch2*^{cKO}) mice colonized by the pathobionts. All together, we could show that the dysbiotic environment was causal to the generation of these preclinical autoimmune symptoms, and also that it directly correlated with the expansion of AXL^{+}CD64^{+}inf-DC3 (cluster 9), whose transcriptional profile showed upregulation of inflammatory genes and genes related to antigen processing and presentation (new Fig. 8). In addition, the kinetic of pathobiont colonization (Fig. 4J, L), inf-DC3 expansion (new Fig. 7A-C, **Fig. R3**) and increase in antibody titers (Fig. 4G-H, S4L, R, new S7S), collectively support our hypothesis. Further, close proximity of IgA-producing B cells, T cells, and AXL^{+}inf-DC3 are strongly implying the formation of functional immune clusters/tertiary lymphoid structures promoting the observed antibody response.

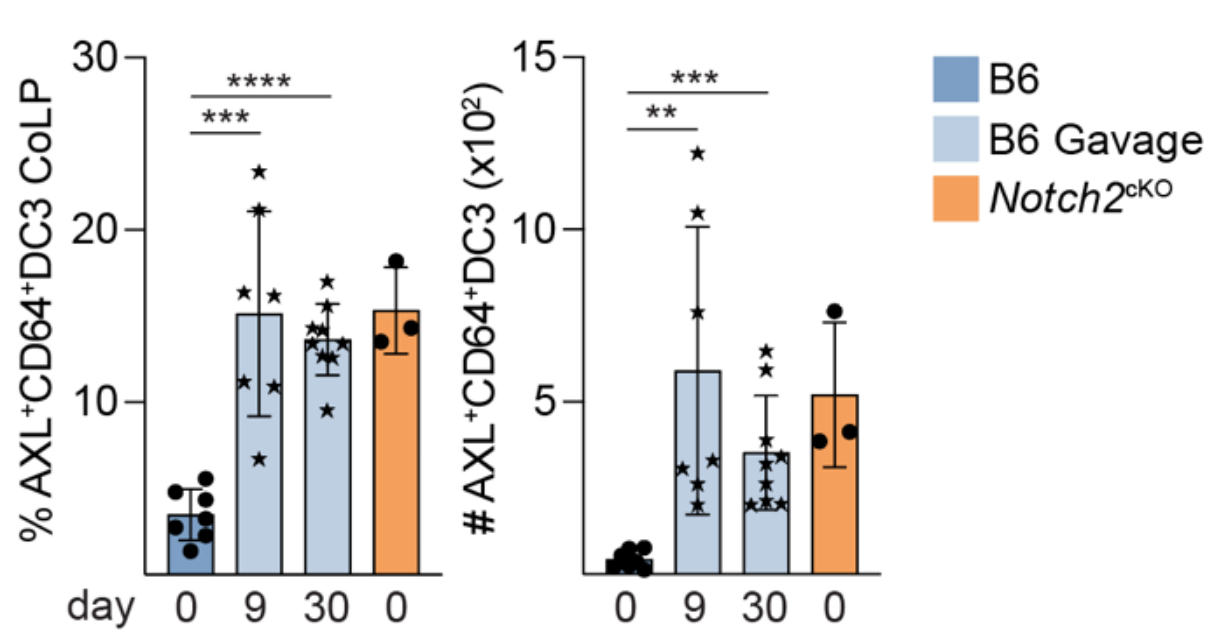

Fig. R3 (Fig. 7B). Analysis of colonic AXL$^+$CD64$^+$DC3 at the indicated time-points in control C57BL/6 (dark blue), C57BL/6 gavaged with *Notch2*^{cKO} fecal material (light blue), and *Notch2*^{cKO} (orange) mice. Shown are frequencies (left) and total cell numbers (right) of AXL$^+$CD64$^+$DC3, pre-gated as CD11c$^+$MHCII$^+$CD26$^+$XCR1$^-$CD16/32$^+$.

Unfortunately, currently available genetic tools do not enable specific deletion of either or both subsets. Nevertheless, to the best of our capabilities we could provide sufficient evidence in support of our hypothesis.

Point 3

Additionally, while the transcriptional differences between WT and Notch2-deficient cDC3 are described, it is not clear whether these changes are due to intrinsic loss of Notch2 or secondary to the altered immune and microbial environment (e.g. loss of cDC2a, changes in Th17 responses, decreased Tregs, or preclinical autoimmunity). A more in-depth discussion of these possibilities would be helpful.

As stated above (Point 1) we generated more data including a detailed analysis of the DC compartments in Red-*Notch2*^{cKO}.

Further, following the requests by the other two reviewers, we decided to generate scRNA-seq data on WT, Red-*Notch2*^{cKO}, WT-colonized, and *Notch2*^{cKO}-colonized mLN and colon DCs (see Point 2). This comparison allowed us to clearly dissect the transcriptional difference that is related to the genotype and define the changes in response to the altered microbiome as shown in new Fig. 8 and S8. We have further extended the discussion as requested, discussing changes driven by the genetic deficiency and by the dysbiosis.

Point 4

The title could also be toned down.

To be more in line with the new results, we have tweaked the title to: "**Dysbiosis-induced expansion of AXL-positive inflammatory type 3 dendritic cells triggers pre-clinical autoimmunity".**

The results obtained on Red-*Notch2*^{cKO} mice and in BM chimeras, the expansion of inf-DC3 upon fecal gavage and their contraction upon antibiotic treatment, which directly correlated

with antibody titers, collectively provide compelling evidence to support our statement and hypothesis (New figures 7, 8 and S7 and S8).

AXL$^+$CD64$^+$inf-DC3 are characterized by a strong inflammatory profile (new Fig. 8) that triggers a concerted tissue response with the formation of tertiary lymphoid structures and geminal center reactions (Fig. 5). Collectively, AXL$^+$inf-DC3 promote elevated antibody titers and the generation of autoantibodies, forming immune complexes that accumulate in the kidney. Despite the lack of proteinuria, such manifestation is compatible with pre-clinical autoimmune symptoms.

Point 5

The downregulation of Dnase1l3 is interesting but remains somewhat unclear. Is Dnase1L3 also downregulated in WT cDC3 following fecal transfer?

We have performed the requested experiment and observe induction of DNASE1L3 expression across all DC subsets in WT mice, with an increase from 20% to 50-70% for colonic DC3 (**Fig. R4)**. The expression data shown below in **Fig. R4,** was obtained following the generation of a new mouse line that replaced the endogenous locus with a DNASE1L3-mNeonGreen fusion protein followed by an in-frame cassette expressing the truncated extra-cellular domain of the human CD4. This mouse is currently being analyzed and will not be included in the current study.

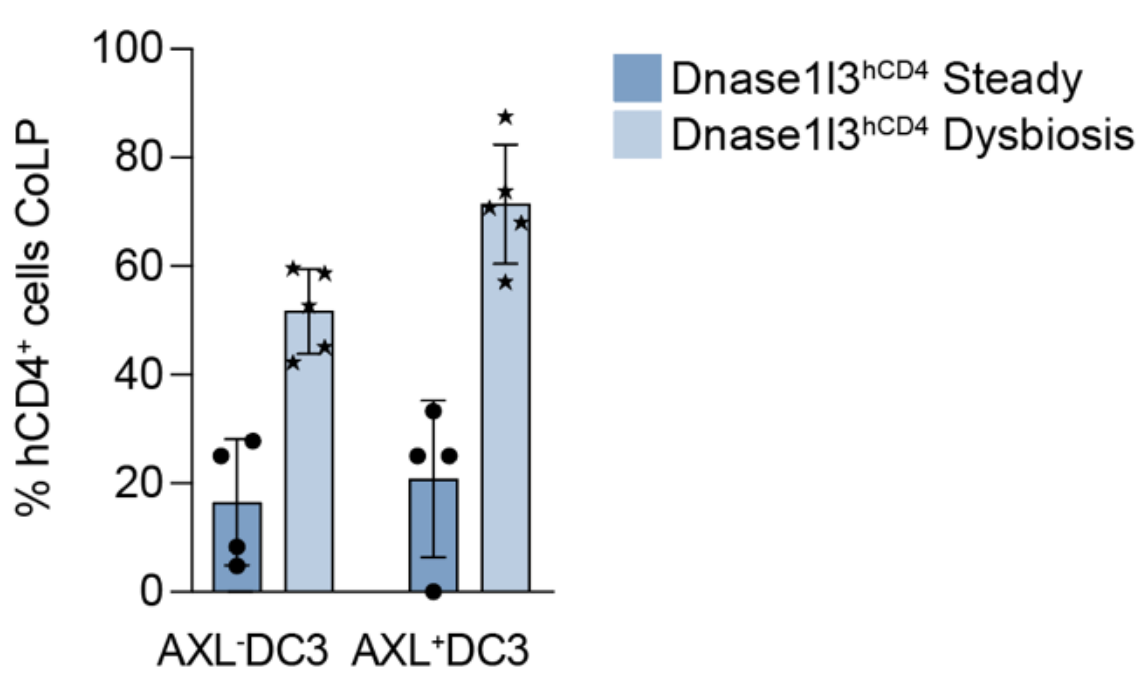

Fig. R4. Shown is the quantification of DNASE1L3 expression, traced by the induction of hCD4 in DC3 and AXL$^+$CD64$^+$DC3, pre-gated as CD11c$^+$MHCII$^+$CD26$^+$XCR1$^-$CD16/32$^+$, under steady-state conditions and following induction of dysbiosis by fecal transfer.

ScRNA-seq data showed also an increase in the percent of cells expressing *Dnase1l3* across most subsets within the colon (new Fig. S8Q). DC in mLN and spleen shown by scRNA-seq and flow

cytometry analysis have high expression levels across multiple subsets independently of the dysbiosis (Fig. 2L, new Fig. S8Q).

Point 6

Is its downregulation Notch2-dependent, or is it a consequence of dysbiosis?

In WT cells *Dnase1l3* is exclusively induced in the colon following dysbiosis, but is already high across all DC subsets within lymphoid tissues. *Notch2*^{cKO} mice lack the expression of this gene at steady-state and under dysbiosis, suggesting regulation by NOTCH2.

The obtained data collectively suggests that induction of DNASE1L3, is *Notch2*-dependent and that its upregulation occurs in response to dysbiosis within the local tissue. Since this enzyme is secreted, it is possible that it may be a mechanism to limit inflammation or reduce pathobiont colonization by degrading biofilms[1] or endogenous DNA released in response to tissue damage. The susceptibility and exacerbated phenotype observed in *Notch2*^{cKO} mice could reflect this impaired mechanism. Future studies are required to address this aspect.

Point 7

Are other cDCs in WT mice upon fecal transfer also changed in numbers?

In order to address this point in depth, we have generated scRNAseq-data on DCs isolated from mLN and colon, comparing WT, Red-*Notch2*^{cKO}, WT-colonized, and *Notch2*^{cKO}-colonized mice (new Figure 8). Further, flow cytometry analysis expanding the characterization of DC subsets across multiple tissue is now shown in new Figure S5I-L. Briefly, spleen shows no changes in DCs numbers and frequency in dysbiotic WT (new Fig. S5I); mLN show an exclusive expansion of AXL^{+}CD64^{+}DC3 (new Fig. S5J and **Fig. R5)**; within the colon we observe expansion of DC2 and DC3 (new Fig. S5K), but AXL^{+}CD64^{+}DC3 are the subset showing the highest fold change (new Fig. S5L).

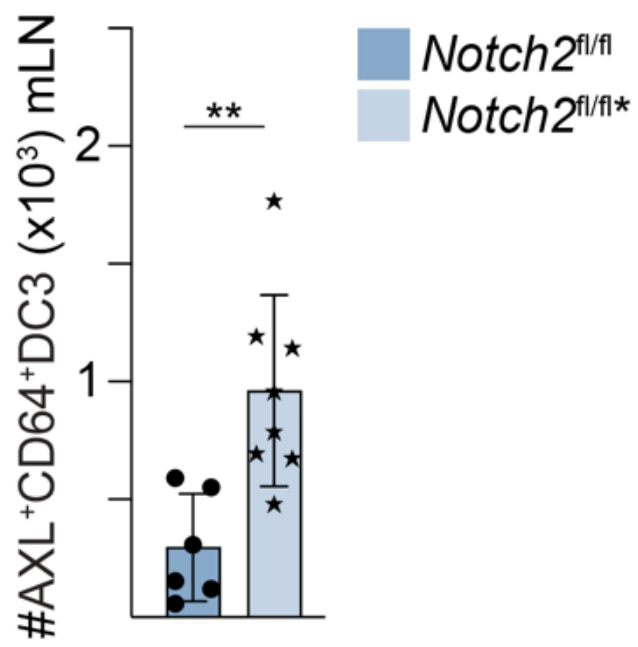

Fig. R5. Total cell numbers of AXL^{+}CD64^{+}DC3, pre-gated as CD11c^{+}MHCII^{+}CD26^{+}XCR1^{-}CD16/32^{+}, in mLN of *Notch2*$^{fl/fl}$ (dark blue) and dysbiotic *Notch2*$^{fl/fl}$* (light blue) mice.

Point 8

Could the authors elaborate on how the cDC3 subset in their model compares to AXL+Siglec6+ cDCs and cDC3 in humans?

The previously identified circulating AXL^{+}Siglec6^{+} human DC subset[2, 3] was shown to transcriptionally align close to pDCs, and correspond to murine pDC-like/tDC (cluster 6 in Fig. 2 and Fig. 8), that are different to the here described DC3 subset (clusters 2 and 5 in Fig. 2 and clusters 9 and 10 in Figure 8).

Point 9

The increased Ab titers observed in co-housed or cKO mice was reversible upon antibiotics treatment. Did they also see a corresponding decrease in cDC3?

Yes, we observe a direct correlation between dysbiosis and AXL^{+}inf-DC3 numbers. This subset expands upon fecal transfer and establishment of dysbiosis, sustaining inflammation (new Fig. 7A-C, Fig. R3), and possibly counteracting the inability of the immune system to clear these pathobionts (Fig. 4J, L). Antibiotic treatment resulted in the contraction of inf-DC3 in the colon, and in a reduction of antibody titers, supporting the hypothesis that this subset is promoting local

and systemic immune responses to the colonization by opportunistic and pathogenic species (new Fig. 7D-F, **Fig. R6**).

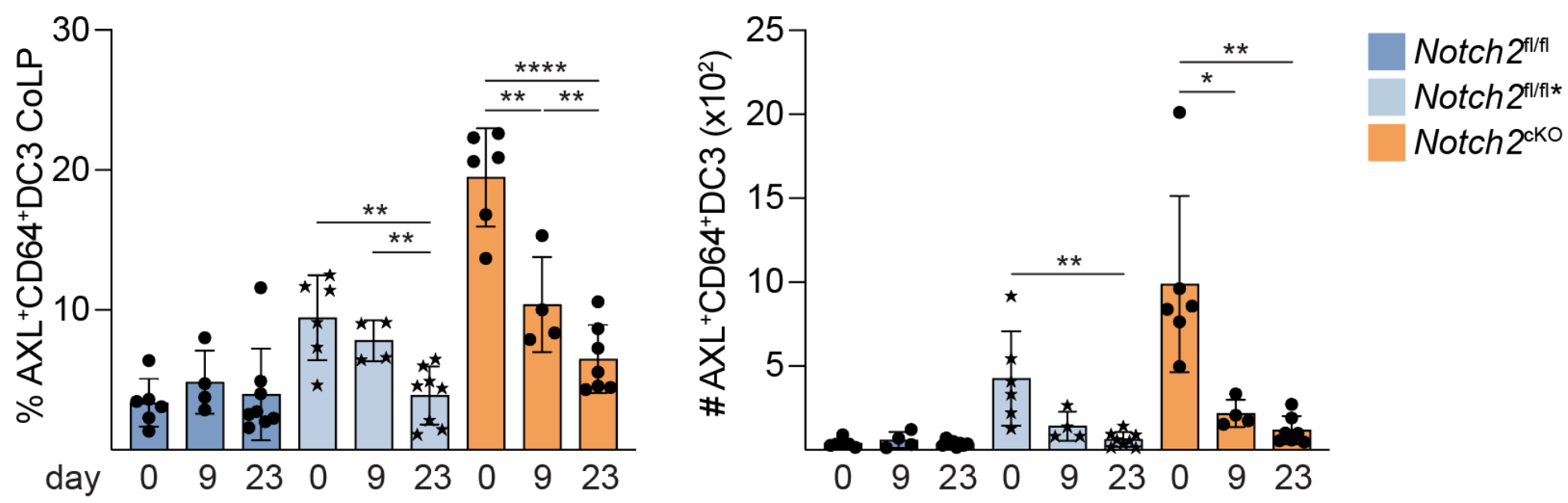

Fig. R6 (Fig. 7E). Antibiotics were administered to *Notch2*$^{fl/fl}$ (dark blue), dysbiotic *Notch2*$^{fl/fl}$* (light blue), and *Notch2*^{cKO} (orange) via drinking water for 9 days, followed by regular water for 2 weeks (day 23). Shown are the frequencies (left) and total cell numbers (right) of colonic AXL^{+}CD64^{+}DC3, pre-gated as CD11c^{+}MHCII^{+}CD26^{+}XCR1^{-}CD16/32^{+}.

Point 10

Could cDC3 directly be involved in Ab production by influencing B cells or plasma cells? Can they discuss this?

We tried to address this aspect to the best of our capacity as bacterial FISH and DC staining are incompatible and one does not preserve the antigens while the other does not allow for use of FISH probes. We have generated sequential slides and multidimensional immune-histochemistry to determine the proximity of IgA expressing B cells, T cells and DC clusters. As shown in original Figure 5 we find all relevant immune subsets co-localizing, suggesting that DC are presenting antigens to promote the induction of antibody responses.
Further, scRNA-seq data show that DC3 within the colon and mLNs show a significant upregulation of genes related to antigen processing and presentation, including the expression of members of the immune proteasome, i.e. *Psmb*.

Collectively, localization, transcriptional profile, cytokine production and the kinetic of DC3 expansion suggest that DC3 are necessary for Tfh induction and antibody production/class switch recombination, that results in high antibody titers. We have extended this point in the discussion as suggested.

Point 11

Is the AXL+ cDC3 subset increased in lymphoid (such as sLN) or non-lymphoid tissues, beyond the gut? For instance, in the lung, skin, or kidney? Or is the observed accumulation primarily restricted to gut-associated tissues?

We have specifically addressed this point by analyzing lung, skin, skin-draining LNs and kidney DCs. As shown in new Fig. S2K and **Fig. R7**, each tissue appears to have different frequencies of DC3 and inf-DC3, with the lowest number of cells identified in skin and the highest number of cells in colon, kidney and lung at steady-state, further supporting the hypothesis that monocellular epithelial layers highly exposed to the environment are enriched in this subset.

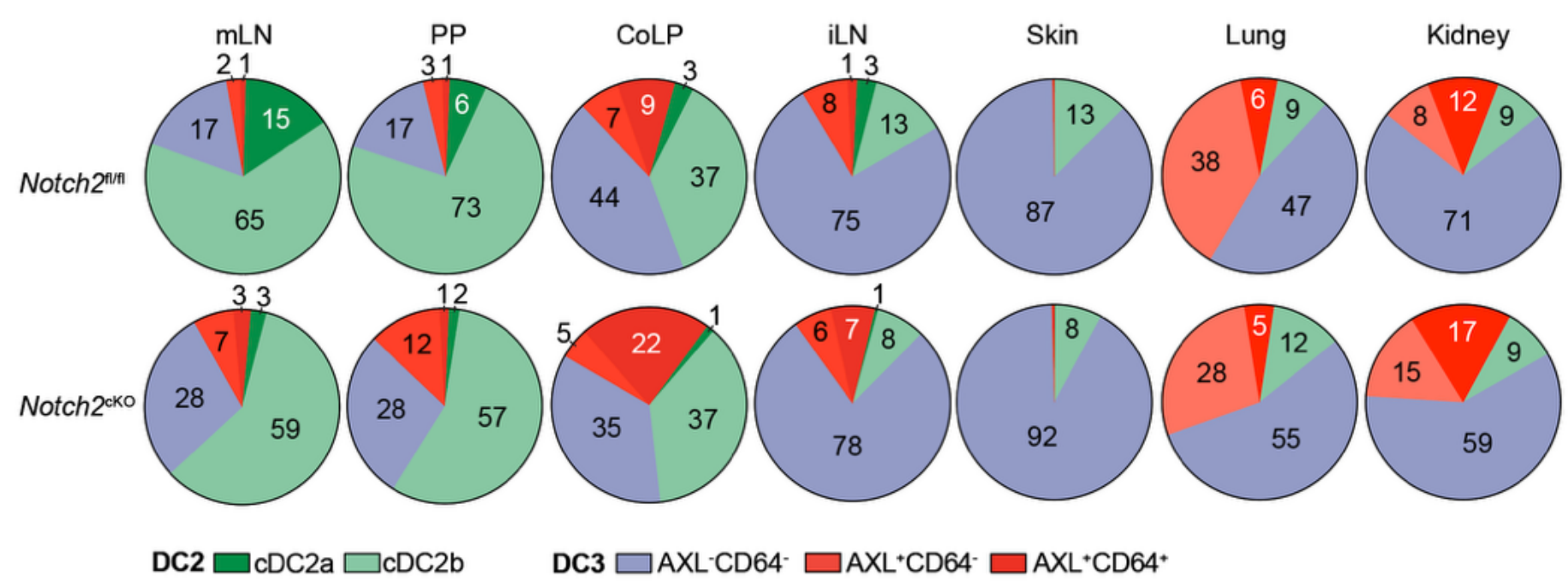

Fig. R7 (Fig. S2K). Shown are average frequencies of DC2 and DC3 subsets across the indicated tissues.

Point 12

Could the authors further elaborate on the lineage and ontogeny of the cDC3s in the absence of Notch2? Do these cells have a heterogenous origin? Several genes expressed by these cells (such as Ccr2, Csf1r, CD64, etc.) are typically associated with the monocyte/macrophage lineage. Their dependence on Crr2 but not Flt3, along with their substantial monocyte contribution (Fig. S2A), would further support this.

This is an important but complex question as we learn more and more about the intricacy and heterogeneity of DC lineages, its states upon inflammation, and tissue specificity that depends on

different cues, which appear to be co-regulated by multiple cytokines and chemokines, including GM-CSF, FLT3 and CCR2. While the community is still addressing this important aspect, we have tried our best by presenting the data obtained for different KO lines that were available in our colony. We have now included in the manuscript the analysis of DC3 and inf-DC3 obtained following fecal transfer in *Ms4a3*^{Cre}/*Rosa26*^{tdTomato} (New Figure S5W). *Ms4a3*^{Cre} traces monocyte and GMP-derived DCs. As shown, cDC1, cDC2a and cDC2b are poorly labelled and dysbiosis does not appear to change the frequency of labeling, suggesting that these are as previously stated CMP-derived. DC3 and inf-DC3 show a higher frequency of labeling around 30-50% at steady-state, which however remains unchanged upon dysbiosis. We can therefore imply that the expanding inf-DC3 are not monocyte- nor GMP-derived as the frequency of labelled cells does not reach the high frequency close to 100% for monocytes. We can however not unequivocally determine the origin of DC3/inf-DC3 based on their heterogenous labeling. They could be partially GMP- (30-50% tdT positive) and partially CMP (50%-70% tdT negative) derived. Alternatively, they may arise form a yet to be identified precursor, which would show this labeling frequency.

Minor comments:

Fig.1, it would be helpful if the authors annotated the clusters directly in Fig. 1A. Furthermore, it would be better to omit CD45 and CD45.2 from the markers used for the UMAP to better detect genotype-specific clusters. Also, for Fig. 1C, it is unclear which clusters from Fig. 1A were included in the analysis. Can the authors clarify which clusters in Fig. 1A correspond to these populations?

The plots in Fig 1A are relatively small for allowing all clusters to be labeled. We therefore introduced some key clusters and annotated the subsets on top of the markers in both Fig. 1A and S1A (i.e. Ly6G (Neutrophils), NK1.1 (NK cells) etc). The CD45 and CD45.2 markers were used purposefully to segregate WT and KO cells. Their exclusion as backbone markers from the analysis pipeline would not allow their reintroduction once the clustering is achieved and therefore preclude us from determining the genotype. We keep the backbone marker for the UMAP generation but

perform cluster analysis excluding both CD45 and CD45.2. As shown shared and genotype-specific clusters can still be displayed following this approach. We have clarified this aspect in the main text and method section.
Fig. S1D shows the gating strategy for the population analyzed in Fig. 1B-E. The figure legend was adjusted for clarity as requested.

Fig. 1C, macrophages were detected in the WT but not in the cKO. Can they comment on this?

Macrophages are not lost in cKO sample but just appear to have reduced CD11c expression which resulted in their exclusion based on a pre-gating strategy on CD11c-positive cells. We have added a comment in the main text to clarify this point.

The cell number of total splenic cDC3 does not differ between WT and the cKO mice (Fig. S1I). However, using immunofluorescent imaging, cDC3 are not detected in the WT spleen (Fig. S1M). Are the cDC3 in a different location in comparison to the cKO cDC3? Alternatively, are the cDC3s detected in Fig. S1M specifically the AXL+ subset described in Fig. 2?

This is correct and indeed it may lead to confusion; total cDC3s are not significantly different (Fig. S1I) between WT and KO in the spleen, their expansion in cKO or dysbiotic mice is confined to the gut-associated tissues. However, in the spleen the number of AXL^{+}inf-DC3 increases drastically from 2 to 3-fold (new Fig. S5I, and **Fig. R8**).

Further, as correctly pointed out, in WT mice DC3 are mostly localized around follicles in proximity of the marginal zone, while in cKO mice the cells are localized in the center of follicles (Fig. 1I, S1M-N).

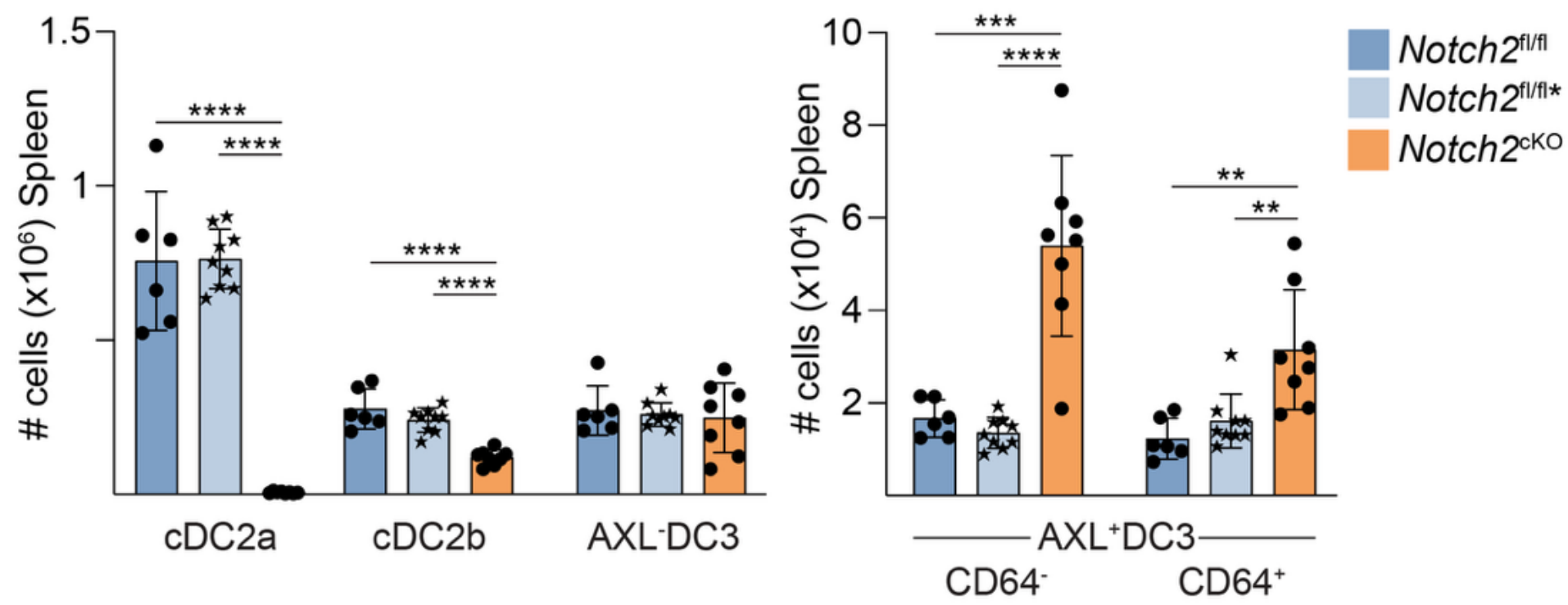

Fig. R8. Shown is the expansion of AXL+inf-DC3 in cKO mice.

In Fig. 1I, it would be helpful to show single stainings for the markers in the insets, and to include an inset for the fl/fl control.

The single-color stainings are now shown in Fig. S1M-N. For floxed mice there is no inset as cells which correspond to the identified subset are not present within follicles but only at its margin. We have adapted the main text to make sure this information is clear.

Color code is missing in Fig 1 and Fig. S1A.

We have now included the legend with the color code.

Fig. 3G-J, it would be helpful to add total cell numbers in the tissue or per gram tissue, and not just frequencies among CD45+ cells.

Figure 3 and S3 have been adapted to include absolute numbers.

Reviewer #2

(Remarks to the Author)

The manuscript by Cvijetic et al. provides evidence that that Notch2 deficiency in CD11c+ cells

leads to broad changes in the abundance and transcriptional phenotypes of dendritic cells, beyond the previously reported absence of cDC2a. Initially the authors use flow cytometry of splenic dendritic cells and later scRNA-seq analysis of dendritic cells from spleen, mLN and sLN of Notch2cKO to identify changes in dendritic cell subsets. The study then focuses on a population of inflammatory cDC2 (inf-cDC2), here suggested to be a variant of monocyte-derived DC3 (inf-DC3). The authors show that Notch2cKO mice have increased numbers of inf-DC3 and this is accompanied by microbial dysbiosis, increased susceptibility to colitis and enhanced autoantibody production. The authors propose that inf-DC3 play a direct role in regulation of microbiota and autoimmunity.

The role of inf-cDC2/DC3 has not previously been established and the study addresses an important question. The characterization of Notch2cKO mice is extensive and the authors have established a number of important phenotypes. However, the manuscript would benefit from methodological and conceptual refinements to definitively establish the role of "inf-DC3" in the phenotypes described.

We are grateful for the positive feedback. We have extensively refined the study including scRNA-seq, BM chimeras, rederivation, detailed time-course experiments, antibiotic treatment, and in-depth DC3 characterization.

Major comments

Point 13

The most notable absence is any direct evidence linking inf-DC3 to the ensuing immune dysregulation and disease phenotypes in Notch2cKO mice. Although the authors suggest a direct role for inf-DC3 in inflammation that arises in Notch2cKO mice, inflammation drives inf-DC3 differentiation therefore the increase in inf-DC3 could be a consequence rather than a cause of inflammation in these mice. The authors demonstrate many differences in CD11c+ populations (both abundance and differentially expressed genes) by scRNA-seq analysis which could lead to immune dysregulation.

We agree with the reviewer that many changes in DC subset are observed in this line and could reflect the phenotype observed, however inf-DC3 show similar expansion and transcriptional changes in WT compared to cKO mice upon dysbiosis. In this subset, we detected the highest expression of inflammatory genes. Inf-DC3 appear forming tertiary lymphoid structures co-localizing with T and IgA-producing B cells (Fig. 5).

Nevertheless, as this was a major point for all three reviewers and indeed a weakness of our study, we decided to investigate further. To determine if the inf-DC3 expansion is intrinsic to the phenotype or the consequence of the dysbiosis in *Notch2*^{cKO} mice we have rederived the line. In rederived *Notch2*^{cKO} (Red-*Notch2*^{cKO}) inf-DC3 are at similar frequencies compared to WT mice. Colonization by pathobionts leads to specific expansion of AXL^{+}CD64^{+}DC3. Contraction of the subset occurs upon antibiotic usage and reflects the reduction in microbiome mass and pathobionts reads. This observation is further supported by the generation of BM chimeras. A detailed explanation is provided in Point 1, Point 2, and Point 9. We have also generated scRNA-seq on WT, Red-*Notch2*^{cKO}, as well as dysbiotic WT and *Notch2*^{cKO} mice. The extended data are now displayed in new Fig. 7, S7, 8 and S8.

Point 14

Besides the analysis of DC subsets in Fig. 2, CD11c-cre is a very non-specifc cre driver that targets not only dendritic cells, monocytes and macrophages, but also specific subsets of B cells and T cells. To determine the direct role of inf-DC3 in intestinal inflammation, more precise cre-drivers are needed to target DC3 such as Ms4a3.

We had considered this option ourselves, however none of the available Cre lines would be more precise than the one we had used as the *Ms4a3* would target only 30-50% of DC3 and all monocytes, leaving alternative interpretations open. Other Cre-lines that we had considered such are the LyzM-cre would also affect both subsets. However, we took advantage of the *Ms4a3*^{Cre}/*Rosa26*^{tdTomato} lineage tracing mice to determine inf-DC3 ontogeny. As detailed in point 12 we can imply that expanded inf-DC3 are not monocyte-derived as their labeling remains unchanged.

We are currently designing new tools, but their generation is in progress and will require significantly more time and will be the focus of a follow-up study.

Point 15

A cell intrinsic role for Notch2 in restraining inf-DC3 differentiation should also be assessed using mixed bone marrow chimeras.

This is indeed an important point. We have generated mixed as well as full BM chimeras to determine cell-intrinsic and cell-extrinsic drivers of inf-DC3 expansion. As shown AXL^+CD64^+ inf-DC3 expand in response to dysbiosis, while AXL^+CD64^- inf-DC3 expansion is a consequence of the genetic defect. A detailed reply to a similar comment is in Point 1.

Point 16

Related to this, loss of Notch signaling leads to impaired intestinal Th17 differentiation attributed to loss of IL23+ (either CD103+CD11b+ or CD103– CD11b+ cDC2s (Lewis et al. Immunity 2011, Ohara et al. JEM 2025). The loss of Th17 cells would likely lead to alterations in the intestinal microbiota that are independent of inf_DC3.

The authors should discuss these previous studies in more detail, and perform additional experiments to exclude a role for conventional DC2 subsets such as cDC2a, cDC2b or IL23+ cDC2s in dysbiosis and autoimmune phenotypes, for example using Clec9a-cre Rbpj flox mice which are available.

We are grateful for the comment and expanded the discussion in this regard, which now states: "The absence of *Notch2* within DCs would impair T_H17 priming, rendering gut epithelium less capable of controlling bacterial growth. Further, defective inf-DC3, and impaired expression of *Dnase1l3*, may favor the growth of enteropathogenic bacteria capable of forming biofilms, such as *C. rodentium*[4], explaining the increased susceptibility of this line to the pathogen."

As discussed in point 14, none of the available Cre lines would help us, as all the ones that we had considered would leave other interpretations open. More specifically the Clec9a-cre Rbpj flox would target NOTCH signaling pathways across all DC subsets including DC1, cDC2a cDC2b and DC3, given the expression of Clec9a in DC progenitors [5].

However, to better determine intrinsic defects related to the genotype we rederived the line, which would still have impaired Th17 priming ability and reduced IL-23 production in the absence of the identified pathobionts. As shown, and explained in detail in Point 1, only AXL^+CD64^- inf-

DC3 are expanded in cKO mice without any increased antibody titers and do not show autoimmune features. Only colonization by specific pathobionts leads to the expansion of $AXL^{+}CD64^{+}$ inf-DC3, and the appearance of high Ab titers and preclinical autoimmune symptoms. This subset is also expanding in *Notch2* competent WT mice in response to dysbiosis (Fig. R3 and new Fig. 7A-C), and precedes the appearance of autoantibodies (New Fig. S7S) and the described symptoms, supporting our hypothesis.

Point 17

CD11c-cre will also target CD11c expressing B cells which include populations of antibody producing B cells that have been implicated in autoimmune disease. Although the authors include an analysis of MZB cells, additional experiments should be performed to clarify whether the dysregulated antibody production observed in Notch2cKO mice is due to a cell intrinsic role of Notch2 in CD11c+ B cells.

As reported, deletion of *Notch2* in B cells results in the absence of MZB[6]. We have carefully looked at B cells in our mice, MZB cells are present and unchanged as shown by flow cytometry (Fig. S3H-I). Moreover, as shown in new Fig. S7P, colonic B cells in mixed bone marrow chimeras show equal donor contribution, suggesting that no specific defect affects this subset. Furthermore, we show that the response is directly linked to the dysbiosis as it is also happening in *Notch2*-sufficient WT mice.

Point 18

The authors perform a comprehensive scRNA-seq analysis of DCs within the spleen, mLN and sLN but the analysis of DC subsets in the intestinal lamina propria, the site of inflammation, is limited to flow cytometry characterization and relies on CD16/32 being a conserved DC3 marker across tissues. The authors state that they focused on inf_DC3 because of the pronounced increase in inf_DC3 in spleen of Notch2cKO mice, yet in mLN, there is only a modest increase in inf_DC3 in Notch2cKO mice (Fig. S2c). I was unclear whether the authors were proposing that the increase in inf_DC3 was responsible for ensuing dysbiosis. If so, this requires some additional onceptual and mechanistic explanation.

The scRNA-seq datasets (Fig. 2) were obtained at the beginning of the study and were instrumental in identifying the subset and the phenotype. In particular the lack of *Dnase1l3* prompted us to look at antibody titers. Based on the isotype specificity, suggestive of an anti-bacterial response, and given the association of DNASE1L3 with SLE and biofilm degradation, we focused our attention on the microbiome and the gut. The current manuscript now includes more detailed experiments on gut DCs, including time-course analysis, antibiotic treatment, rederivation, BM chimeras in new Fig. 7/S7, as well as scRNA-seq data in new Fig. 8/S8. We believe that the study in its current version is providing compelling evidence for the role of AXL^+CD64^+inf-DC3 in responding to the dysbiotic microbiome and promoting preclinical autoimmune symptoms. Unfortunately, current genetic tools do not allow for specific deletion of this subset, however we are currently designing new strategies to target them.

Point 19

The authors should perform scRNA-seq of DCs from WT vs Notch2cKO mice from the intestinal epithelium and lamina propria to confirm that inf_DC3 are increased at these sites and to determine if there are other relevant changes in DC populations that may be responsible for dysbiosis.

Following reviewer #2 suggestion, we optimized the isolation of colonic DCs and performed scRNA-seq on DC isolated from mLN and colon, obtained from WT, Red-*Notch2*^{cKO}, as well as dysbiotic WT and *Notch2*^{cKO} mice. The data is now complied in new Fig. 8 and S8.

Point 20

A second major point relates to the definitions of cDC2b/DC3 subsets. The study relies on the use of CD16/CD32 to distinguish cDC2b vs DC3. Although one study has described the existence of distinct cDC2b vs DC3 populations (Rodrigues et al. Immunity 2024), the original description of monocytic DC3 by Liu et al suggested that cDC2b and DC3 were synonymous. Integration of published datasets in which cDC2b or DC3 were described are needed to understand how these transcriptionally defined subsets relate to each other. In Fig. 2A and F, cluster 2 is annotated as a mixed DC3 and cDC2b cluster. If this is the case, these cells could be resolved by sub-clustering, similar to the analysis performed by Rodrigues et al., Immunity 2024.

The authors should perform subclustering to see if there are 2 transcriptionally distinct cell types present in cluster 2.

We fully agree with the reviewer that a certain degree of overlap exists in the characterization of cDC2b and DC3. CD16/32^{+}DC3 (Liu *et al*.) and cDC2b (Brown *et al*.) are as mentioned synonymous, yet in Rodrigues *et al*. (Immunity 2024) the cDC2b compartment is identified as CX3CR1^{+}CD16/32^{-}, which is distinct from CD16/32^{+}DC3 (Liu) and cDC2b (Brown). We applied the Rodrigues et al. more specific gating strategy that defines DC2b as CX3CR1^{+}CD16/32^{-} and DC3 as CX3CR1^{+}CD16/32^{+}.

We further show that additional markers allow for a refined segregation of DC3 into (i) AXL negative DC3 that are preserved in cKO mice and remain unchanged upon dysbiosis; (ii) AXL^{+}CD64^{-}inf-DC3 that are expanding in *Notch2* deficient mice; and (iii) AXL^{+}CD64^{+}inf-DC3 that expand upon dysbiosis. These data provide evidence for specific functional cues to each subset. Whether they represent specific states or independent lineages remains to be defined.

Nonetheless, differences in marker usage and subset definitions remain a broader issue within the dendritic cell field and affect nearly every newly described population. To improve the clarity, we are also providing readers with user friendly interfaces that enables browsing for favorite markers https://tuss-rox-lab.shinyapps.io/shinyapp/ (figure 2) and https://tuss-rox-lab.shinyapps.io/shinyapp-1/ (Figure 8).

Since we believed that sub-clustering would have generated a suboptimal analysis, we opted for the generation of new scRNA sequencing data. Based on cumulative evidence while DC3 appear as distinct clusters, cDC2b are transcriptionally comprised within cluster 6 that contains pDC-like cells and cDC2b (CD16/32 negative CX3CR1 positive cells). Further analysis of this this subset was beyond the scope of the current study.

Point 21

However, it is notable that there appears to be uniform Fcgr2b in this cluster but no Fcgr3 expression.

As shown in Fig. 2F, there is a difference in the RNA expression patterns of *Fcgr2b* and *Fcgr3*, as correctly pointed out by reviewer #2. Since the antibody detecting CD16/32 will bind to both these antigens, all cells expressing either Fc-receptor will be labeled in flow cytometry. We hope that this clarifies the concern.

Point 22

This is at odds with the authors proposed definition of DC3 as CD16/32+ cells and the flow cytometry characterization of cluster 2/cDC2b as CD16/32-CX3CR1+ cells and cluster 2/steady-state/WT DC3 as CD16/32+CX3CR1+AXL- cells.

DC3 were defined by flow cytometry labeling using CX3CR1 and CD16/32 as markers (Liu et al. Rodrigues et al.). We have adopted the same strategy as shown in Fig. 1F and included AXL and CD64 expression for further segregation. We hope that this is now clear.

Point 23

Since the study relies on CD16/CD32 as the distinguishing marker for DC3, prospective isolation and scRNA-seq analysis of Esam–CX3CR1+CD16/32+ DC3 vs Esam–CX3CR1+CD16/32– cDCb cells and integration with previous scRNA-seq datasets describing DC3 or cDC2b cells is warranted.

We are grateful for having raised this point that suggests lack of clarity. As stated above, surface marker expression analysis in myeloid cells is tricky and comparisons of gating strategies across studies not always straight-forward. We have tested multiple color combinations that would show a better resolution of the population of interest that was expanding upon dysbiosis and in *Notch2*^{cKO} mice. Collectively, *Axl* transcript and AXL expression seemed to ensure a good discrimination by transcript as well as by protein, warranting a good definition of our population of interest inf-DC3. Further we could identify CD64 as another marker expressed in mLN and colonic inf-DC3, that enabled us to characterize within DC3 the specific expansion caused by the genetic defect. Most importantly, the new scRNA-seq experiment supported our findings and to ensure that readers can identify other markers or determine improved gating strategies we are proving a user-friendly interface for selective marker assessment.

Point 24

In addition, the use of CD16/CD32 as a discriminatory cDC2b/DC3 marker has not been validated in inflammatory settings. It is possible that cDC2b upregulate CD16/CD32 in these contexts. Given the closely overlapping transcriptomes of cDC2b and DC3 which were only resolved into separate subsets based on fine sub-clustering and lineage tracing.

We fully agree with the reviewer and have as stated above generated new scRNA-seq data on DC isolated from mLN and colon, obtained from WT, Red-*Notch2*^{cKO}, as well as dysbiotic WT and *Notch2*^{cKO} mice. The data is now complied in new Fig. 8 and S8. This data set allows for direct comparison in eubiotic and dysbiotic conditions. As shown in new Fig 8L-M, a specific set of genes are induced following dysbiosis and include antigen processing and presentation as well as type I IFN response, which is a main driver of autoimmunity. Based on our scRNA-seq data, expression of FC receptors is not altered when comparing DC3 in rederived cKO and dysbiotic cKO mice (New Fig S8O).

Point 25

To define inf DC3 cells as DC3 rather than inf cDC2b cells, lineage tracing experiments are required to confirm that the cells characterized as inf DC3 are fate-mapped by Ms4a3-cre (Liu et al. Immunity 2023) rather than Clec9a-cre (Minutti et al. Nature Immunology 2024).

As explained in Point 12, 14 and 16, no Cre line is ideal for DC3 targeting and we are currently designing new strategies. However, as requested we obtained and analyzed *Ms4a3*-cre linage tracer mice. Briefly, labeling of AXL^{+}inf-DC3 is consistent with previous studies by Liu and Rodrigues and percent tdT positive cells does not change in response to dysbiosis (new Fig. S5W). Further we have included the authors of the *Ms4a3*-cre line to comply with their use.

Point 26

The definition of cDC2b/DC3 is particularly confusing in Fig. 1E where the authors identify only

cDC2a and DC3 clusters in cells isolated from WT mice but no cDC2b whereas cDC2b are present in Notch2cKO. This is at odds with previous studies from Reis e Sousa, Rudensky and Colonna labs which showed that cDC2b were present in the spleen at steady state. On what basis are the authors ascribing clusters 2 and 7 as DC3 rather than cDC2b? The scRNA seq analysis in Figure 2 gives a much more informative overview of cDC2 heterogeneity which renders Fig. 1 somewhat redundant and would perhaps be better as supplemental data to support Fig. 2.

We apologize for the confusion as Cluster 2 and 7 in WT are DC3 based on the expression of CD16/32. Cluster 5 and 14 express CX3CR1 and can therefore align with cDC2b, we have corrected the labeling in Fig. 1E and are grateful to the reviewer for the comment. We nevertheless believe that Fig. 1 is important as in contains relevant flow characterization and histology.

Minor comments:

Abstract: the authors state that NOTCH2 deficiency impairs the development of ESAM+ type 2a DC2s resulting in defective intestinal TH17 cells but it would be more accurate to state that NOTCH2 deficiency impairs the development of ESAM+ type 2a DC2s and intestinal CD103+CD11b+ cells (which have not been shown to represent cDC2a). Thus far the defect in TH17 cell differentiation has been linked to the IL23+ cDC2 population.

We have-modified the manuscript accordingly.
As shown in Fig S2C, all tissues lack cluster 4, which express *Esam* and *Tbx21* (Fig. S2B), as reported by Brown *et al*. 2019 and consistent with their identification as cDC2a, or *Notch2*-dependent DCs (Minutti *et al*. 2024). *Esam* is expressed also on LN migratory clusters 3 and 9 but these show a small reduction in the spleen and a small increase in peripheral LNs (Fig. S2C).
Within the intestinal tissues, cKO mice are devoid of cluster 1, 4 and 11, (new Fig. S8A) which express *Esam* and *Tbx21* (new Fig. S8B), consistent with being *Notch2*-dependent and their identification as cDC2a. Clusters 4 and 11 also express *Ccr7* (new Fig. S8B). Only cluster 1, shows CD11b transcripts and therefore likely aligns with the missing subset identified by Satpathy *et al*. 2013 as CD11b and CD103 positive cDC.

Within the introduction and Fig. 2A, the authors suggest that Rorc-expressing DCs are cDC2s. My understanding is that the definition of Rorc-expressing DCs as cDC2 stemmed from a previous study by Rudensky and this has been superseded by several studies which have shown that these cells are developmentally distinct from conventional dendritic cells, and only a subset of Rorc-expressing APCs express CD11b. In addition, their ontogeny has not been established. A recent study by Colonna's group suggested that they were CLP-derived and more closely related to ILCs. Without supporting evidence, the statements related to Rorc DC ontogeny and their inclusion in the cDC2 family should be omitted as this will only add to the considerable confusion in the field relating to this cell type.

We fully agree with this statement, therefore, as suggested, by the reviewer we have now omitted the statement.

Fig. 1I and S1M – despite the use of CD16/32 as a marker for DC3, the authors did not identify CD16/32 DC3 in WT mice. This requires further explanation.

In WT spleen the DC3 compartment is not present within the follicles, but appears to localize in proximity of the marginal zone area. We have modified the text so that this point is clear.

Reviewer #3

(Remarks to the Author)

Grozdan et al. revisit CD11cCre: Notch2fl/fl mice, exploring the possibility that the Notch2 deficiency not only results in the reported loss of ESAM+ cDC2a, but also additional alterations of the DC compartment. Detailed comparative analysis of mutants and controls, including comprehensive phenotype and transcriptome analyses revealed the accumulation of a cell population resembling a recently described DC3 subset. Using scRNASeq of sorted populations, the authors show that in particular in peripheral tissues, i.e. the intestine, these DC3-like cells cells show prominent expression of Axl and a pro-inflammatory gene signature, including TNF

and IL6 expression. Furthermore, DC of Cd11cCre: Notch2fl/fl mice displayed reduced expression of DNasel|3, which has previously been linked to immuno-pathology. Focusing on the intestine, the authors show that the DC3-like cells accumulate in GALT-like aggregates with T and B cells. Moreover, CD11cCre: Notch2fl/fl mice exhibit increased serum IgA and IgG2b/c serum titers and immune complex depositions in the kidneys. CD11cCre: Notch2fl/fl mice also show dysbiosis, and indeed, the authors show that co-housing of wt animals with mutants induces the accumulation of the DC3-like cells and the serum antibody changes. Moreover, they identify three dominant bacterial species (Parasutterella, Mucispirillum, Chlamydia) driving the alterations.

All data presented by the authors are of high standard, and the experiments are performed with rigor. The study contains a plethora of valuable information; however, concerns remain with respect to the general message and interpretation.

We are grateful for the very positive feedback and will try to address the concerns to the best of our capabilities.

Major points

Point 27

The authors suggest a causal link between DC dysfunction and systemic autoimmunity. Specifically, their title states that DC3-like cells are inducers of the phenotype, but this statement is problematic. To establish the causal role of DC3-like cells, one would arguably have to perform adoptive transfers.

We agree with this comment and have substantially extended our analysis by incorporating several new experiments that collectively corroborate our hypothesis. Based on the new data, we have revised the manuscript and updated the title accordingly. Major changes are highlighted in blue and multiple new panels have been added, along with two additional figures. To address this comment more specifically, we note that transfer experiments with DCs are highly inefficient and

difficult to interpret, particularly when using mature cells. Therefore, we performed a series of complementary approaches, including rederivation of *Notch2*^{cKO} (Red-*Notch2*^{cKO}) under SPF conditions, antibiotic treatment, time course analysis, and the generation of bone marrow chimeras.

Overall, our data indicates that the autoimmune response is triggered by colonization by opportunistic/pathogenic species. Furthermore, the presence and expansion of inf-DC3 closely correlates with the kinetic of microbial colonization and the induction of the immune responses in both WT and cKO mice. Transcriptional analysis highlights the inflammatory profile of the subset including their capacity for antigen processing and presentation. In addition, their localization within splenic follicles and within tertiary lymphoid structures in gut-associated tissues further supports their central role. The reduction of pathogenic species upon Abx use, correlates with the kinetic of DC3 contraction and parallels the decrease in autoantibody titers. Collectively these findings strongly support our hypothesis. While definitive proof would require specific depletion of the subset, such tools are currently unavailable. We have initiated the development of targeted strategies; however, these experiments are beyond the current study and will require extended time.

More specifically, Red-*Notch2*^{cKO} mice exhibit numbers of inf-DC3 comparable to WT mice in the absence of dysbiosis, as detailed in Point 1 (new Fig. 7G-J, **Fig. R1**). Autoantibodies correlate with the expansion of inf-DC3 following induction of dysbiosis (new Fig. S7S and 8A-B). Notably, antibiotic treatment reduces bacterial burden, leading to the contraction of inf-DC3 (new Fig. 7D-F, **Fig. R6**) and decreased serum autoantibodies titers (Fig. 6G). Further, inf-DC3 expansion follows microbial transfer also in WT mice, showing comparable cell numbers as in cKO mice (New Fig 7A-C, **Fig. R3**). Importantly, this expansion precedes the production of auto-antibodies (Fig. S7S).

Taken together, our data suggests that inf-DC3, expand in dysbiotic conditions in response to pathobionts in response to pathobionts, independently of genetic background, either directly or indirectly. Consistent with this, newly generated scRNA-seq data (new Fig. 8), show that inf-DC3 (cluster 9) are characterized by a strong inflammatory signature, induction of type I IFN-induced transcripts, as well as *Gbp* family members that were shown to activate the inflammasome in the presence of bacterial products. We strongly believe that to the best our capabilities we were able to address most of the concerns.

Point 28

Likewise, to evaluate whether DC3 emergence is intrinsic to Notch2-deficient pre-DCs, adoptive transfer of CD11cCre: Notch2fl/fl pre-DCs into WT recipients followed by assessment of DC3 development from the donor cells.

Since the number of cells is a limiting factor in transfer experiments, and the expansion of inf-DC3 is selective of the gut-associated tissue, transfer experiments are inapplicable or require a surgical expertise that we do not have. We have nevertheless addressed this point by generating mixed BM chimeras and analyzing Red-*Notch2*^{cKO}. Point 1 is a detailed reply, but briefly, AXL^{+}CD64^{-}inf-DC3 expand as a result of *Notch2* deficiency, while AXL^{+}CD64^{+}inf-DC3 expand in response to dysbiosis suggesting an cell-intrinsic effect of the first and a cell-extrinsic effect of the latter. Interestingly, the cell-intrinsic expansion of cKO-derived AXL^{+}CD64^{-}inf-DC3 occurs also in mixed bone marrow chimeras, suggesting enhanced proliferative capacity or improved survival in the absence of NOTCH2. This data may explain the increased susceptibility to enteropathogenic species given their strong inflammatory profile or explain the more severe phenotype upon DSS-induced colitis.

Point 29

The question arises whether the accumulation of DC3-like cells and dysbiosis is also observed in Notch2-deficient animals in other facilities or whether the observation is somewhat anecdotal.

We had asked some collaborators but were unable to identify someone who would have these mice. For this reason, we have rederived the line. In the absence of dysbiosis, inf-DC3 are not expanded (new Fig. 7G-J, **Fig. R1**). These new data allowed us to link inf-DC3 cell abundance with the dysbiosis and the colonization by pathobionts. How *Notch2*^{cKO} mice in our colony acquired these pathobionts while WT and other strains mice remained unaffected, remains enigmatic but might be related to their increased susceptibility to enteropathogenic infections, and inability to mount effective Th17 immunity.

Point 30

As it stands, the Notch2-deficient animals allowed the authors to reveal some potential interesting information on DC3-like cells, the Notch-control of DNAse expression, and on the effect of pathobionts on humoral immunity. However, none of these promising threads are pursued to a conclusive end.

Our significantly revised manuscript that now includes rederived *Notch2*^{cKO}, time-course experiments, bone marrow chimeras, antibiotic-treated mice, as well as a new single-cell RNA sequencing dataset generated from tissue samples of WT, dysbiotic WT, rederived cKO, and dysbiotic cKO mice (new Fig. 7 and 8).
Together, these integrated datasets not only strengthen our conclusions but also provide mechanistic insight into the development of autoimmune pathology. We are confident that the revised manuscript directly addresses the concerns raised by all reviewers and all major points of criticism. We have further adjusted the title based on the new findings. We have extended our discussion to include more critical points or future direction that we hope will intrigue the broad audience of the journal.

The complexity of autoimmune pathogenesis is far from being resolved however we are very excited to be able to provide some significant cues and advancement to the field, that will enable us and the larger community of immunologists, microbiologists and clinicians to pursue these avenues and contribute with their expertise.

Point 31

It also remains unclear which of the DC alterations are due to cell-intrinsic effects of the Notch deficiency and which are secondary effects due to environmental changes.

We have addressed this concern extensively by rederiving the line, by generating mixed bone marrow chimeras, and by treating dysbiotic mice with broad spectrum Abx as explained in point 1, 2, and 9.

Briefly, we prove that the expansion of AXL^+CD64^+inf-DC3 occurs in response to dysbiosis, while AXL^+CD64^-inf-DC3 show a *Notch2*^{cKO}-intrinsic expansion in addition to the lack of cDC2a. Furthermore, the scRNA-seq on WT, dysbiotic WT, rederived cKO, and dysbiotic cKO allowed us to determine the specific transcriptional profile and delineate genetic and environmental effects (new Fig. 8). We also included a table which highlights DEG across all DC clusters in dysbiotic WT and/or cKO mice (new Fig. S8O).

Point 32

It would be good to highlight which of the DC populations express Notch2 and In which DC populations are direct Notch targets such as Hes1 affected (see Fig. 2E) ? Mixed BM chimeras could further help dissect intrinsic effects.

The expression of *Notch2* is broad across all DC subsets and therefore multiple subsets are affected by *Notch2* deficiency, as shown by impaired *Dnase1l3* expression across all DC subsets (Fig. 2L). We have further included in our study single and mixed BM chimeras, that is now displayed in Fig. 7K-O, S7N-R. Further we have generated new scRNA-seq data analysis on DC isolated from mLN and colon, obtained from WT, Red-*Notch2*^{cKO}, as well as dysbiotic WT and *Notch2*^{cKO} mice. These datasets ensure the possibility to directly compare intrinsic and extrinsic effects for WT and cKO mice under eubiotic and dysbiotic conditions. We have also generated a user-friendly interface to enable readers to explore expression profiles across subsets, and conditions.

Point 33

The fact that co-housing, i.e. the mere microbiota transfer, results in an increase of Axl+ DC3 cells, alongside elevated autoantibody levels, challenges the notion of a causal link between DC3 and dysbiosis/autoimmunity. Would the DC3 expansion occur in germ-free or broad-spectrum antibiotic-treated CD11cCre; Notch2fl/fl mice?

This was an important aspect, and we have therefore rederived the line and could show that the expansion of AXL^+CD64^+inf-DC3 occurs in response to dysbiosis, while AXL^+CD64^-inf-DC3

show a *Notch2*^{cKO}-intrinsic expansion in addition to the lack of cDC2a. Rederivation in germ-free conditions was not possible in our facility. We however, treated both dysbiotic WT and cKO mice with broad spectrum antibiotics. As shown in new Fig. 7D-F and **Fig. R6,** the reduction in microbiome biomass contracts inf-DC3, lowers autoantibody titers, and depletes the reads specific for the identified pathobionts.

Also, in the context of antibiotic treatment, only *Notch2*^{cKO} retained elevated numbers of AXL^{+}CD64^{-}inf-DC3 further strengthening the genetically-driven expansion of this subset (new Fig. S7E-F, H), in line with the Red-*Notch2*^{cKO} and BM chimera data.

Point 34

Is also the DNasel|3 deficiency observed under dysbiosis in wt mice or is it strictly linked to the Notch2 deficiency?

Based on our new scRNA-seq data analysis, expression of DNASE1L3 is dependent on *Notch2*, such that KO mice are not expressing it at steady-state, nor in response to pathobionts.
WT DCs express this DNase across all lymphoid tissues tested (spleen, mLN and sLN). Dysbiosis leads to increased expression only in the colon but not in mLNs DC of WT mice as now shown in new Fig. S8Q.
Further, we generated DNASE1L3-NeonGreen-P2A-hCD4 mice and induced dysbiosis in these mice. In this mice expression of *Dnase1l3* can be monitored by NeonGreen and hCD4. As shown in **Fig. R4** in response to reviewer #1 in point 5, dysbiosis leads to induction of the gene as tracked by the reporter gene.

Point 35

Peripheral tissue resident DC3 populations remain less well characterized, particularly with regard to lineage tracing with a decisive discriminating factor being that these cells are not derived from monocytes. While splenic and BM DC3 populations were identified using established gating strategies, it is unclear whether these criteria are valid for intestinal DC, whose marker expression often differs by tissue. Notably, the use of CD64, a common intestinal macrophage marker

(Tamoutounour et al.) to define DC3 is concerning. The observation that the CD64+ DC3 accumulation is abrogated in CCR2-deficient mice, and that the DC3 increase compensates for the DC2 loss in Flt3l KO mice, would support monocyte-derivation. Likewise, Klf4 is also required for monocyte development (Kurotaki et al.)

Their assignment to the DC3 lineage is based on the entire transcriptional profile and co-expression of multiple key surface markers. However, we do agree with the reviewer that discrimination between DC and monocyte-derived DCs is challenging under inflammatory conditions. For this reason, we have used the previously suggested marker CD26 as the one allowing for discrimination between inflammatory monocytes and DCs, as elegantly shown by M Guilliams *et al*. 2016 and Bosteels *et al* 2020. Nevertheless, to better solve this question we have imported some *Ms4a3*^{Cre}*Rosa26*^{tdTomato} mice to look at the ontogeny of the DC3 compartment in these mice. Inf-DC3 labeling is intermediate, and the frequency of labeled cells does not change in dysbiotic mice, while monocyte labeling is close to 100%. This excludes that inf-DC3 are of monocytic origin, even under inflammatory conditions (new Fig. S5W) as detailed in point 12. Further studies that are beyond the scope of this report, are required to establish the direct precursor/s of this subset.

Point 36

Thorough clarification of the identity of colonic DC3, including Axl-expressing populations, would require single-cell RNA-seq analysis of colonic myeloid cells, including both DC and macrophages, as was done in spleen and LNs.

As this was a major concern also for reviewer #2 we decided to generate scRNA-seq data on DC isolated from mLN and colon, obtained from WT, Red-*Notch2*^{cKO}, as well as dysbiotic WT and *Notch2*^{cKO} mice. A detailed analysis is shown in Fig. 8 and S8.

Point 37

Furthermore, administration of neutralizing anti-CSF1R antibodies may help distinguish intestinal DC3 from macrophages based on differential depletion (see Nonaka et al. 2025).

We had also reasoned about the possibility of using anti-CSF1R antibodies. However, as several subsets express this marker, we believed it would not add more clarity to the presented data, in particular it would only be complementary to the results obtained with CCR2-deficient mice.

Point 38

Related to the above, the discussion states: "DC3 were recently identified as a distinct DC subset that is transcriptionally similar to, but developmentally distinct from monocyte-derived DC." However, to our knowledge, no study has definitively demonstrated a developmental distinction between DC3 and monocyte-derived DCs. If such literature exists, it should be cited.

We have now added the most relevant citation however for clarity we have here included a more detailed explanation for the cited work:

To our knowledge, in the study by Bosteels *et al*. Immunity 2020, CD26 was shown to segregate DCs from Mo-DCs. And for this reason, we have included CD26 in all datasets and plots. Further, Liu *et al*. Immunity 2023 conclude that these cells arise from a distinct progenitor rather than monocytes: "Using fate mapping models, single-cell RNA sequencing and adoptive transfer, we identified a lineage of murine CD16/32+CD172a+ DC3, distinct from DC2, arising from Ly6C+ monocyte-DC progenitors (MDPs) through Lyz2+Ly6C+CD11c- pro-DC3s, whereas DC2s develop from common DC progenitors (CDPs) through CD7+Ly6C+CD11c+ pre-DC2s. Corresponding DC subsets, developmental stages, and lineages exist in humans. These findings reveal DC3 as a DC lineage phenotypically related to but developmentally different from monocytes and DC2s."

Minor points

It is well established that gut DC require retinoic acid for proper differentiation and function, with ALDH enzymes playing a key role in RA synthesis, thereby promoting Treg induction and IgA

production (Erkelens et al., 2017). The authors should analyze expression of Aldh genes in intestinal DCs of CD11cCre; Notch2fl/fl mice.

We are grateful for this input and analyzed colonic Aldh expression on the newly generated scRNA-seq dataset. As shown for the colon (Fig. R9), Aldh1a2 is the only Aldh gene detected The gene is only expressed at low levels, but appears to increase in response to dysbiosis, in both genotypes. We do not observe any difference between WT and cKO DCs.

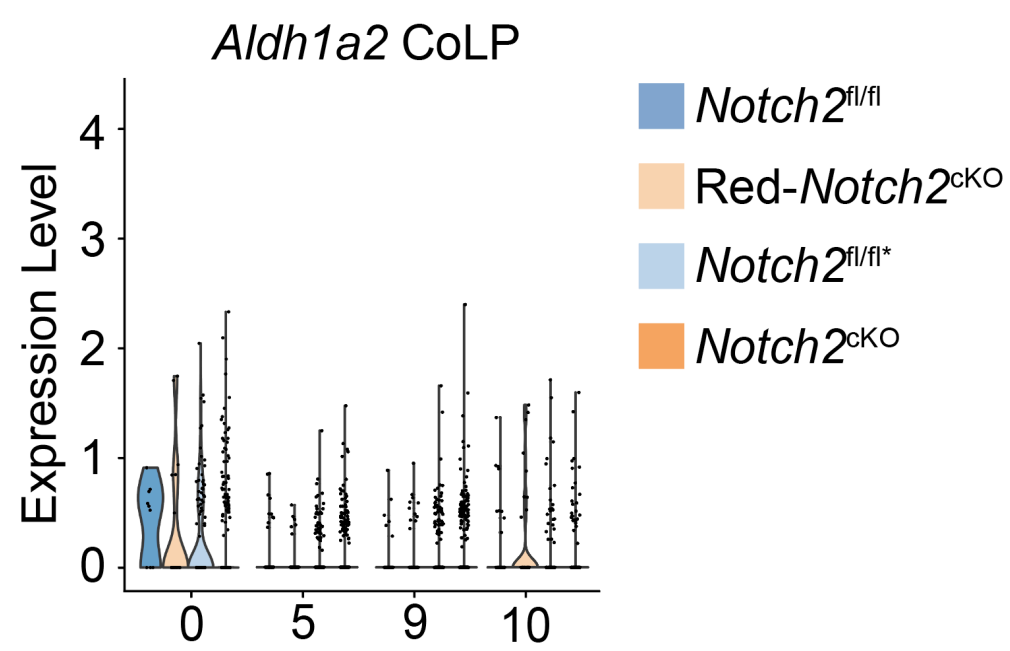

Fig. R9. Colonic *Aldh1a2* expression levels across major clusters, by scRNA-seq.

We observe expression of this gene in mLN, with the highest expression in cluster 12 followed by cluster 3, but no difference by genotype or dysbiosis (Fig. R10).

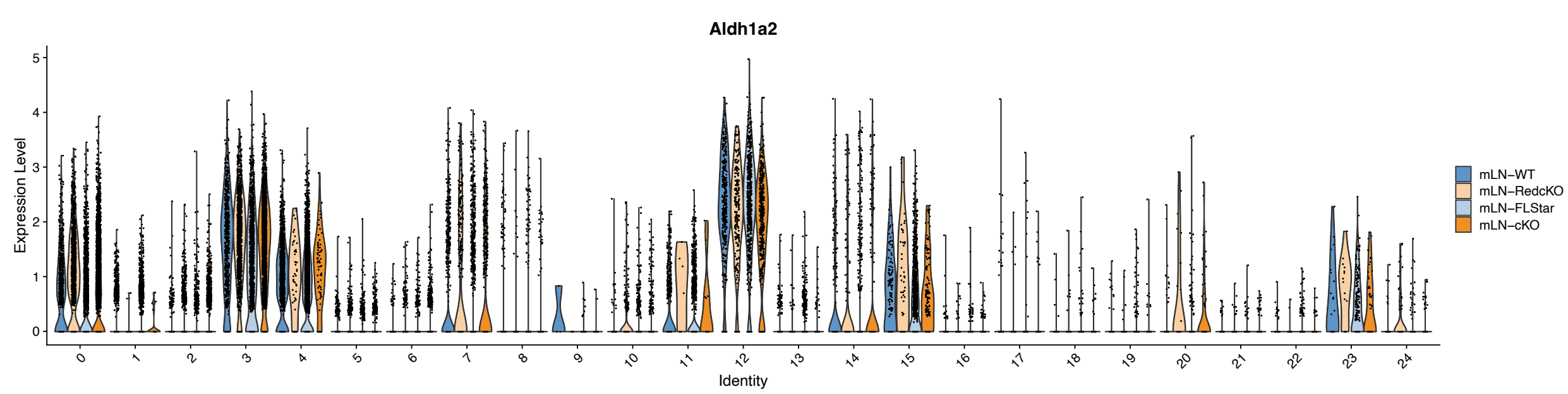

Fig. R10. mLN *Aldh1a2* expression levels across all clusters.

Additionally, treatment of these mice with exogenous RA may clarify whether RA supplementation can ameliorate autoimmune-like pathology.

As there was no difference in gene expression, we believed that the investment of time in optimizing and obtaining animal study approval for the suggested experiment was not worth as this was considered a minor point.

IL-23 is a known driver of Th17-mediated autoimmune diseases such as IBD, RA, and SLE, and IL-23p19 knockout mice show reduced disease severity in colitis models (Yen et al., 2006). that CD11cCre; Notch2fl/fl mice were reported to lack IL-23-producing DC (Ohara et al., 2024). The autoimmune phenotype associated with dysbiosis in Notch2-deficient mice suggests hence a potentially mechanism distinct from classical IL-23–Th17-mediated IBD, a point deserving further discussion.

We agree with this comment and have addressed concern as detailed in point 16

- Fig. 1I, 5J, S5I

Microscopy images indicating DC3 localization are difficult to interpret. Including DAPI or other structural markers to visualize tissue context and simplifying the color combination would significantly improve clarity.

In order to improve visualization, we have now included the original image without magnification (new Fig. S1M) and included CD169 to help localize follicles and CD11c to help localize DCs. In cKO mice 70% CD11c expressing cells in the spleen are DC3, and those show a clear localization within follicles based on CD169 expression (Fig. 2I). We opted for markers that would enable a better characterization of DC instead of including structural markers, given the complexity of DC subsets. Fig. S1N includes individual colors to help the readout. We hope that this improvement and explanation will satisfy this minor concern.

For Fig. 5 a new panel (S5M) was included to help provide tissue context and orientation.

- Fig. 1G,H How were DC3 defined for sorting for bulk RNAseq ? Show gates and add info

This information is provided in the methods and now included in the legend. DC3 are defined as $Lin^{-}CD11c^{+}MHC\text{-}II^{+}XCR1^{-}CD16/32^{+}ESAM$.

- Fig. 4K

The claim that Akkermansia reads are reduced by co-housing lacks sufficient statistical support. Please include appropriate statistical analysis.

We have now included the appropriate statistical analysis, performing a paired two-tailed t-test.

- Fig. 5H, I, N–P

To better assess whether DC3 changes are statistically significant compared to other cell types, frequencies should be expressed as a proportion of total CD45+ cells rather than cDC2 alone, and compared with cDC2a and cDC2b using appropriate statistical tests.

We agree with this comment and have decided to include absolute numbers. The new analysis is shown in new Fig. S5I-L.

- Fig. 6L, M

Please include body weight curves to complement the DSS-induced colitis data.

This is now shown in new Fig. S6P

- Fig. 6, S6.

Please specify the starting age and duration of co-housing in the methods or figure legend.

We have included this information in figure legend and the Method section.

1. Lacey, K.A. et al. Secreted mammalian DNases protect against systemic bacterial infection by digesting biofilms. *J Exp Med* **220** (2023).
2. Villani, A.C. et al. Single-cell RNA-seq reveals new types of human blood dendritic cells, monocytes, and progenitors. *Science* **356** (2017).
3. See, P. et al. Mapping the human DC lineage through the integration of high-dimensional techniques. *Science* **356** (2017).
4. Lackraj, T. et al. Novel antimicrobial peptide prevents C. rodentium infection in C57BL/6 mice by enhancing acid-induced pathogen killing. *Microbiology (Reading)* **162**, 1641-1650 (2016).
5. Schraml, B.U. et al. Genetic tracing via DNGR-1 expression history defines dendritic cells as a hematopoietic lineage. *Cell* **154**, 843-858 (2013).
6. Saito, T. et al. Notch2 is preferentially expressed in mature B cells and indispensable for marginal zone B lineage development 1. *Immunity* **18**, 675-685 (2003).

Point by point revision 2

First, I would like to thank all reviewers for their careful evaluation of our manuscript and for the constructive comments.

Reviewer #1

(Remarks to the Author)
The authors have done an excellent job addressing all of my comments and points. I have no further remarks and fully support the publication.

We appreciate the positive feedback by reviewer #1.

Reviewer #2
(Remarks to the Author)
The authors have undertaken a substantial series of additional experiments to address the prior comments, including fecal transfer studies, rederivation of cKO mice, and detailed phenotypic analysis of intestinal DC populations by flow cytometry and scRNA-seq. Collectively, these revisions provide important new insight into the sequence of events leading to dysbiosis and autoantibody production, as well as the associated changes in DC compartments across disease progression. I have only two remaining comments regarding the revised manuscript.

We thank reviewer #2 for acknowledging the undertaken work to address all comments to the best of our capabilities.

The authors identify a subset of CD64–CD16/32+ “inf DC3” cells that is expanded in rederived cKO mice and propose that this population is causally involved in the initiating dysbiosis marked by outgrowth of three pathobionts. However, in the absence of genetic tools to selectively target this subset, or candidate effector molecules such as Dnase1l3, it remains unclear why this population, rather than the cDC2a subset that is also reduced in cKO mice, should be considered

the primary driver of dysbiosis. An alternative interpretation is that loss of cDC2a initiates dysbiosis.

We agree with Reviewer #2 that, in the absence of genetic tools to selectively target the CD64⁻CD16/32⁺ DC3 population or candidate effector molecules such as *Dnase1l3*, a definitive causal link between this subset and the initiation of dysbiosis cannot be established. An alternative explanation, such as the loss of cDC2a as a primary driver of dysbiosis, is also plausible and important to consider. We note that this possibility is already reflected in our manuscript. In the abstract, we state that "*NOTCH2 deficiency impairs cDC2a development, leading to defective intestinal TH17 responses and increased susceptibility to enteropathogenic bacteria, which in turn contributes to the development of persistent dysbiosis*". This framing acknowledges that loss of cDC2a may represent an initiating event.

In addition, in the conclusion of Fig. 7, we propose that the expansion of CD64⁻ DC3 cells occurs in the context of this altered immune landscape. To further clarify this point, we revised the text to explicitly reflect a multifactorial model: **"This cell-intrinsic expansion, together with the reported cDC2a-dependent TH17 deficiency, could explain the increased susceptibility in deficient mice."**

Taken together, we favor a model in which both the loss of cDC2a (impacting TH17-mediated barrier immunity) and the expansion of inflammatory DC3 contribute to disease susceptibility, but we agree that our current data do not allow us to assign primary causality to either population.

I still find the definition of DC3 somewhat confusing. The DC3 subset was defined by the Ginhoux lab and the same lab subsequently reported that these cells were derived from monocyte progenitors, implying that DC3 represents a monocyte-derived DC population.

We agree that the definition and ontogeny of DC3 remain an area of active discussion, which may contribute to the reviewer's concern. Early work from the Ginhoux lab described DC3 as a distinct subset with features overlapping monocyte-derived cells, which has led to the interpretation that DC3 are monocyte-derived. However, more recent data refine this view.

Specifically, Liu *et al*. (Immunity, 2023) showed that DC3 do not to arise from classical monocyte progenitors (cMoP), but instead from a Ly6C⁺ monocyte–dendritic cell progenitor (MDP) that

retains dendritic cell potential. In that study, fate-mapping demonstrated that cMoP and circulating monocytes are labeled close to 100%, whereas CDP and early pre-DC subsets remain minimally labeled (<5%). In contrast, Ly6C+ MDP, pro-DC3, and mature DC3 show intermediate labeling (~20–40%), supporting a developmental pathway distinct from classical monocytes and DCs. Importantly, our own fate-mapping results are highly consistent with these findings. Both at steady state and under dysbiotic conditions, the "inf DC3" population exhibits intermediate labeling (~30–40%), closely matching the labeling kinetics reported for DC3 derived from Ly6C+ MDP rather than from fully committed monocyte progenitors.

We revised the manuscript to clarify this point in the discussion and avoid potential misinterpretation, emphasizing that the DC3 population studied here is unlikely to represent classical monocyte-derived DCs, but instead aligns with the recently described MDP-derived DC3 lineage.

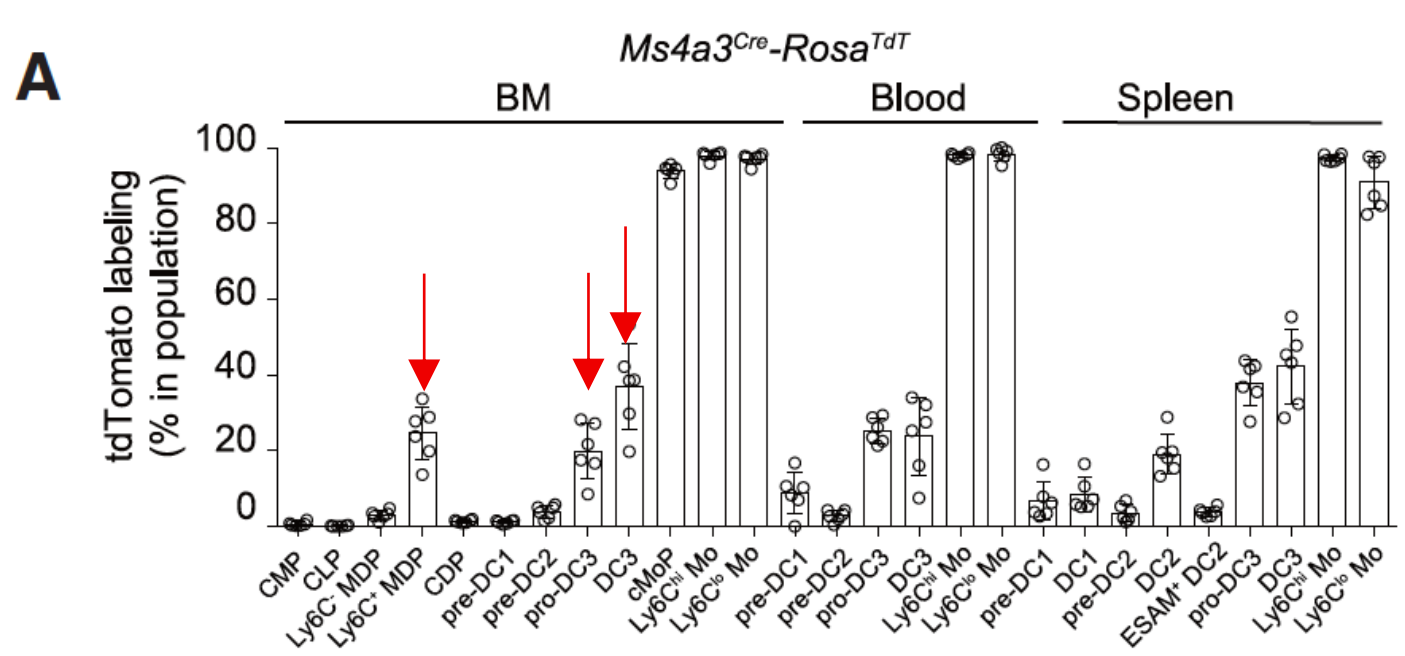

From Liu et al.

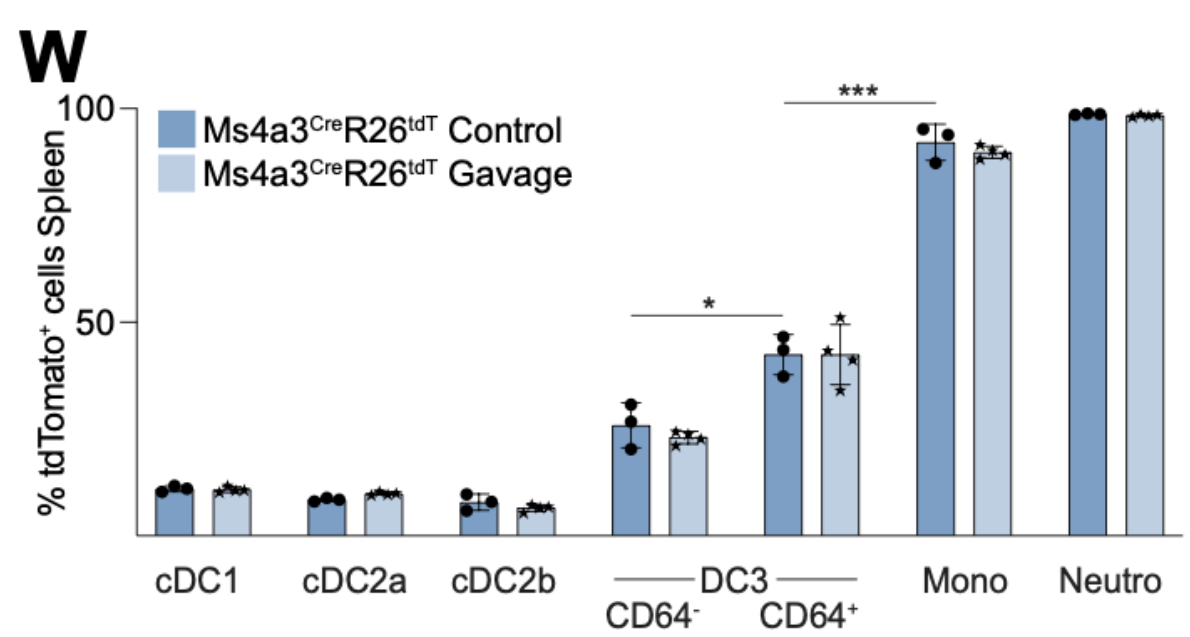

Cvijetic et al Extended Data Figure 6k.

Ms4a3-cre is a very efficient lineage tracer. Thus, the incomplete Ms4a3 fate mapping observed in CD16/CD32+ CD11b+ DCs likely reflects the fact that CD16/CD32 does not discriminate monocyte derived DC3 from CDP derived cDC2b, as reported by Nguyen et al. (PMID: 41272166). It would therefore seem more accurate to describe this population as CD16/32+ cDC2b/DC3.

We thank the reviewer for raising this important point regarding the interpretation of Ms4a3-Cre fate mapping and the nomenclature of the $CD16/32^+$ $CD11b^+$ DC population.
We agree that Ms4a3-Cre is a highly efficient lineage tracer and that incomplete labeling within this compartment likely reflects underlying heterogeneity rather than technical inefficiency. In this context, CD16/CD32 expression alone does not fully discriminate between monocyte-associated DC3 and CDP-derived cDC2b, as also highlighted by Nguyen et al. (PMID: 41272166). We therefore agree that this population may encompass both cDC2b- and DC3-like cells.
As in any lineage tracing approach, partial labeling cannot definitively resolve ontogeny and may reflect either heterogeneous progenitor contributions or the complexity of intermediates such as the $Ly6C^+$ monocyte–DC progenitor (MDP), which retains the potential to generate both monocytes and DCs. **We now explicitly acknowledge this limitation in the revised manuscript discussion.**
We carefully considered alternative nomenclature, including a broader cDC2b/DC3 designation. However, we opted to use “DC3” because our fate-mapping data (~30–40% labeling) closely align with the labeling profile reported for DC3 and their progenitors in Liu et al. (Immunity, 2023), supporting a relationship to the MDP-derived DC3 lineage. In contrast, the study by Nguyen et al. relies on Clec9a-Cre fate mapping without parallel Ms4a3-Cre analysis, which makes direct comparison to our system challenging. Notably, the authors themselves highlight these discrepancies and propose multiple interpretations regarding the relationship between DC3, cDC2 subsets, and progenitor origins.
Importantly, beyond fate mapping, our population expresses defining features of bona fide dendritic cells, including Zbtb46 and CD26, the latter of which has been shown (Bosteels et al.) to distinguish DCs from monocyte-derived cells even under inflammatory conditions. These data further support the interpretation that this population is not composed of classical monocyte-derived DCs. **To reflect these considerations, we have revised the manuscript to (i) cite**

Nguyen et al. (ii) clarify the potential overlap between cDC2b and DC3 populations, and (iii) explicitly acknowledge that the ontogeny and nomenclature of these subsets remain unresolved in the discussion.

Overall, we agree that the DC2/DC3 landscape is complex and that resolving lineage relationships will require improved markers and next-generation genetic tools. A unified framework across the field will be essential to address these questions moving forward.

Do the authors observe differences in the proportion of Ms4a3 fate mapped cells within CD64+ vs CD64– CD16/32 DC3 populations?

As shown revised Extended Data Figure 6k the % labeling of both $CD64^{+}$ and $CD64^{-}$ subsets is shown.

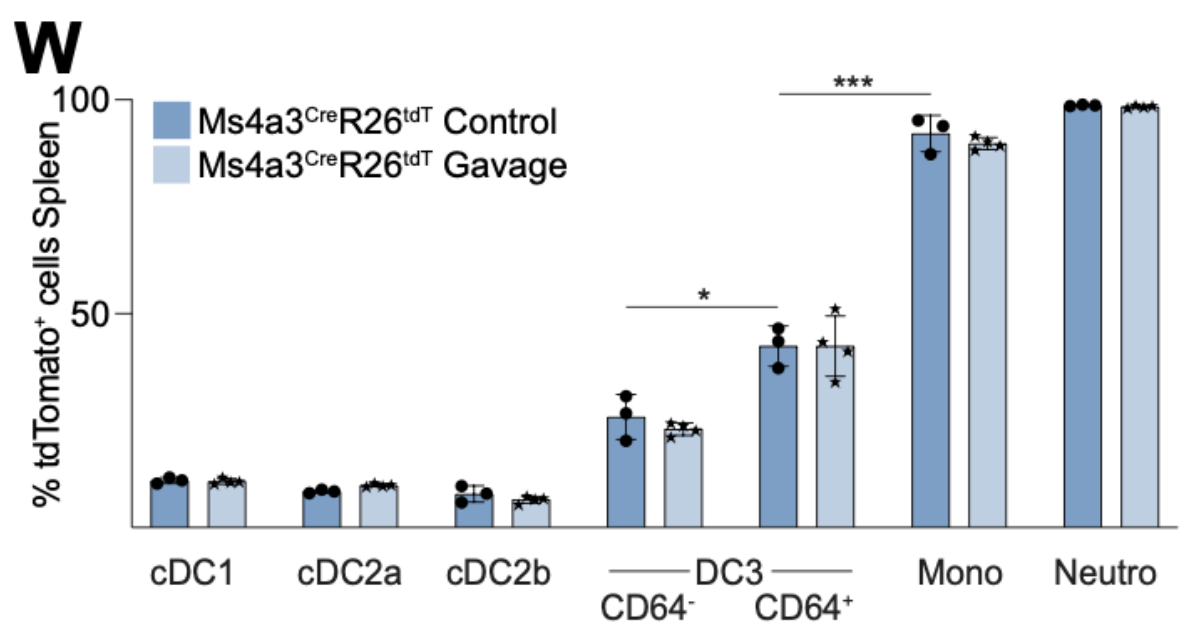

Cvijetic et al Extended Data Figure 6k

Reviewer #3

(Remarks to the Author)

The manuscript remains complex but the clarification of causality is a clear improvement.

The authors have responded to most of our points, but some issues remain and are listed below.

We thank reviewer #3 for the positive feedback on our revised draft.

Related to point #28

We understand the technical challenges of pre-DC transfer experiments, but it is problematic to

dismiss lineage testing solely on the ground of limited cell numbers. Flt3L-Fc could be used to expand DC progenitors and enable adoptive transfer experiments. Although the mixed BM chimera data support a competitive advantage and/or enhanced intrinsic survival or proliferation of Notch2-deficient cells, they do not directly demonstrate that DC3 are derived from CD135+ DC progenitors. Defining a cell type requires not only phenotypic characterization but also clarification of its developmental origin.
(https://www.sciencedirect.com/science/article/pii/S1074761320301631)

We thank the reviewer for this important point and would like to clarify that it was not our intention to dismiss adoptive transfer approaches. We agree that such experiments can provide valuable insight into developmental origin. However, the challenges extend beyond limited cell numbers.
A key limitation is the uncertainty regarding the appropriate progenitor population to isolate. Based on current lineage tracing data, DC3 are thought to arise from Ly6C$^+$ monocyte–dendritic progenitors (MDP), which retain the capacity to generate both monocytes and dendritic cells. As a result, adoptive transfer of MDP would not allow us to discriminate whether the resulting population represents a bona fide DC lineage or a monocyte-derived population. Thus, even if technically feasible (e.g., via Flt3L-Fc expansion), such experiments would not definitively resolve the ontogeny of the inf-DC3 population.
To address this question within these constraints, we instead employed complementary approaches. Mixed bone marrow chimera experiments demonstrate a cell-intrinsic competitive advantage of Notch2-deficient cells, while Ms4a3-Cre fate mapping provides insight into lineage contribution. Together, these data argue against a purely monocyte-derived origin.
In addition, we incorporated phenotypic criteria aligned with the definition of bona fide dendritic cells. Notably, the inf-DC3 population expresses CD26, which has been shown (Bosteels et al.) to reliably distinguish conventional DCs from monocyte-derived DCs even under inflammatory conditions in epithelial tissues such as the lung.
We fully agree with the reviewer that the precise DC precursor of inf-DC3 remains to be formally defined. **We now explicitly acknowledge this limitation in the revised manuscript discussion**. Nevertheless, based on the combination of fate mapping, competitive chimeras, and phenotypic characterization, our data support the interpretation that inf-DC3 belong to the DC3 lineage as described by Liu et al. (Immunity, 2023).

Related to point #35

CD26 has been proposed as a marker to distinguish DCs from macrophages under steady-state conditions, but to our knowledge, it has not been established as a reliable marker of intestinal DCs under inflammatory conditions. Therefore, when defining a DC3 population in the intestine, where such populations have been only minimally characterized, caution is required when assigning these cells as macrophages or DCs, particularly with respect to their developmental origin.

Moreover, the Ms4a3 fate-mapping data do not conclusively exclude a monocytic origin. It is known that, in Ms4a3 fate-mapping mice, the labeling efficiency of tissue macrophages is lower than that of circulating blood monocytes. Therefore, simply comparing the labeling frequency of the putative inf-DC3 population with that of blood monocytes does not provide sufficient evidence that these cells are not monocyte-derived.

We thank Reviewer #3 for highlighting these important considerations regarding marker reliability and lineage interpretation.

We agree that CD26 has been primarily validated as a discriminator between dendritic cells and macrophages under steady-state conditions, and that its applicability in the intestine under inflammatory settings is less well established. We therefore interpret CD26 expression cautiously and do not rely on it as a sole criterion. Instead, we use it in combination with additional features, including Zbtb46 expression, fate-mapping approaches, and competitive bone marrow chimera experiments, to support the classification of this population within the dendritic cell compartment.

We also agree that Ms4a3-Cre fate mapping does not formally exclude a monocytic origin. As the reviewer correctly points out, as labeling efficiencies can differ between circulating monocytes and tissue-resident populations, and partial labeling may reflect either developmental heterogeneity or limitations of the model. **We now explicitly acknowledge this caveat in the revised manuscript.**

To address lineage as rigorously as possible with currently available tools, we combined Ms4a3-Cre fate mapping with mixed bone marrow chimeras and phenotypic characterization. While none of these approaches alone is definitive, their convergence argues against a purely monocyte-derived population and supports alignment with the DC3 lineage described in recent studies.

We fully agree that defining developmental origin is an important objective. However, given the current limitations of available genetic tools and the unresolved complexity of DC2/DC3 ontogeny, we have focused this study primarily on the functional contribution of this population in the context of dysbiosis. **We have revised the manuscript to more clearly delineate this scope and to explicitly acknowledge the remaining uncertainty regarding lineage.** We anticipate that future studies using improved genetic models and more specific markers will be required to definitively resolve the ontogeny of these cells.

The statement **"These findings reveal DC3 as a DC lineage phenotypically related to but developmentally different from monocytes and DC2s."** is too strong and should be modified.

We thank the reviewer for this comment and agree that statements regarding the developmental origin of DC3 should be made with appropriate caution. We would like to clarify, however, that the statement referenced by the reviewer does not appear in the manuscript itself, but was included in our point-by-point response citing Liu et al.
In the manuscript, we refer to Liu et al. more cautiously, stating: "DC3 were recently identified as a distinct DC subset that is transcriptionally similar to, but developmentally distinct from monocyte-derived DC." Importantly, we do not make definitive claims regarding ontogeny based on our data. On the contrary, when discussing Ms4a3 fate-mapping results, we explicitly acknowledge this limitation: "However, these data did not allow definitive conclusions regarding the developmental origin of this subset."
To avoid any potential misunderstanding, we further revised the text to ensure that the distinction between published interpretations and our own conclusions is fully clear, and that all statements regarding DC3 ontogeny remain appropriately qualified.

Related to point #36
It is known that macrophages also express Axl (https://www.science.org/doi/10.1126/science.aai8132). This has to kept in mind when proposing Axl as marker for identifying DC3.

We thank the reviewer for this important comment. We agree that Axl expression is not restricted to DC3 and has also been reported in macrophages, including in the study cited (PMID: 25594431 and PMID: 28495875). This highlights that Axl, like other scavenger receptors, should not be used as a standalone marker to define this population. In our study, we therefore interpret Axl expression in combination with additional phenotypic and transcriptional features, rather than as a definitive identifier. Within this framework, Axl remains a useful marker that contributes to the identification of the DC3-like population.

We acknowledge that we did not cite the referenced study and included it in the revised manuscript, along with clarification of the limitations associated with Axl expression.

Related to point #36

The results obtained in CCR2-deficient and Flt3l-deficient mice would lead many readers to interpret the DC3 population discussed by the authors as being derived from CCR2-dependent monocytes, rather than as an Flt3L-dependent DC-lineage population.

We thank the reviewer for this important comment and agree that, without additional clarification, the results obtained in CCR2- and Flt3L-deficient settings could be interpreted as supporting a monocytic origin of the DC3 population. We apologize for the lack of clarity in the original text. Importantly, CCR2 deficiency does not exclusively affect monocyte egress. **As shown by the group of Reis e Sousa (Cell Reports, 2023), CCR2 also contributes to the mobilization of dendritic cell progenitors from the bone marrow.** Thus, the reduction observed in CCR2-deficient mice cannot be interpreted as evidence for a purely monocyte-derived origin, as both monocytes and DC progenitors may be impacted. **We revised the manuscript to make this point more explicit in both Figure 5 and discussion.**

We agree that the lack of a strong reduction in Flt3L-deficient mice could raise questions regarding their classification within the DC lineage. However, several lines of evidence argue against a classical monocytic origin. First, in Ms4a3-Cre fate-mapping mice, labeling of the inf-DC3 population remains partial (~30–40%) and does not increase under dysbiotic conditions, whereas monocytes are labeled close to 100%. This suggests that the expansion of this population is not

driven by recruitment or accumulation of monocyte-derived cells. Second, the population expresses canonical DC features, including Zbtb46 and CD26 (as shown by Bosteels et al. to distinguish bona fide DCs from monocyte-derived cells in inflamed epithelial tissues). **In addition, we were able to generate this population *in vitro* from conventional DC cultures in the presence of Flt3L, supporting a relationship to the DC differentiation pathway (Figure R1). This data is now included in the manuscript (Extended Data Fig. 6j) and discussed.**

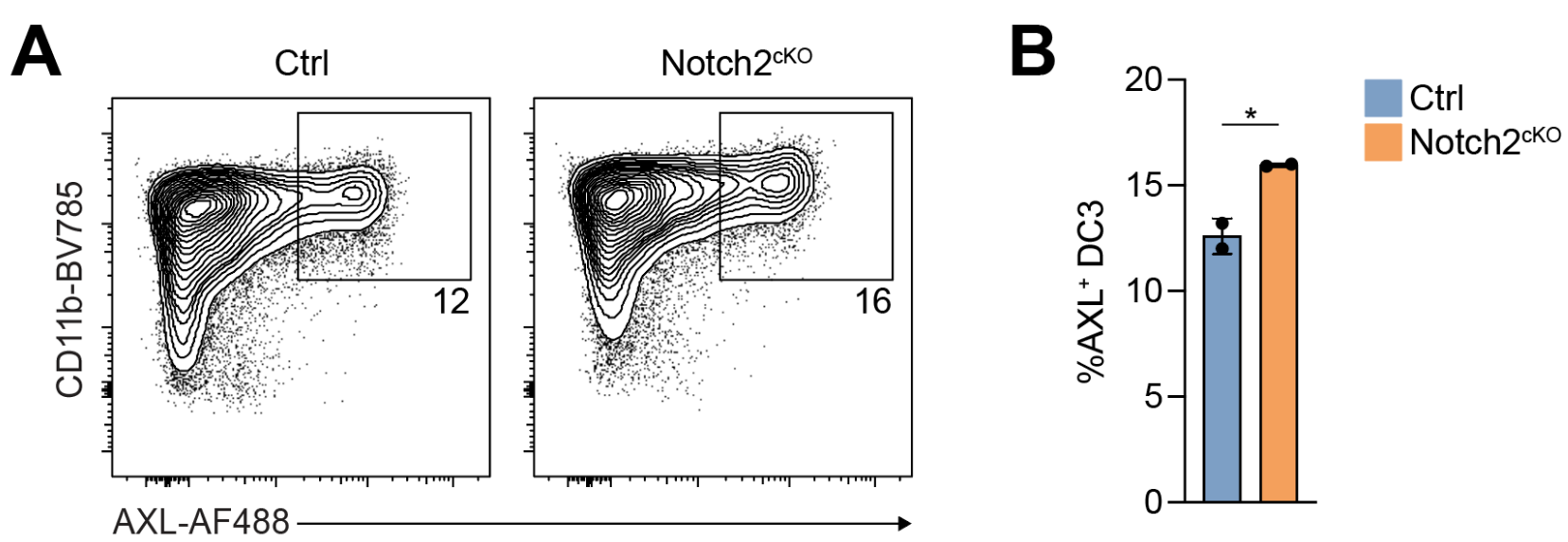

Fig. R1. Shown is the quantification of AXL$^+$DC3, pre-gated as CD11c$^+$MHCII$^+$CD26$^+$XCR1$^-$, in a Flt3L stimulated BM culture after 7 days.

While this does not exclude cytokine redundancy, previous studies (e.g., Kingston et al., Blood 2009; Durai et al., JEM 2018) suggest that alternative or compensatory pathways can contribute to DC development in the absence of Flt3L. Taken together, we agree that the precise ontogeny of this population remains to be definitively established. However, the combined fate-mapping, phenotypic, and functional data argue against a purely CCR2-dependent monocytic origin and instead support alignment with the DC3 lineage. **We revised the manuscript to clarify these points and to more explicitly acknowledge the remaining uncertainties**.

Assessing dependence on growth and survival signals is useful for defining cellular identity. Therefore, the authors should examine whether this population depends on CSF1R signaling. https://www.sciencedirect.com/science/article/pii/S1074761320304532

We fully agree with the reviewer that assessing dependence on growth and survival signals is important for defining cellular identity, and that CSF1R signaling represents a relevant axis in this context. However, direct interrogation of CSF1R dependence in the intestinal setting is challenging

to interpret. CSF1R is broadly expressed across multiple myeloid populations, including various macrophage subsets that are abundant in the gut and play key roles in maintaining tissue homeostasis. As a result, genetic or antibody-mediated perturbation of CSF1R signaling would not selectively target the population of interest, but instead would broadly affect the intestinal myeloid compartment. Indeed, previous work (e.g., from the Powrie laboratory) has shown that perturbation of intestinal macrophages profoundly alters tissue homeostasis and can itself lead to secondary inflammatory and microbiota changes, complicating interpretation.

For these reasons, we did not include CSF1R perturbation experiments, as they would not allow us to attribute observed effects specifically to the inf-DC3 population. Instead, we addressed lineage and identity using a combination of complementary approaches, including Ms4a3-Cre fate mapping, and single and mixed bone marrow chimera experiments, together with phenotypic and transcriptional characterization. **We agree that more selective tools to interrogate CSF1R dependence in defined dendritic cell subsets would be highly valuable, and their development will be important for future studies. We have clarified this limitation in the revised manuscript.**
